# Dietary quality among men and women in 187 countries in 1990 and 2010: a systematic assessment

**DOI:** 10.1016/S2214-109X(14)70381-X

**Published:** 2015-02-19

**Authors:** Fumiaki Imamura, Renata Micha, Shahab Khatibzadeh, Saman Fahimi, Peilin Shi, John Powles, Dariush Mozaffarian, on behalf of the Global Burden of Diseases Nutrition and Chronic Diseases Expert Group (NutriCoDE)

**Affiliations:** a Medical Research Council Epidemiology Unit, Institute of Metabolic Science, University of Cambridge School of Clinical Medicine, Cambridge Biomedical Campus, Cambridge, UK; b Department of Food Science and Human Nutrition, Agricultural University of Athens, Athens, Greece; c Gerald J and Dorothy R Friedman School of Nutrition Science and Policy, Tufts University, Boston, MA, USA; d Department of Epidemiology, Harvard School of Public Health, Boston, MA, USA; e Department of Public Health and Primary Care, Cambridge Institute of Public Health, Cambridge, UK

## Abstract

**Background:**

Healthy dietary patterns are a global priority to reduce non-communicable diseases. Yet neither worldwide patterns of diets nor their trends with time are well established. We aimed to characterise global changes (or trends) in dietary patterns nationally and regionally and to assess heterogeneity by age, sex, national income, and type of dietary pattern.

**Methods:**

In this systematic assessment, we evaluated global consumption of key dietary items (foods and nutrients) by region, nation, age, and sex in 1990 and 2010. Consumption data were evaluated from 325 surveys (71·7% nationally representative) covering 88·7% of the global adult population. Two types of dietary pattern were assessed: one reflecting greater consumption of ten healthy dietary items and the other based on lesser consumption of seven unhealthy dietary items. The mean intakes of each dietary factor were divided into quintiles, and each quintile was assigned an ordinal score, with higher scores being equivalent to healthier diets (range 0–100). The dietary patterns were assessed by hierarchical linear regression including country, age, sex, national income, and time as exploratory variables.

**Findings:**

From 1990 to 2010, diets based on healthy items improved globally (by 2·2 points, 95% uncertainty interval (UI) 0·9 to 3·5), whereas diets based on unhealthy items worsened (−2·5, −3·3 to −1·7). In 2010, the global mean scores were 44·0 (SD 10·5) for the healthy pattern and 52·1 (18·6) for the unhealthy pattern, with weak intercorrelation (*r*=–0·08) between countries. On average, better diets were seen in older adults compared with younger adults, and in women compared with men (p<0·0001 each). Compared with low-income nations, high-income nations had better diets based on healthy items (+2·5 points, 95% UI 0·3 to 4·1), but substantially poorer diets based on unhealthy items (−33·0, −37·8 to −28·3). Diets and their trends were very heterogeneous across the world regions. For example, both types of dietary patterns improved in high-income countries, but worsened in some low-income countries in Africa and Asia. Middle-income countries showed the largest improvement in dietary patterns based on healthy items, but the largest deterioration in dietary patterns based on unhealthy items.

**Interpretation:**

Consumption of healthy items improved, while consumption of unhealthy items worsened across the world, with heterogeneity across regions and countries. These global data provide the best estimates to date of nutrition transitions across the world and inform policies and priorities for reducing the health and economic burdens of poor diet quality.

**Funding:**

The Bill & Melinda Gates Foundation and Medical Research Council.

## Introduction

Poor quality of diet is a major cause of mortality and disability worldwide.^1^ International food programmes have traditionally focused on food security and micronutrient deficiency, but the diet-related health burdens due to non-communicable chronic diseases (NCDs) are now surpassing those due to undernutrition in nearly every region of the world.^1^, ^2^, ^3^, ^4^ This trend has raised the global concern of a so-called nutrition transition and convergence toward less healthy diets globally, with growing attention on the need to improve transnational food policies and overall diets.^5^, ^6^, ^7^ However, the differences in dietary patterns across the world, and how such dietary patterns are changing with time, are not well established. An improved understanding of dietary patterns and changes around the world is crucial to inform, design, and implement strategies to reduce national and global diet-related diseases.^1^, ^8^

Most previous global analyses of diet have relied on national-level estimates of food availability (food balance sheets) from the UN Food and Agricultural Organization (FAO) or on similar industry-derived data for national imports and exports or sales.^9^, ^10^, ^11^, ^12^, ^13^ However, such estimates might have large errors with respect to actual national intakes and cannot assess within-country differences across key population subgroups—eg, by age or sex.^13^ Other previous studies of global diets have assessed only small subsets of nations.^14^ Therefore, absence of data and understanding of dietary patterns across the world greatly restricts informed setting of dietary policies and priorities.

Additionally, most analyses of dietary patterns have summed together greater consumption of more healthy items (eg, fruits and fish) and less consumption of unhealthy items (eg, sodium).^13^ Yet, the intakes of healthy versus unhealthy dietary factors might not be concordant across countries—for example, the Japanese population consumes high volumes of both fish and sodium.^15^, ^16^ Little is known about dietary patterns across the world based on consumption of healthier foods and nutrients versus consumption of unhealthy foods and nutrients.

We aimed to characterise global changes (or trends) in dietary patterns nationally and regionally and to assess heterogeneity by age, sex, national income, and type of dietary pattern. We analysed global dietary information derived from individual-based national surveys as part of our work of the Global Burden of Diseases Nutrition and Chronic Diseases Expert Group (NutriCoDE).

## Methods

### Global dietary consumption by country, age, sex, and time

Our methods for selection of key dietary factors, identification of surveys, and data extraction and analysis have been reported.^1^, ^15^, ^16^, ^17^ Briefly, in our systematic assessment, we focused on 20 foods and nutrients having at least probable or convincing evidence of effects on major NCDs, including cardiovascular diseases, diabetes, and diet-related cancers.^1^, ^17^, ^18^ We systematically searched, identified, and compiled data from nationally representative dietary surveys, large subnational surveys (when national surveys were not available), and UN FAO food balance sheets; for sodium intake, we additionally identified surveys assessing urinary sodium.^15^ In total, we compiled information from 325 dietary surveys, including 233 that were nationally representative, covering 88·7% of the global adult population, of which 154 were undertaken before 2000, with no significant difference in response rate across years; and on urinary sodium from 142 surveys (representing 71·9% of the global adult population).^15^, ^16^

For every survey, we obtained and assessed information about survey methods and population characteristics, and extracted or (in most cases) obtained data directly from the survey authors for dietary intakes by age, sex, and time.^15^, ^16^, ^17^, ^18^, ^19^ We additionally compiled, for all 187 nations, year-specific data for national availability of ten foods and ten nutrients. In view of our aim to assess NCDs, we focused on data from adults (aged ≥20 years) only.^17^

We evaluated dietary intakes adjusted for a 2000 kcal per day (8·37 MJ per day) diet^15^, ^16^, ^17^ to assess diet quality independently of diet quantity, and to reduce measurement error within and across surveys (because energy intake is related to under-reporting or over-reporting of dietary consumption and adjustment for total energy intake partly corrects the error).^20^ For all dietary factors, we developed an age-integrating Bayesian hierarchical model that estimated the mean intake levels and its statistical uncertainty for each age-sex-country-year stratum, accounting for differences in dietary data, survey methods, representativeness, and sampling and modelling uncertainty.^15^, ^16^, ^17^, ^21^ Our dataset included estimates of dietary consumption for 26 subgroups (men and women and 13 age categories from 20–24·9 years to ≥80 years) within all 187 countries with a year 2000 population greater than 50 000^1^ in 1990 and 2010, covering 4·42 billion adults across 21 world regions.

### Characterisation of dietary patterns

For our analysis, we evaluated 17 of the 20 dietary factors compiled,^17^, ^18^ excluding three factors (calcium [we assessed milk instead]; seafood omega-3s [we assessed fish instead]; and fruit juice, with its equivocal evidence for effects on major health outcomes). We modelled two different dietary patterns: one based on relatively high consumption of ten healthy items (fruits, vegetables, beans and legumes, nuts and seeds, whole grains, milk, total polyunsaturated fatty acids, fish, plant omega-3s, and dietary fibre); and another based on relatively low consumption of seven unhealthy items (unprocessed red meats, processed meats, sugar-sweetened beverages, saturated fat, trans fat, dietary cholesterol, and sodium). For comparison, we also modelled a third overall dietary pattern that incorporated all 17 dietary factors together.

To derive a score for each pattern, the mean age-specific, sex-specific, and nation-specific intakes of each dietary factor in 2010 were divided into quintiles, based on all 4862 age-specific, sex-specific, and country-specific strata. Each quintile was assinged an ordinal score. Higher scores were given to quintiles with higher mean intakes of healthier foods (1 to 5 points). Of unhealthier foods, higher scores were given to quintiles with lower mean intakes (5 to 1 points). For each population stratum, scores across different dietary items were summed to obtain the total score for each of three dietary patterns: healthy items, unhealthy items, and all items combined. For comparability, every score was standardised to a 100-point scale (higher scores equivalent to healthier diets). To optimise comparability of trends over time, the quintile cutpoints for every dietary factor in 2010 were used to generate quintile cutpoints for every dietary item in 1990.

### Statistical analysis

Each dietary pattern was assessed by country, sex, age, and national income.^19^ For these analyses, we modelled hierarchical linear regression in which age and sex strata were nested within every nation and random intercepts were estimated.^22^ To estimate national, regional, and global means, each age and sex stratum was weighted by the proportion of adults within each contributing country. Every model included age, sex, and national income simultaneously to assess whether any of these key sociodemographic factors were independently associated with the dietary pattern score. To test linear trends in dietary patterns across age and national income, ordinal categories of age and income were assessed as continuous variables. Similar models were used to test trends in dietary patterns from 1990 to 2010, after standardisation of age and sex distributions to 2010 to assess changes independent of varying demographics with time.

Statistical uncertainty was quantified with Monte Carlo simulations.^1^, ^15^, ^16^, ^21^ We simultaneously propagated the uncertainty in the estimated dietary intake of all items in every age, sex, country, and time stratum by randomly drawing from the 95% uncertainty interval (UI) of intake and combining results across 1000 iterations. The 95% UIs were derived from estimated SEs based on within-iteration and between-iteration variances.^23^ Using the median and SE, we evaluated Wald statistics (square of β/SE) to test the null hypothesis for each result from the regression analyses.

### Role of the funding source

The funders had no role in the study design, study conduct, data analysis, data interpretation, or writing of the report. All authors had full access to all the data in the study and take responsibility for the integrity of the data and the accuracy of the data analysis. The corresponding author had final responsibility to submit the report for publication.

## Results

In 2010, consumption levels of key foods and nutrients related to NCDs varied across their quintile categories by between two-fold to more than 50-fold (table 1, appendix pp 2–41). The largest variation was noted for mean wholegrain consumption (10th to 90th percentiles: 12–157 g per day), fruit juice (1·4–86 g per day), nuts and seeds (1·5–19·4 g per day), beans and legumes (1·6–147 g per day), milk (33–230 g per day), seafood omega-3 fats (22–553 mg per day), plant omega-3 fats (0·2–1·5 g per day), sugar-sweetened beverages (33–293 g per day), and processed meats (3·9–34 g per day). Smaller but still substantial variation was seen for saturated fat, trans fat, cholesterol, and sodium. Between the 17 dietary factors contributing to dietary patterns, correlations across countries were moderate or weak (*r*=–0·44 to 0·48).

**Table 1:** Dietary consumption of selected foods and nutrients among men and women in 187 countries in 2010

	Quintiles determined by all age-specific, sex-specific, and country-specific estimates (n estimates=4862)*				
	1st	2nd	3rd	4th	5th
**Healthy items**					
Wholegrains, g per day	12 (1·0–18)	24 (19–31)	40 (31–56)	70 (56–89)	157 (89–477)
Fruits, g per day	57 (17–72)	88 (72–101)	114 (101–131)	151 (131–174)	204 (174–395)
Fruit juices, g per day†‡	1·4 (0·0–4·8)	10 (4·9–18)	27 (18–36)	48 (36–62)	86 (62–298)
Vegetables, g per day	73 (24–95)	109 (95–119)	130 (119–144)	160 (144–182)	222 (182–463)
Fish, g per day	11 (4·8–15)	18 (15–22)	26 (22–30)	35 (30–41)	52 (41–99)
Nuts and seeds, g per day	1·5 (0·1–2·3)	3·1 (2·3–4·0)	5·1 (4·0–6·8)	9·5 (6·8–12·5)	19·4 (12·5–192)
Beans and legumes, g per day	1·6 (0·1–7·1)	14 (7·1–20)	27 (20–35)	57 (35–97)	147 (97–472)
Milk, g per day †	33 (7–56)	76 (56–103)	123 (103–141)	160 (141–188)	230 (188–470)
Dietary fibre, g per day	14 (7–16)	18 (16–19)	21 (19–22)	24 (22–26)	28 (26–41)
Polyunsaturated fat, % energy	2·8 (1·1–3·4)	4·0 (3·5–4·4)	4·9 (4·4–5·3)	5·9 (5·3–6·5)	7·9 (6·5–12·9)
Seafood omega-3, mg per day fat‡	22 (3·7–40)	56 (40–70)	95 (70–141)	215 (141–322)	553 (322–5202)
Plant omega-3 fat, g per day	0·2 (0·0–0·4)	0·5 (0·4–0·6)	0·7 (0·6–0·8)	1·1 (0·8–1·2)	1·5 (1·2–5·7)
Calcium, mg per day‡	399 (288–461)	506 (461–553)	611 (553–658)	711 (658–786)	883 (786–1272)
**Unhealthy items**					
Sugar-sweetened beverages, g per day†	33 (6·0–45)	57 (45–69)	85 (69–105)	137 (105–195)	293 (196–1239)
Unprocessed red meats, g per day	23 (2·6–28)	34 (28–40)	47 (40–53)	60 (53–71)	84 (71–138)
Processed meats, g per day	3·9 (1·8–5·1)	6·7 (5·2–9·2)	12 (9·2–16)	20 (16–26)	34 (26–76)
Saturated fat, % energy	7·1 (2·2–8·4)	9·1 (8·4–9·9)	11 (9·9–12·0)	13·2 (12·0–14·1)	16·7 (14·1–28·2)
Trans fat, % energy	0·6 (0·2–0·7)	0·8 (0·7–0·9)	1·0 (0·9–1·0)	1·1 (1·0–1·3)	1·6 (1·3–6·8)
Cholesterol, mg per day	182 (93–204)	220 (204–236)	250 (236–264)	281 (264–296)	321 (297–455)
Sodium, g per day	2·3 (1·4–2·6)	2·9 (2·6–3·1)	3·5 (3·1–3·7)	4·0 (3·7–4·2)	4·6 (4·2–6·4)

Data are the median (range) of mean consumption levels in each quintile.

* Combining estimates of mean consumption levels across 13 age categories from 20–24·9 to >80 years in 5-year increments, men and women, and 187 countries.

† To convert units to servings per day, divide by 226·8 (8 oz).

‡ Fruit juice and calcium were not included in the calculation of diet pattern scores because of equivocal evidence for effects of fruit juice on major health outcomes and because calcium consumption was highly correlated with milk consumption (Spearman *r*=0·75), which was already included in the diet pattern. Similarly, consumption of seafood omega-3 polyunsaturated fatty acid (PUFA) was not included in the calculation of diet pattern scores because of high correlation with fish consumption (*r*=0·80).

The mean (SD, range) global dietary pattern scores out of a maximum (healthiest) of 100 were 44·0 (10·5, 13·8–64·5) on the basis of ten healthy foods and nutrients, 52·1 (18·6, 15·2–93·4) on the basis of seven unhealthy foods and nutrients, and 51·9 (9·3, 27·5–75·3) on the basis of all 17 foods and nutrients (table 2, appendix pp 42–45). As expected, both the healthy pattern score and the unhealthy pattern score were moderately associated with the overall score (Spearman *r*=0·63 for healthy pattern score; *r*=0·70 for unhealthy pattern score). By contrast, the healthy pattern and unhealthy pattern had very little intercorrelation across countries (*r*=–0·08, p=0·14).

**Table 2:** Global dietary patterns among men and women in 187 countries in 2010

		**Score based on greater consumption of ten healthy dietary items**	**Score based on lesser consumption of seven unhealthy dietary items**	**Score based on 17 dietary items**
Global		44·0 (10·5)	52·1 (18·6)	51·9 (9·3)
Sex				
	Men	42·4 (10·5)	50·6 (18·8)	50·3 (9·4)
	Women	46·0 (10·6)	53·8 (18·5)	53·7 (9·3)
	p value*	<0·0001	<0·0001	<0·0001
Age, years				
	20–29	36·0 (10·0)	45·8 (18·5)	44·0 (9·4)
	30–39	39·4 (10·3)	46·3 (18·6)	46·5 (9·6)
	40–49	42·2 (10·7)	47·9 (18·7)	49·0 (9·7)
	50–59	44·4 (10·7)	50·4 (18·4)	51·5 (9·4)
	60–69	45·9 (10·7)	53·2 (18·1)	53·6 (9·0)
	70–79	45·6 (10·8)	54·0 (18·0)	53·7 (8·9)
	≥80	44·7 (10·7)	54·2 (18·0)	53·2 (8·9)
	p value for trend*	<0·0001	<0·0001	<0·0001
Country income level				
	High (n=47)	47·0 (9·3)	37·4 (11·2)	48·6 (8·1)
	Upper middle (n=53)	45·2 (11·3)	46·2 (12·8)	50·1 (8·7)
	Lower middle (n=51)	40·9 (10·9)	55·0 (15·3)	51·1 (9·4)
	Low (n=36)	42·9 (9·6)	75·9 (12·5)	59·9 (7·3)
	p value for trend*	0·0005	<0·0001	0·0006

Data are mean (SD). Possible range of each score is from 0 (less healthy) to 100 (more healthy).

* p values for differences by sex or across ordinal categories of age or country income were estimated using hierarchical regression analysis accounting for age–sex distribution. Age, sex, and country income (high, ≥US$12 475; upper middle, US$4037–12 474; lower middle, US$1025–4036; low, <US$1024) were mutually adjusted when assessing statistical significance of each.

For all three patterns, older adults had better dietary patterns than did younger adults (table 2). On average, women also had better dietary patterns than did men. Conversely, substantial differences were noted across the three dietary patterns by national income. Higher national income was associated with better quality for the healthy dietary pattern, accounting for 15·7% of between-country variability of the score (p=0·0005), and with much worse quality for the unhealthy dietary pattern, accounting for 46·9% of between-country variability (p<0·0001). Compared with low-income countries, high-income countries had higher healthy dietary pattern scores (adjusted mean difference 2·5, 95% UI 0·3–4·1), but substantially lower unhealthy dietary pattern scores. Unhealthy dietary pattern scores were also substantially lower in upper middle-income countries (−25·2; 95% UI −30·2 to −20·2) and lower middle-income (−18·5, −23·7 to −13·2) countries than in low-income countries. In post-hoc analysis, high-income nations showed a non-significant positive correlation between the two types of pattern scores (*r*=0·27), whereas low-income nations showed an inverse correlation (*r*=–0·24; p>0·05 each; p_interaction_>0·1 by national income). These differences between healthy and unhealthy foods were largely masked when only one overall dietary pattern score was assessed.

**Figure 1:**
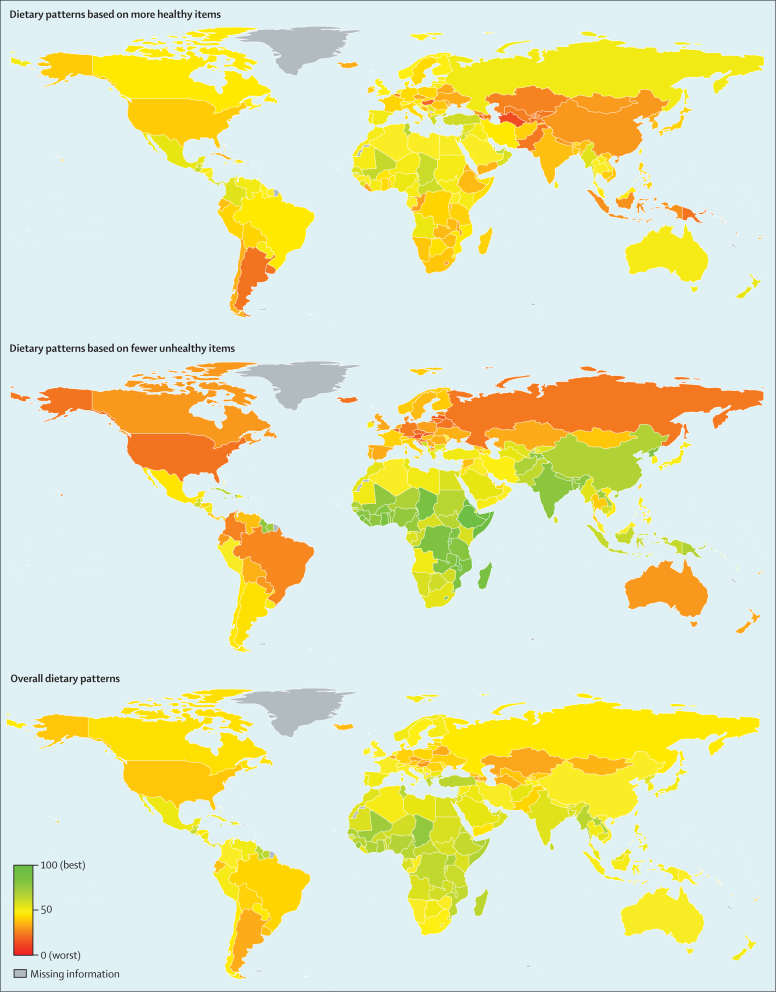
Global dietary patterns among men and women in 187 countries in 2010 Values represent degrees of adherence to each dietary pattern, ranging from 0 (least healthy) to 100 (most healthy).

**Figure 2:**
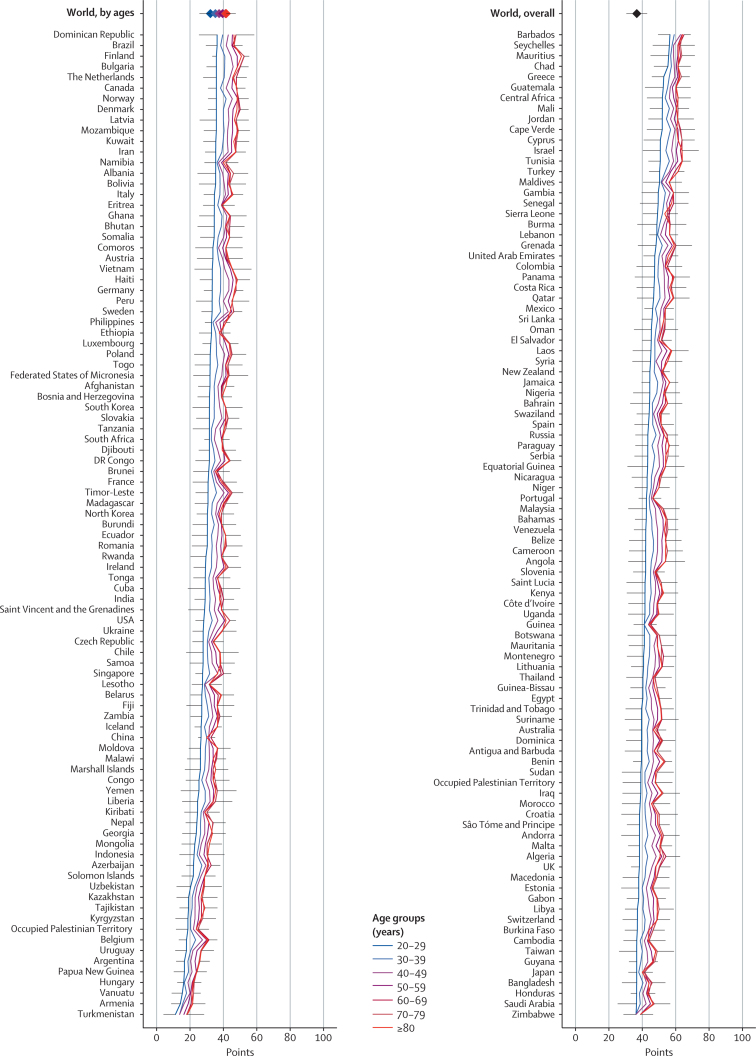
Dietary pattern among men and women in 187 countries in 2010 based on greater consumption of ten more healthy items Values represent degrees of adherence to each dietary pattern, ranging from 0 (least healthy) to 100 (most healthy). 187 countries are ordered by scores among adults aged 20–29 years. Lines show error bars for each country, which represent the lower side of the 95% uncertainty interval for the lowest age-specific estimate and the upper side of the 95% uncertainty interval for the highest age-specific estimate.

**Figure 3:**
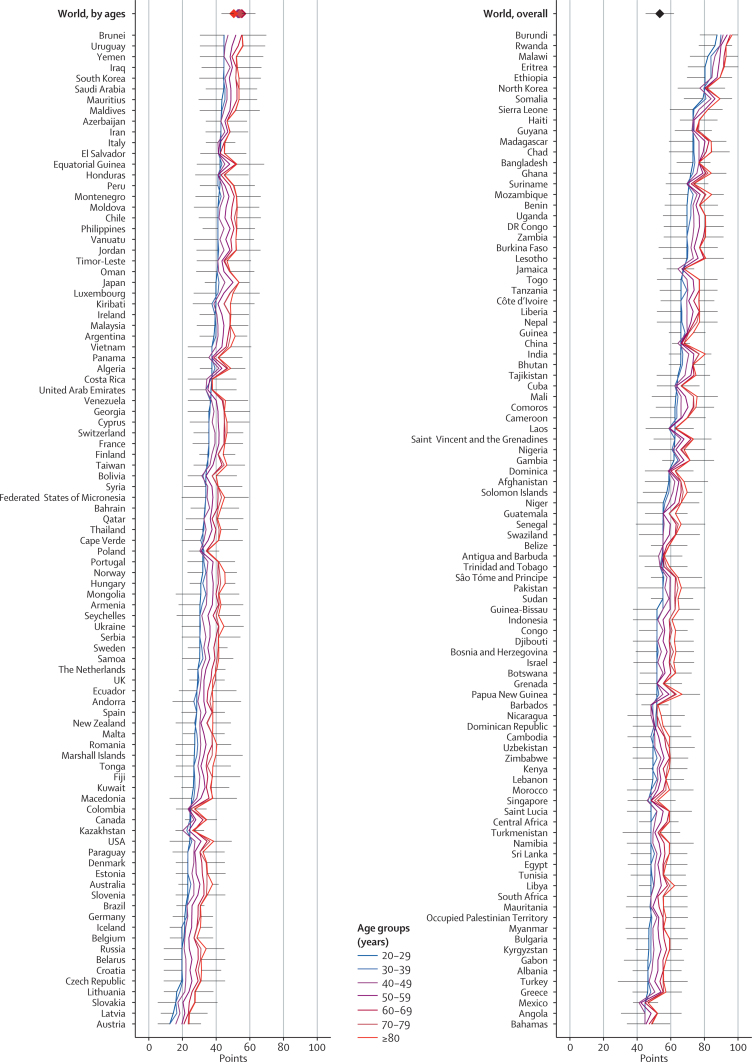
Dietary pattern among men and women in 187 countries in 2010 based on less consumption of seven unhealthy items Values represent degrees of adherence to each dietary pattern, ranging from 0 (least healthy) to 100 (most healthy). 187 countries are ordered by scores among adults aged 20–29 years. Lines show error bars for each country, which represent the lower side of the 95% uncertainty interval for the lowest age-specific estimate and the upper side of the 95% uncertainty interval for the highest age-specific estimate.

Substantial heterogeneity was evident in diet quality across nations, and comparisons across countries also varied substantially for the healthy versus unhealthy diet patterns (Figure 1, Figure 2, Figure 3, appendix pp 42–45). As noted in analyses by national income, this divergence of national diet quality based on healthy versus unhealthy items was largely masked when only overall diet patterns were considered. For example, India ranked 70th of 187 countries for the overall diet pattern (50·6 points, 95% UI 45·5–56·0), but ranked high (23rd) for the score based on fewer unhealthy items (70·0, 63·0–77·0) and ranked low (149th) for the score based on more healthy items (33·8, 27·4–40·4). Similar trends were noted in many low-income countries in southeast Asia and sub-Saharan Africa.

Dietary patterns often varied greatly even between neighbouring countries (figure 1, appendix p 46). For example, dietary patterns based on healthy items were poor in Argentina (20·8 points) but moderate in Brazil (40·7); whereas dietary patterns based on fewer unhealthy items were very poor in Brazil (24·3), but moderate in Argentina (42·4). Similar heterogeneity was evident between Caribbean neighbours (eg, Barbados and Dominica) and southeast Asian neighbours (eg, Laos and Thailand).

**Figure 4:**
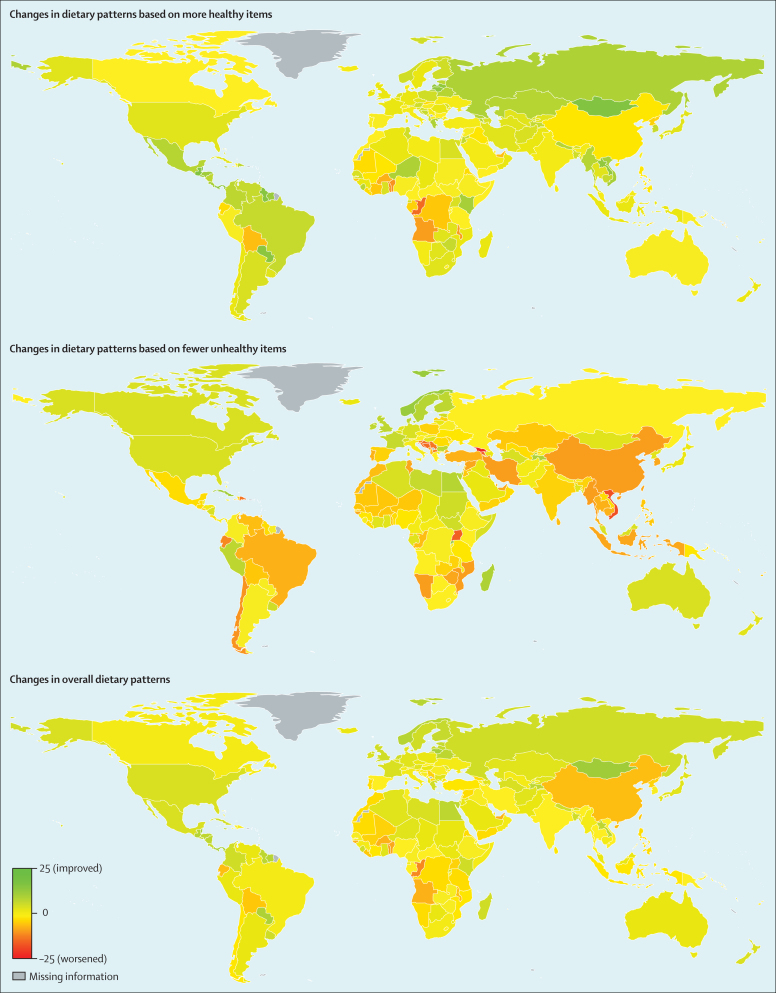
Changes in dietary patterns from 1990 to 2010 among men and women in 187 countries Top: changes in dietary pattern scores based on greater consumption of ten healthful foods and nutrients. Middle: changes in dietary pattern scores based on less consumption of seven unhealthful foods and nutrients. Bottom: changes in dietary pattern scores based on both healthful and unhealthful foods and nutrients. Values represent degrees of adherence to each dietary pattern, ranging from 0 (least healthful) to 100 (most healthful). Scores in 1990 were standardised to age and sex distribution in 2010.

Between 1990 and 2010, global dietary patterns based on more healthy items improved modestly (by 2·2 points, 95% UI 0·9–3·5; figure 4, appendix pp 47–51), indicating greater consumption of these more healthy foods and nutrients. By contrast, global dietary patterns based on fewer unhealthy items worsened (−2·5; 95% UI −3·3 to −1·7), indicating concomitant increased consumption of these unhealthy foods and nutrients. These trends were weakly correlated across countries (*r*=–0·08 overall, range −0·15 to 0·09 in the four national-income categories; p>0·05 each).

These trends did not significantly vary by age or sex (p>0·4 each), but significantly varied by national income (p<0·02 each; appendix p 47 figure S24). Nations with higher incomes had larger improvements in diet patterns based on healthy items than did nations with lower incomes; for example, by 2·5 points (95% UI 0·5–4·6) comparing high-income to low-income countries. By contrast, middle-income nations showed the largest worsening in diet patterns based on unhealthy items: compared with high-income nations, greater worsening by 2·5 points (95% UI 0·5–4·5) and by and 2·8 points (95% UI 0·9–4·8) was noted in upper-middle nations and lower-middle income nations, respectively. Although most world regions showed modest improvements in dietary patterns between 1990 and 2010 on the basis of more healthy items, such improvements were generally not noted in the poorest regions, including in sub-Saharan Africa and the Andean states of Latin America. Conversely, most regions of the world showed substantial declines in diet quality based on increased consumption of unhealthy items. The exceptions included many of the wealthiest regions including the USA and Canada, western Europe, Australia, and New Zealand, where consumption of these unhealthy items modestly decreased. Of note, for these world regions and nations, this improvement was superimposed on a poor starting score in 1990 (appendix pp 47–49 figure S24). Thus, despite some improvement by 2010, dietary scores for unhealthy items in wealthy countries remained among the worst in the world. As seen for absolute scores, most of these differences in national and regional trends were far less apparent when examining the dietary pattern aggregating both healthy and unhealthy dietary items (figure 4).

## Discussion

In this systematic assessment of different dietary patterns across 187 nations in 1990 and 2010, we noted that diet quality varied by age, sex, national income, time, and world region. Consumption of healthier foods and nutrients has modestly increased during the past two decades; however, consumption of unhealthy foods and nutrients has increased to a greater extent. Improvements in healthier foods were seen in high-income and middle-income countries; by contrast, no improvements were seen in the poorest regions. Notably, we identified the substantial variations of diets across the world depending on whether diet quality was characterised by greater consumption of healthier or lesser consumption of unhealthier foods and nutrients. This heterogeneity went largely undetected when diet quality was defined by aggregation of both healthy and unhealthy items. To our knowledge, this is the first investigation to analyse data derived from individual-based surveys and to evaluate current worldwide dietary patterns and their changes over time, providing the best estimates to-date of nutrition transitions across the world (panel).

Panel **Research in context**

**Systematic review**

We did not do any systematic search in the initial planning for this study. However, through extensive collaborations with experts of the UN's organisations and global health projects, we were aware of no global data derived from surveys assessing individuals’ diets. Therefore, there is no evidence on overall diet quality across the world derived from individuals’ diets, including their international variations, associations with key demographic variables, and trends with time. Moreover, evidence is absent for distinct types of dietary patterns based on healthy items versus unhealthy items, although these two classes of dietary factors are consumed differently across the world.

**Interpretation**

To our knowledge, this is the first study to evaluate dietary patterns among adults across the world. In 187 countries between 1990 and 2010, dietary patterns and their trends over time varied substantially depending on differences between healthy and unhealthy foods. The global variations were largely undetectable if we evaluated one scale of diet quality, as has been previously done.^13^, ^14^ Global public health should recognise diverse dietary trends based on healthy versus unhealthy foods, identify determinants of this diversity, and improve strategies for global, transnational, and domestic policy actions with a joint consideration of both healthy and unhealthy foods.

The 17 foods and nutrients included in this analysis are especially relevant for their effects on obesity and NCDs.^24^, ^25^ Suboptimum dietary patterns based on these factors are linked to substantial burdens of morbidity, premature mortality, and medical costs.^1^, ^2^ Indeed, it has been estimated that, by 2020, nearly 75% of all deaths and 60% of all disability-adjusted life years will be attributable to NCDs,^1^, ^26^ and most of the key causes of these conditions are dietary or strongly diet-related.^1^ Our results characterising dietary patterns across the world have implications for the reduction of disease and economic burdens of poor diet by lowering the consumption of unhealthier foods, increasing the consumption of healthier foods, or both.

Our findings also have implications for undernutrition. Whereas globally valid data for consumption levels of most micronutrients are not currently available, the healthy dietary factors included in our analysis are the major contributors to many essential nutrients associated with a range of health outcomes in both low-income and high-income nations.^27^ Recent research has shown associations between suboptimum dietary patterns and poor pregnancy and fetal growth outcomes.^28^, ^29^ Although caloric deficits and disease burdens other than those of NCDs must not be overlooked in some low-income countries,^1^, ^3^, ^11^ the trends in dietary patterns we note show the urgent need to focus on improvement of diet quality among poor populations worldwide. Left unaddressed, undernutrition and deficiency diseases will be rapidly eclipsed in these populations by obesity and NCDs, as is already occurring in India, China, and other middle-income nations.^1^, ^2^, ^3^, ^4^, ^11^ Notably, many of the differences by national income were minimised or not seen when examining the overall diet pattern that aggregated both healthy and unhealthy foods and nutrients. Similarly, the Prospective Urban Rural Epidemiology study, which used one overall diet pattern score—the Alternate Healthy Eating Index (AHEI)^13^—reported no significant association between national income and diet quality across 17 nations.^14^ Diet pattern scores such as the AHEI were originally developed to assess diet–disease associations within fairly homogeneous, high-income populations.^13^ Our novel findings show that associations between socioeconomic status and diet quality might vary substantially for diet patterns based on healthy versus unhealthy items, and also that such diet patterns are only weakly correlated. Different policies could be influencing the two dietary patterns—eg, transnational marketing and investment often promotes consumption of unhealthy foods, such as snacks in Thailand and soft drinks in Mexico,^5^, ^6^ whereas governmental strategies attempt to promote consumption of healthy food, such as the multifocal polices in Norway and nutrition education in South Korea.^5^, ^8^ When combined with assessment of nation-specific policies, our observations derived from individuals’ diets should help to understand and characterise influences of business, agriculture, and health policies on consumption of healthy food, consumption of unhealthy food, and population health in different countries.

Although a monotonic relation between wealth and diet quality has been frequently proposed,^30^ we noted high-income nations at both extremes of healthy dietary patterns. These global observations are supported by previous nation-specific findings that within-country socioeconomic status might correlate with either better or worse diets depending on the dietary factors in question.^11^, ^30^ For instance, in southern Europe, lower socioeconomic status is associated with higher consumption of fruits and vegetables, possibly reflecting greater domestic production in rural areas.^30^, ^31^ We identified substantial variation in both healthy and unhealthy diet patterns by national income, indicating much more complex relations between socioeconomic status and diet quality than has commonly been assumed.

Our data for improved global intakes of healthier foods between 1990 and 2010 are supported by country estimates of food availability.^5^, ^9^, ^11^ These improvements might be attributable to advances in agricultural practices, storage, transport, and out-of-season availability of healthier foods, as well as increased recognition of the importance of healthier foods to minimise NCDs.^11^ Yet, notably, improvements were not seen in many of the lowest-income nations. Causes of this disparity need to be fully characterised and might be multifaceted and region-specific. For instance, a failure to increase more healthy foods could reflect unguided economic transition, such as liberalisation and investment for marketing of unhealthy products in a wealthy segment of a population;^6^, ^11^ in northwest sub-Saharan Africa, for example, food prices have increased and diet quality has worsened.^32^ Domestic and international conflict could affect diets. For example, conflicts in the DR Congo (1996–2008) and neighbouring countries have impeded both food production and trade.^33^ Our work should help to link these possible economic and political factors to actual diets and to assess determinants of the potential divergence^6^ in consumption of healthy foods in the poorest nations in the world.

By contrast with improving global trends based on consumption of healthy foods, our findings show that the consumption of unhealthy foods has been worsening. Such trends have been speculated about previously^5^, ^8^, ^9^, ^11^, ^34^ and are now supported by our individual-level data. Yet, our findings indicate no single global convergence of nutrition transition into homogeneously unhealthy diets. Moreover, our findings suggest that not all nations have been increasing their intake of unhealthy foods to the same extent. Indeed, most high-income nations are actually showing reductions in consumption of unhealthy foods. Together with the increasing consumption of healthy foods, these results could at least in part explain the observed reductions in blood pressure, blood cholesterol, and cardiovascular mortality in the USA, Canada, and western Europe.^1^, ^4^ Yet, despite the improvements in dietary patterns in these high-income nations, our findings show that they are still among the worst in the world, especially for consumption of unhealthy foods.^1^, ^4^

Our investigation has several strengths. We included all available global data derived from individual-level dietary surveys, most of which were nationally representative, and further supplemented by FAO food balance sheets. Although not perfect, these data provide the most valid information so far about global dietary intakes. We included major dietary risk factors for NCDs, the leading causes of morbidity and mortality in the world. We assessed differences by country, age, sex, national income, and time; characterised diet patterns separately based on healthy items versus unhealthy items; and provided a novel demonstration of the divergent correlates and trends in these patterns.

Our study has several potential limitations. We did not assess within-country variations of diets and socioeconomic characteristics, and further studies should investigate how diet quality varies within countries during this time of global nutrition transition.^6^, ^11^ Globally valid and reliable information about other potentially relevant dietary factors, for example extent of food processing or glycaemic load, is not currently available.^34^ Yet these factors are inversely correlated with intakes of minimally processed foods such as the healthy items we evaluated, and so our patterns would at least partly capture differences in these other factors. Although we made extensive efforts to minimise bias and incorporate heterogeneity and uncertainty, individual-based data are subject to measurement errors, and were incomplete for some regions, dietary factors, and years. These limitations were incorporated into uncertainty in the analysis, but could cause sampling bias, information bias, or both. Dietary patterns were not derived through agnostic methods, such as factor analysis. Instead, we aimed to assess dietary patterns related to NCDs, rather than identify novel patterns. Although we distinguished between healthy and unhealthy items, different items within each category were equally weighted. Yet, each of these dietary factors are relevant for different NCDs and other conditions.

In conclusion, global diet quality varies substantially by age, sex, and national income, and fairly independent heterogeneity is evident for diet patterns based on eating more healthy versus fewer unhealthy foods and nutrients. Increases in unhealthy patterns are outpacing increases in healthy patterns in most world regions. In view of the disease burdens associated with suboptimum diet quality, these findings emphasise the need to better elucidate the societal, policy, and food industry determinants of these differences and trends, and to implement policies to address these inequities and improve diet quality globally.

## References

[1] Lim SS, Vos T, Flaxman AD (2012). A comparative risk assessment of burden of disease and injury attributable to 67 risk factors and risk factor clusters in 21 regions, 1990-2010: a systematic analysis for the Global Burden of Disease Study 2010. Lancet.

[2] Lopez AD, Mathers CD, Ezzati M, Jamison DT, Murray CJL, Lopez AD, Mathers CD, Ezzati M, Jamison DT, Murray CJL (2006). Global Burden of Disease and Risk Factors.

[3] De Onis M, Blössner M, Borghi E, Frongillo EA, Morris R (2004). Estimates of global prevalence of childhood underweight in 1990 and 2015. JAMA.

[4] Lozano R, Naghavi M, Foreman K (2012). Global and regional mortality from 235 causes of death for 20 age groups in 1990 and 2010: a systematic analysis for the Global Burden of Disease Study 2010. Lancet.

[5] Keats S, Wiggins S (2014). Future diets: implications for agriculture and food prices.

[6] Hawkes C (2006). Uneven dietary development: linking the policies and processes of globalization with the nutrition transition, obesity and diet-related chronic diseases. Global Health.

[7] Vandevijvere S, Monteiro C, Krebs-Smith SM (2013). Monitoring and benchmarking population diet quality globally: a step-wise approach. Obes Rev.

[8] Lachat C, Otchere S, Roberfroid D (2013). Diet and physical activity for the prevention of noncommunicable diseases in low- and middle-income countries: a systematic policy review. PLoS Med.

[9] Ezzati M, Riboli E (2013). Behavioral and dietary risk factors for noncommunicable diseases. N Engl J Med.

[10] Monteiro CA, Moubarac J-CC, Cannon G (2013). Ultra-processed products are becoming dominant in the global food system. Obes Rev.

[11] Kennedy G, Nantel G, Shetty P (2004). Globalization of food systems in developing countries: impact on food security and nutrition. FAO Food Nutr Pap.

[12] Basu S, Yoffe P, Hills N, Lustig RH (2013). The relationship of sugar to population-level diabetes prevalence: an econometric analysis of repeated cross-sectional data. PLoS One.

[13] Vandevijvere S, Monteiro C, Krebs-Smith SM (2013). Monitoring and benchmarking population diet quality globally: a step-wise approach. Obes Rev.

[14] Contribution O, Teo K, Lear S (2013). Prevalence of a healthy lifestyle among individuals with cardiovascular disease in high-, middle- and low-income countries: the Prospective Urban Rural Epidemiology (PURE) study. JAMA.

[15] Powles J, Fahimi S, Micha R (2013). Global, regional and national sodium intakes in 1990 and 2010: a systematic analysis of 24 h urinary sodium excretion and dietary surveys worldwide. BMJ Open.

[16] Micha R, Khatibzadeh S, Shi P (2014). Global, regional, and national consumption levels of dietary fats and oils in 1990 and 2010: a systematic analysis including 266 country-specific nutrition surveys. BMJ.

[17] Micha R, Kalantarian S, Wirojratana P (2012). Estimating the global and regional burden of suboptimal nutrition on chronic disease: methods and inputs to the analysis. Eur J Clin Nutr.

[18] Khatibzadeh S, Micha M, Afshin A, Rao M, Yakoob MY, Mozaffarian D (2012). Major dietary risk factors for chronic diseases: a systematic review of the current evidence for causal effects and effect Sizes. Circulation.

[19] World Bank (2013). World Development Indicators. http://databank.worldbank.org/data/download/WDI-2013-ebook.pdf.

[20] Willett WC, Willett WC (2012). Nutritional Epidemiology.

[21] Barendregt JJ, Van Oortmarssen GJ, Vos T, Murray CJ (2003). A generic model for the assessment of disease epidemiology: the computational basis of DisMod II. Popul Health Metr.

[22] Diez-Roux AV (2000). Multilevel analysis in public health research. Annu Rev Public Health.

[23] Gelman A, Rubin DB (1992). Inference from iterative simulation using multiple sequences. Stat Sci.

[24] World Cancer Research Fund/American Institute for Cancer Research (2007). Food, Nutrition, Physical Activity, and the Prevention of Cancer: a Global Perspective.

[25] Mozaffarian D, Bonow RO, Mann DL, Zipes DP, Peter L (2011). Braunwald's Heart Disease: A Textbook of Cardiovascular Medicine.

[26] Murray CJ, Lopez AD (1997). Global mortality, disability, and the contribution of risk factors: Global Burden of Disease Study. Lancet.

[27] McNeill S, Van Elswyk ME (2012). Red meat in global nutrition. Meat Science.

[28] Carmichael SL, Yang W, Feldkamp ML (2012). Reduced risks of neural tube defects and orofacial clefts with higher diet quality. Arch Pediatr Adolesc Med.

[29] Rodríguez-Bernal CL, Rebagliato M, Iñiguez C (2010). Diet quality in early pregnancy and its effects on fetal growth outcomes: the Infancia y Medio Ambiente (Childhood and Environment) Mother and Child Cohort Study in Spain. Am J Clin Nutr.

[30] Darmon N, Drewnowski A (2008). Does social class predict diet quality?. Am J Clin Nutr.

[31] Trichopoulou A, Naska A, Costacou T (2002). Disparities in food habits across Europe. Proc Nutr Soc.

[32] Lock K, Stuckler D, Charlesworth K, McKee M (2009). Potential causes and health effects of rising global food prices. BMJ.

[33] Ohiorhenuan JFE, Stewart F (2008). United Nations Development Programmeme. Post-Conflict Economic Recovery Enabling Local Ingenuity.

[34] Monteiro CA, Moubarac J-CC, Cannon G (2013). Ultra-processed products are becoming dominant in the global food system. Obes Rev.

