# Supplementary material for: Dietary quality among men and women in 187 countries in 1990 and 2010: a systematic assessment
Source: Lancet Glob Health. 2015 Feb 19;3(3):e132–42. doi: 10.1016/S2214-109X(14)70381-X (PMC4342410; doi:10.1016/S2214-109X(14)70381-X)
Supplement: Supplementary appendix [file mmc1.pdf]

THE LANCET Global Health

Supplementary appendix

This appendix formed part of the original submission and has been peer reviewed. We post it as supplied by the authors.

Supplement to: Imamura F, Micha R, Khatibzadeh S, et al, on behalf of the Global Burden of Diseases Nutrition and Chronic Diseases Expert Group (NutriCoDE). Dietary quality among men and women in 187 countries in 1990 and 2010: a systematic assessment. *Lancet Glob Health* 2015; **3:** e132–42.

Supplementary Materials for "Dietary quality among men and women in 187 countries in 1990 and 2010"

Fumiaki Imamura, Shahab Khatibzadeh, Renata Micha, Saman Fahimi, Peilin Shi, John Powles, Dariush Mozaffarian, on behalf of the Global Burden of Diseases Nutrition and Chronic Diseases Expert Group (NutriCoDE)

	Page
**Figure S1.** Whole grain consumption among men and women aged 20 years or older in 187 countries.	2
**Figure S2.** Fruit consumption among men and women aged 20 years or older in 187 countries.	4
**Figure S3.** Fruit juice consumption among men and women aged 20 years or older in 187 countries.	6
**Figure S4.** Vegetable consumption among men and women aged 20 years or older in 187 countries.	8
**Figure S5.** Fish consumption among men and women aged 20 years or older in 187 countries.	10
**Figure S6.** Consumption of nuts and seeds among men and women aged 20 years or older in 187 countries.	12
**Figure S7.** Consumption of beans and legumes among men and women aged 20 years or older in 187 countries.	14
**Figure S8.** Milk consumption among men and women aged 20 years or older in 187 countries.	16
**Figure S9.** Dietary fibre consumption among men and women aged 20 years or older in 187 countries.	18
**Figure S10.** Polyunsaturated fat consumption among men and women aged 20 years or older in 187 countries.	20
**Figure S11.** Consumption of seafood omega-3 fatty acids among men and women aged 20 years or older in 187 countries.	22
**Figure S12.** Consumption of plant omega-3 fatty acids among men and women aged 20 years or older in 187 countries.	24
**Figure S13.** Calcium consumption among men and women aged 20 years or older in 187 countries.	26
**Figure S14.** Sugar sweetened beverage consumption among men and women aged 20 years or older in 187 countries.	28
**Figure S15.** Unprocessed red meat consumption among men and women aged 20 years or older in 187 countries.	30
**Figure S16.** Processed meat consumption among men and women aged 20 years or older in 187 countries.	32
**Figure S17.** Saturated fat consumption among men and women aged 20 years or older in 187 countries.	34
**Figure S18.** Consumption of trans fatty acids among men and women aged 20 years or older in 187 countries.	36
**Figure S19.** Dietary cholesterol consumption among men and women aged 20 years or older in 187 countries.	38
**Figure S20.** Sodium consumption among men and women aged 20 years or older in 187 countries.	40
**Figure S21.** Dietary patterns based on consumption of more healthful foods and nutrients among men and women aged 20 years or older in 187 countries.	42
**Figure S22.** Dietary patterns based on consumption of lesser unhealthful foods and nutrients among men and women aged 20 years or older in 187 countries.	44
**Figure S23.** Global dietary patterns of 187 countries in 21 world regions.	46
**Figure S24.** Dietary patterns in 1990 and change from 1990 to 2010 among men and women by demographics and world regions.	47
**Figure S25.** Dietary patterns in 1990 among men and women in 187 countries.	48
**Figure S26.** Changes in dietary patterns from 1990 to 2010 among men and women in 187 countries.	50
**Collaborator list**	52

Correspondence:
Fumiaki Imamura, MS, PhD,
Medical Research Council Epidemiology Unit
Institute of Metabolic Science,
University of Cambridge School of Clinical Medicine,
Cambridge Biomedical Campus, Cambridge, CB2 0QQ, United Kingdom.
Email: fumiaki.imamura@mrc-epid.cam.ac.uk

Whole grain consumption among men

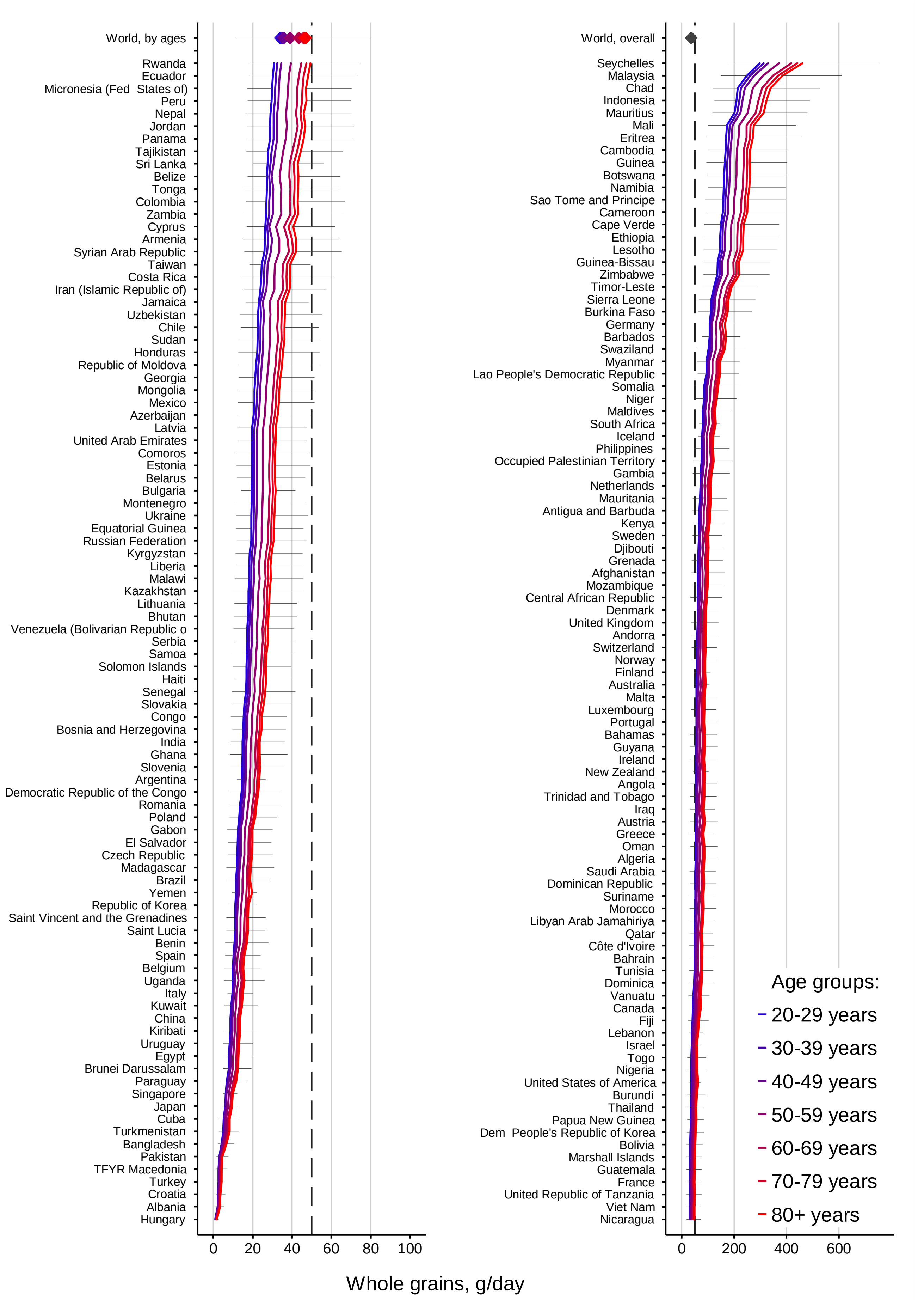

Figure S1. Whole grain consumption among men and women aged 20 years or older in 187 countries. Countries are ordered by the mean consumption levels among men and women with 20-29 years of age, from the lowest at the bottom-left to the highest at the top-right. Error bars for each country represent a lower side of 95% uncertainty interval (UI) for the lowest estimate and an upper side of 95% UI for the highest estimate. The dashed vertical line represents mean of the theoretical minimal risk exposure distribution for whole grain consumption.

Whole grain consumption among women

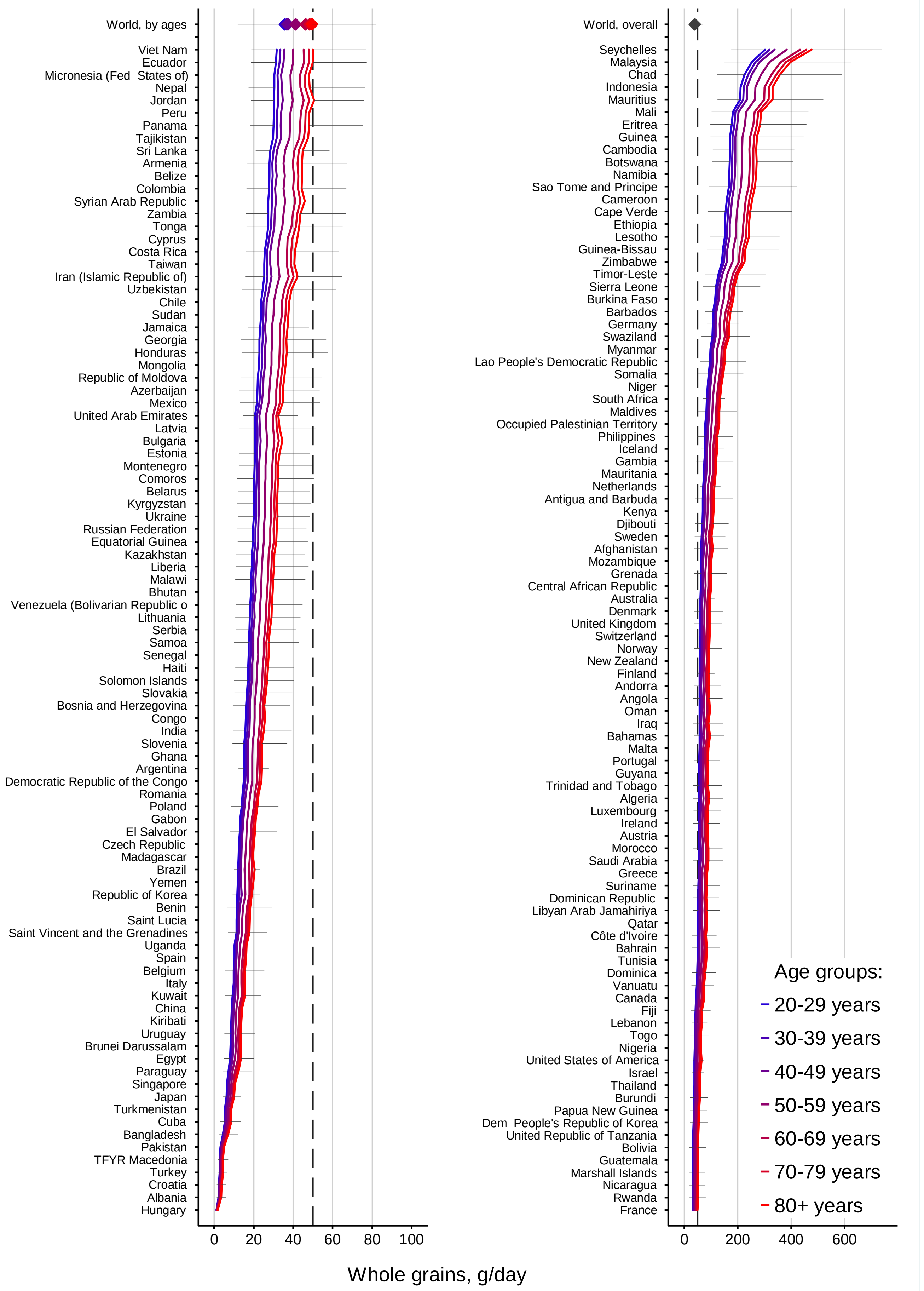

Fruit consumption among men

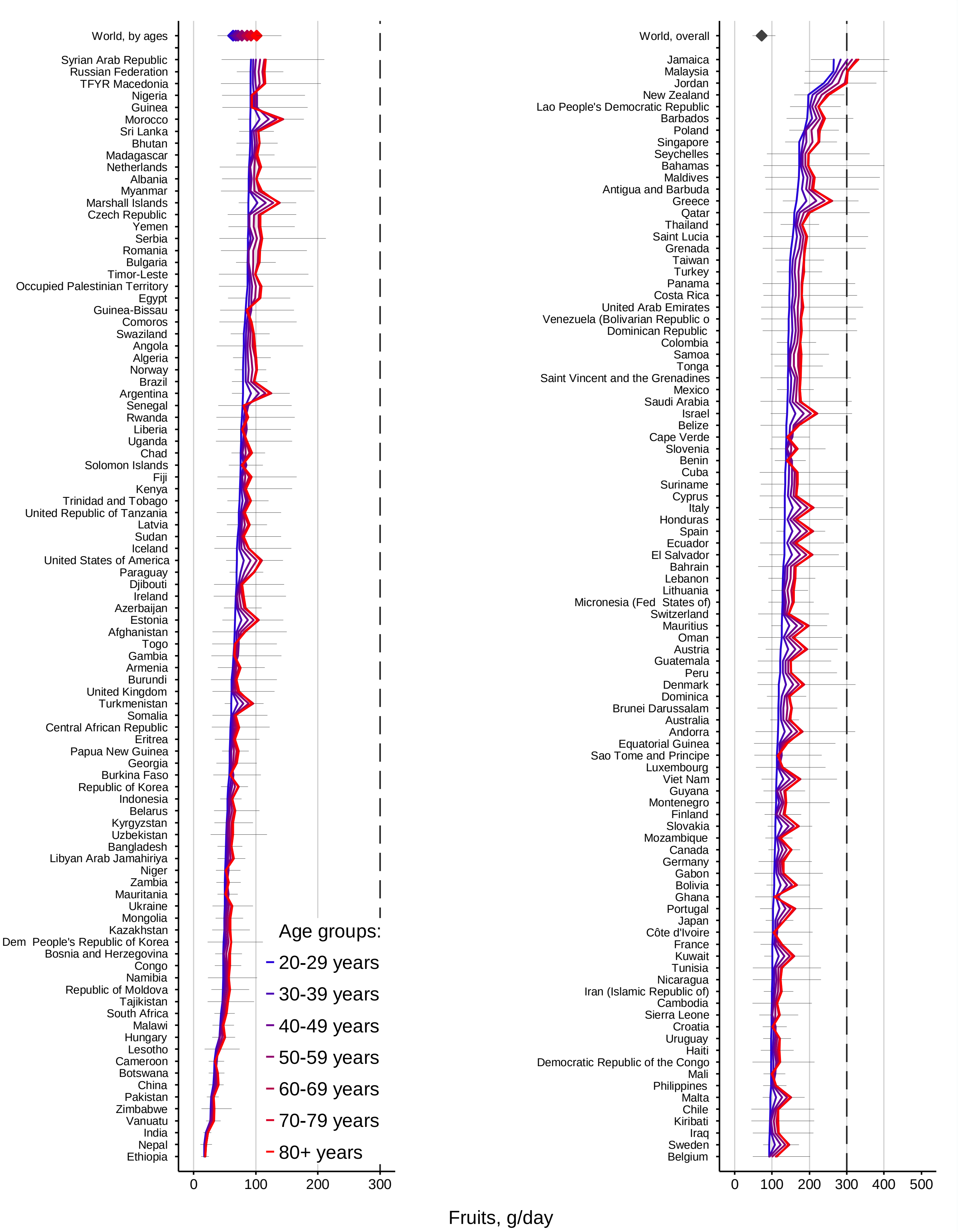

Figure S2. Fruit consumption among men and women aged 20 years or older in 187 countries.
Countries are ordered by the mean consumption levels among men and women with 20-29 years of age, from the lowest at the bottom-left to the highest at the top-right. Error bars for each country represent a lower side of 95% uncertainty interval (UI) for the lowest estimate and an upper side of 95% UI for the highest estimate. The dashed vertical line represents mean of the theoretical minimal risk exposure distribution for fruit consumption.

Fruit consumption among women

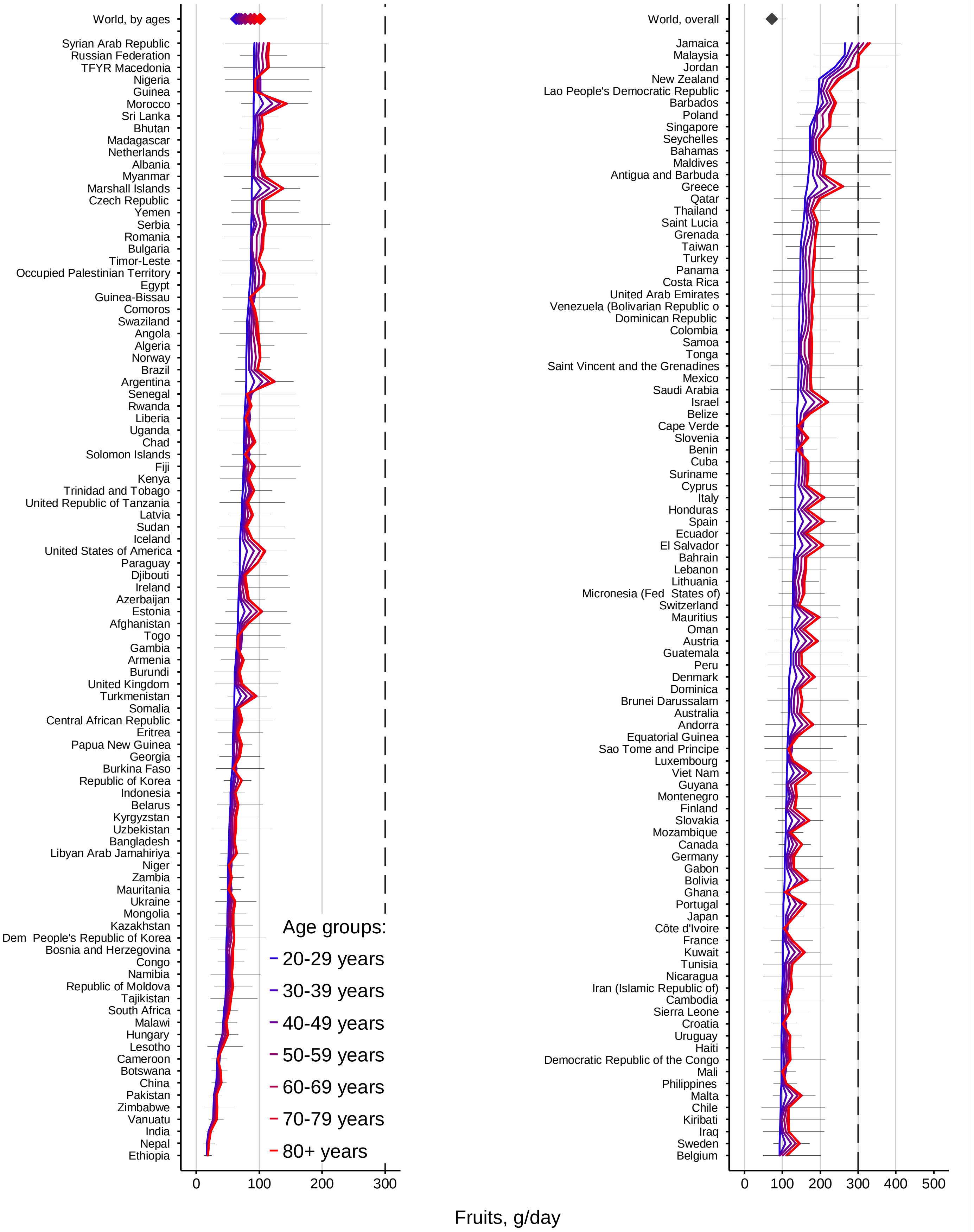

Fruit juice consumption among men

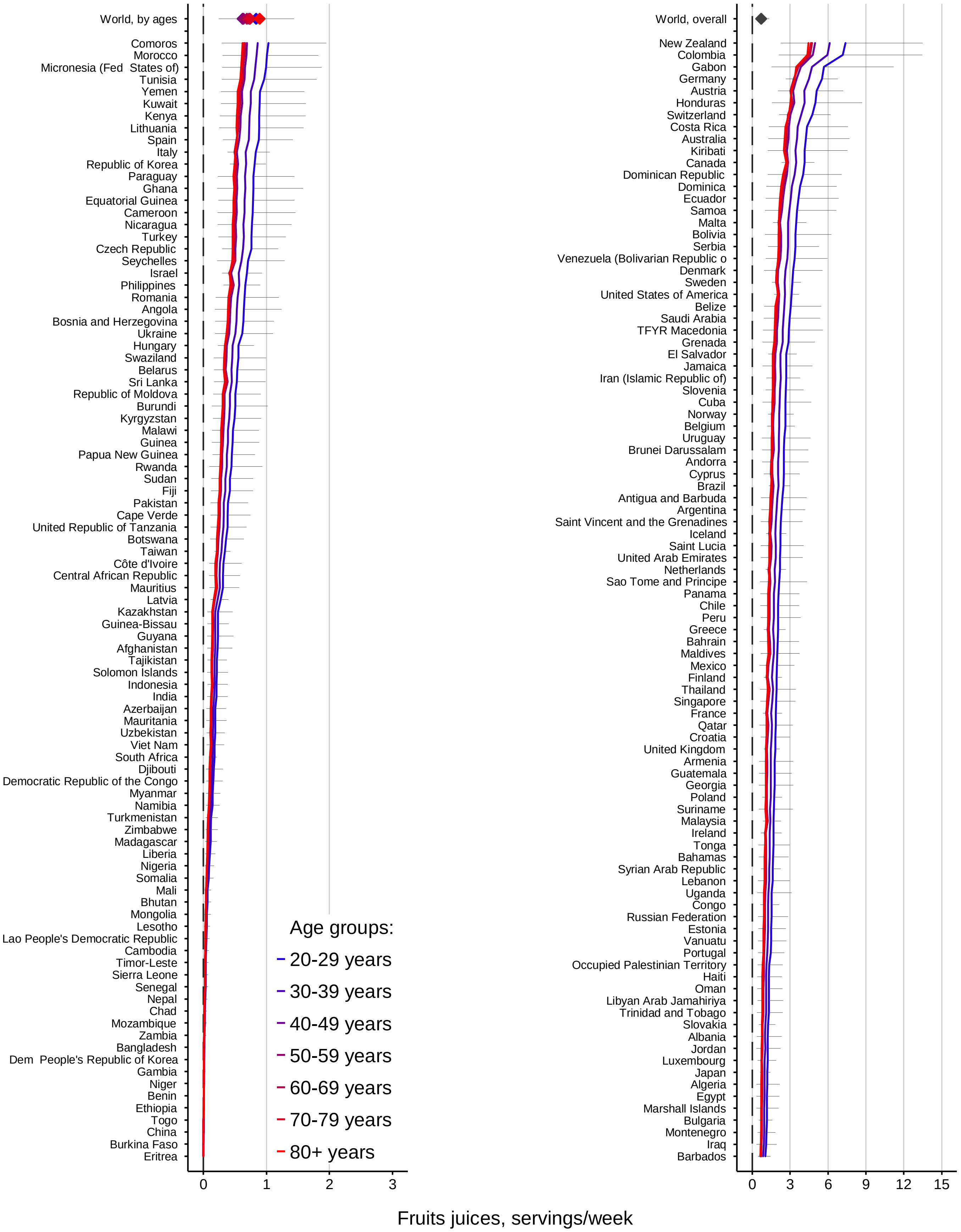

Figure S3. Fruit juice consumption among men and women aged 20 years or older in 187 countries.
Countries are ordered by the mean consumption levels among men and women with 20-29 years of age, from the lowest at the bottom-left to the highest at the top-right. Error bars for each country represent a lower side of 95% uncertainty interval (UI) for the lowest estimate and an upper side of 95% UI for the highest estimate. The dashed vertical line represents mean of the theoretical minimal risk exposure distribution for fruit juice consumption.

Fruit juice consumption among women

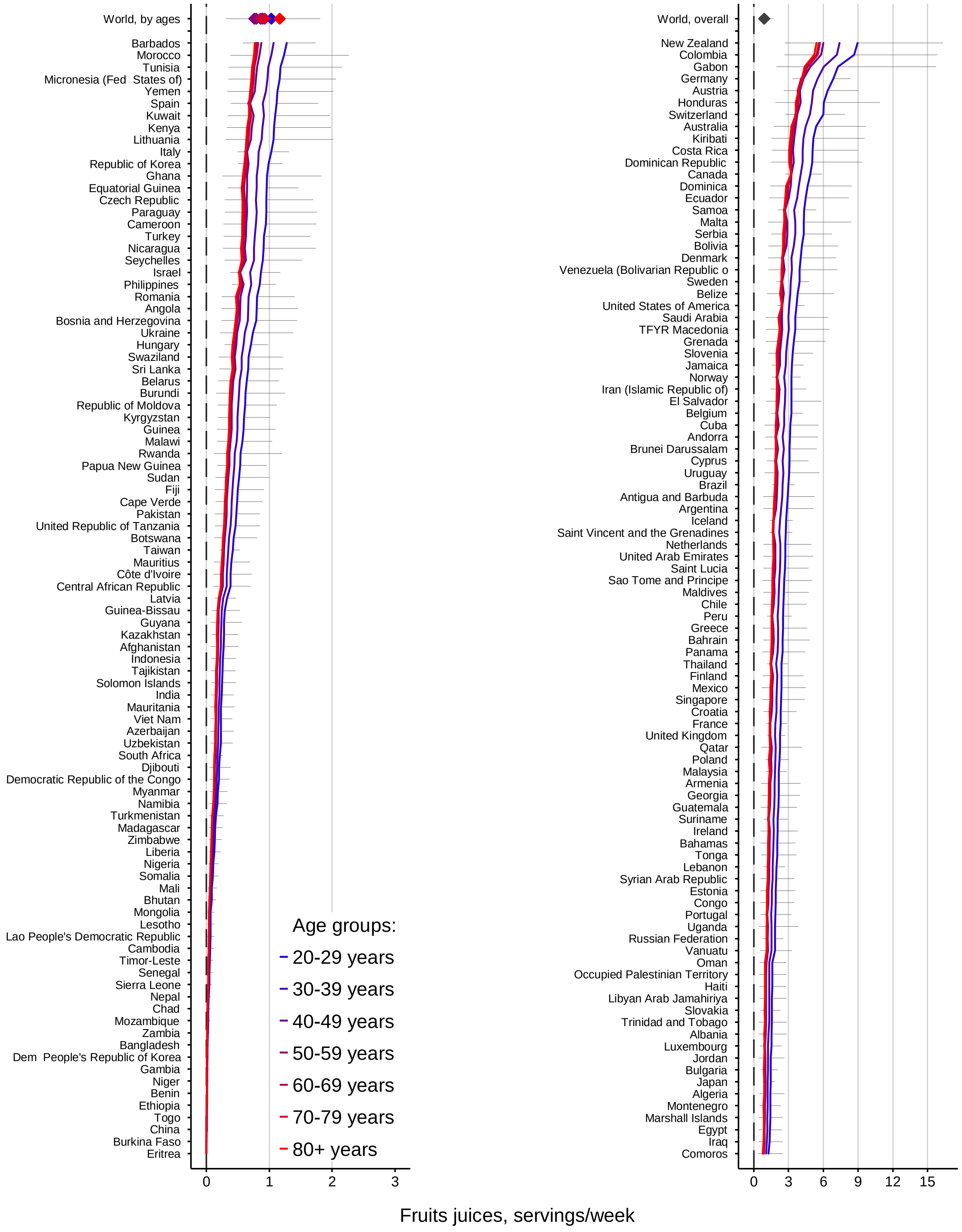

Vegetable consumption among men

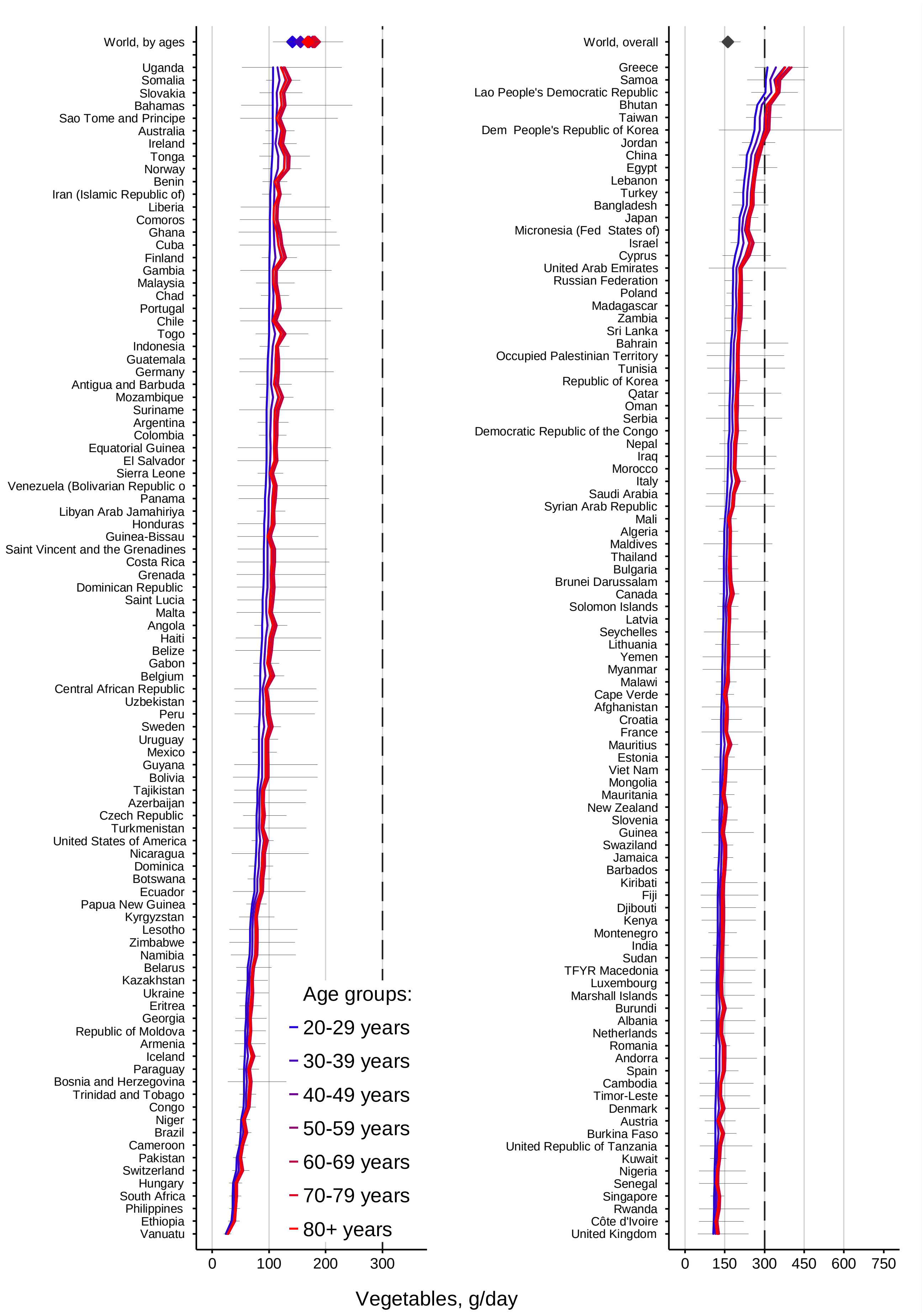

Figure S4. Vegetable consumption among men and women aged 20 years or older in 187 countries.
Countries are ordered by the mean consumption levels among men and women with 20-29 years of age, from the lowest at the bottom-left to the highest at the top-right. Error bars for each country represent a lower side of 95% uncertainty interval (UI) for the lowest estimate and an upper side of 95% UI for the highest estimate. The dashed vertical line represents mean of the theoretical minimal risk exposure distribution for vegetable consumption.

Vegetable consumption among women

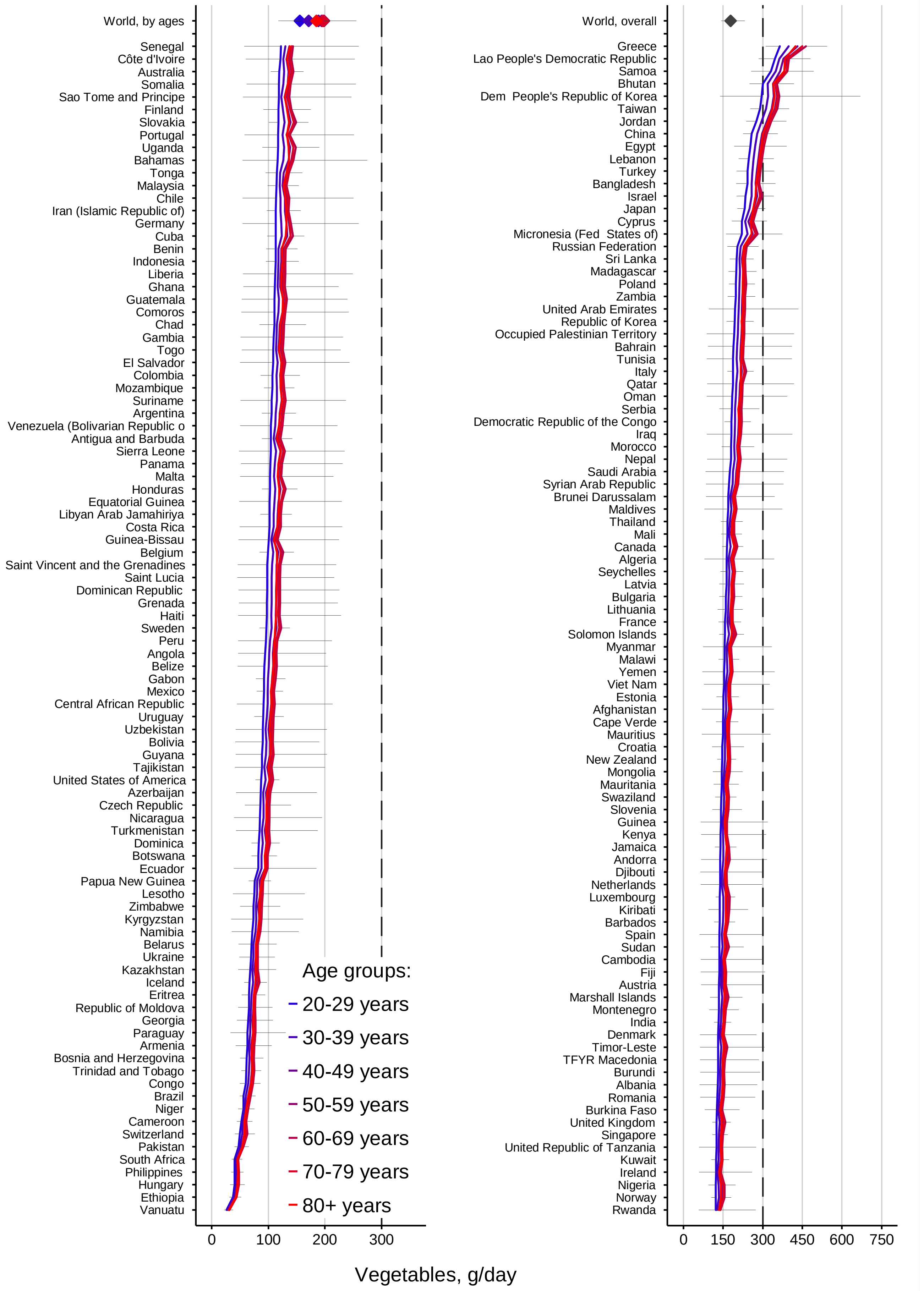

Fish consumption among men

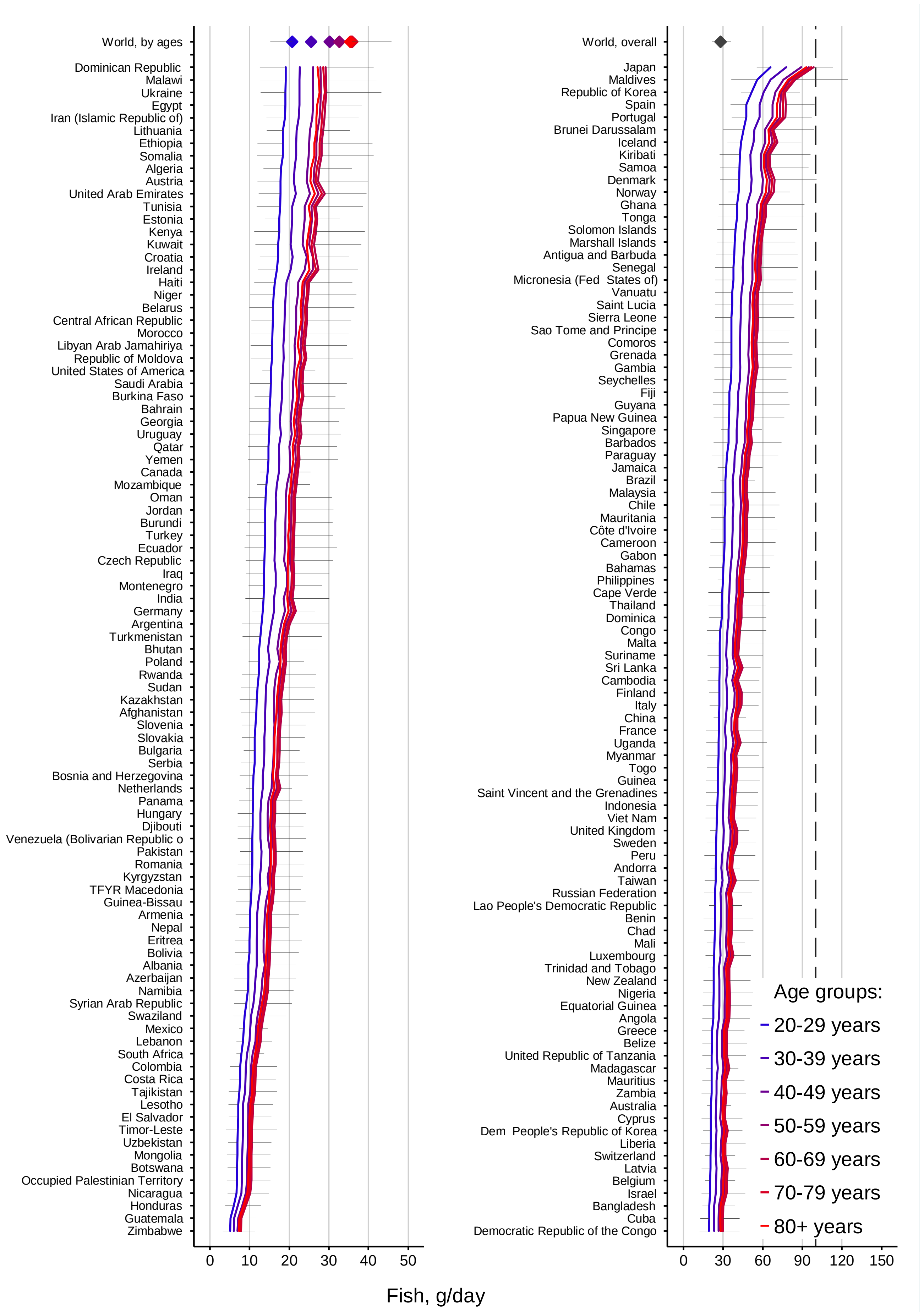

Figure S5. Fish consumption among men and women aged 20 years or older in 187 countries.
Countries are ordered by the mean consumption levels among men and women with 20-29 years of age, from the lowest at the bottom-left to the highest at the top-right. Error bars for each country represent a lower side of 95% uncertainty interval (UI) for the lowest estimate and an upper side of 95% UI for the highest estimate. The dashed vertical line represents mean of the theoretical minimal risk exposure distribution for fish consumption.

Fish consumption among women

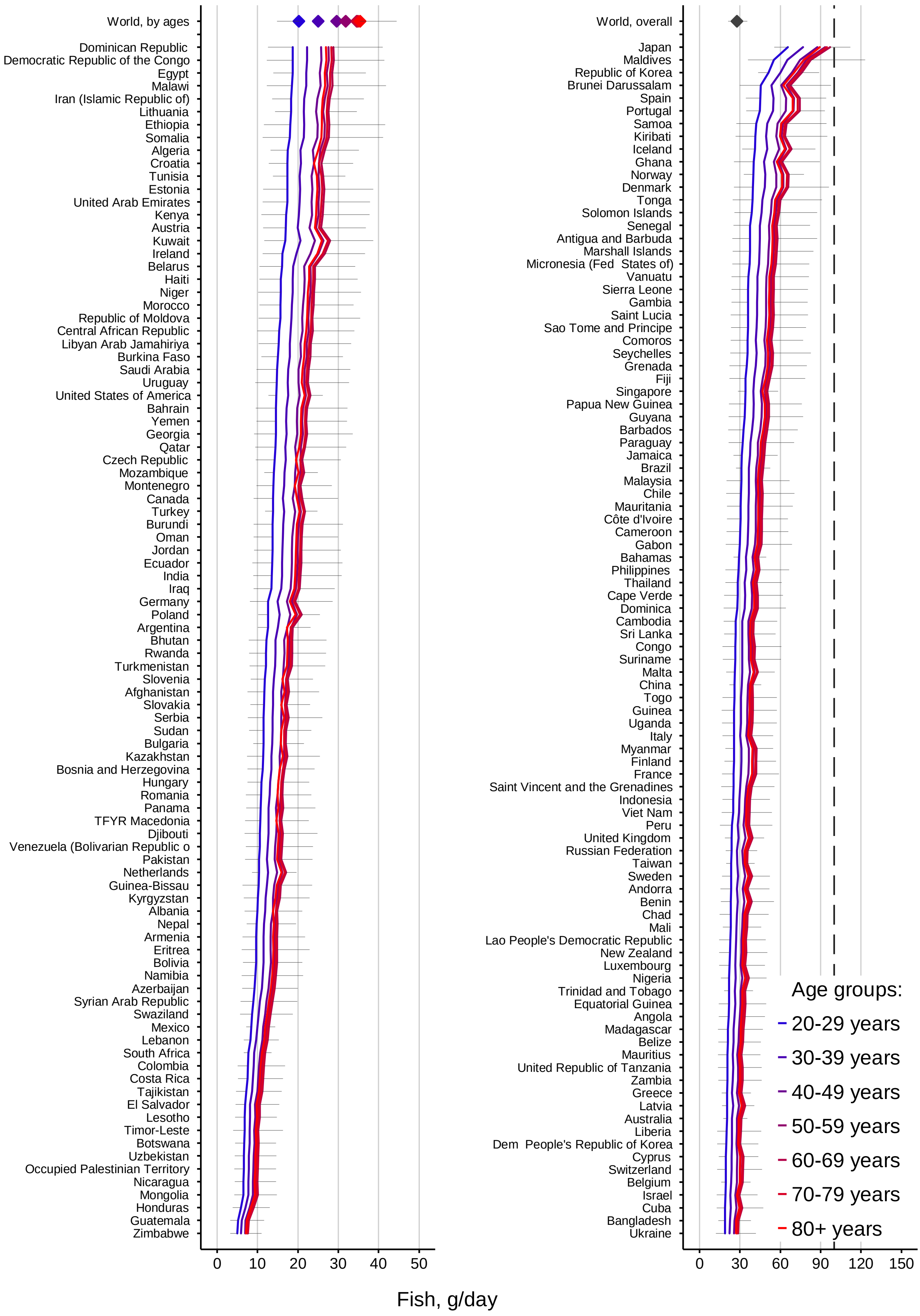

Consumption of nuts and seeds among men

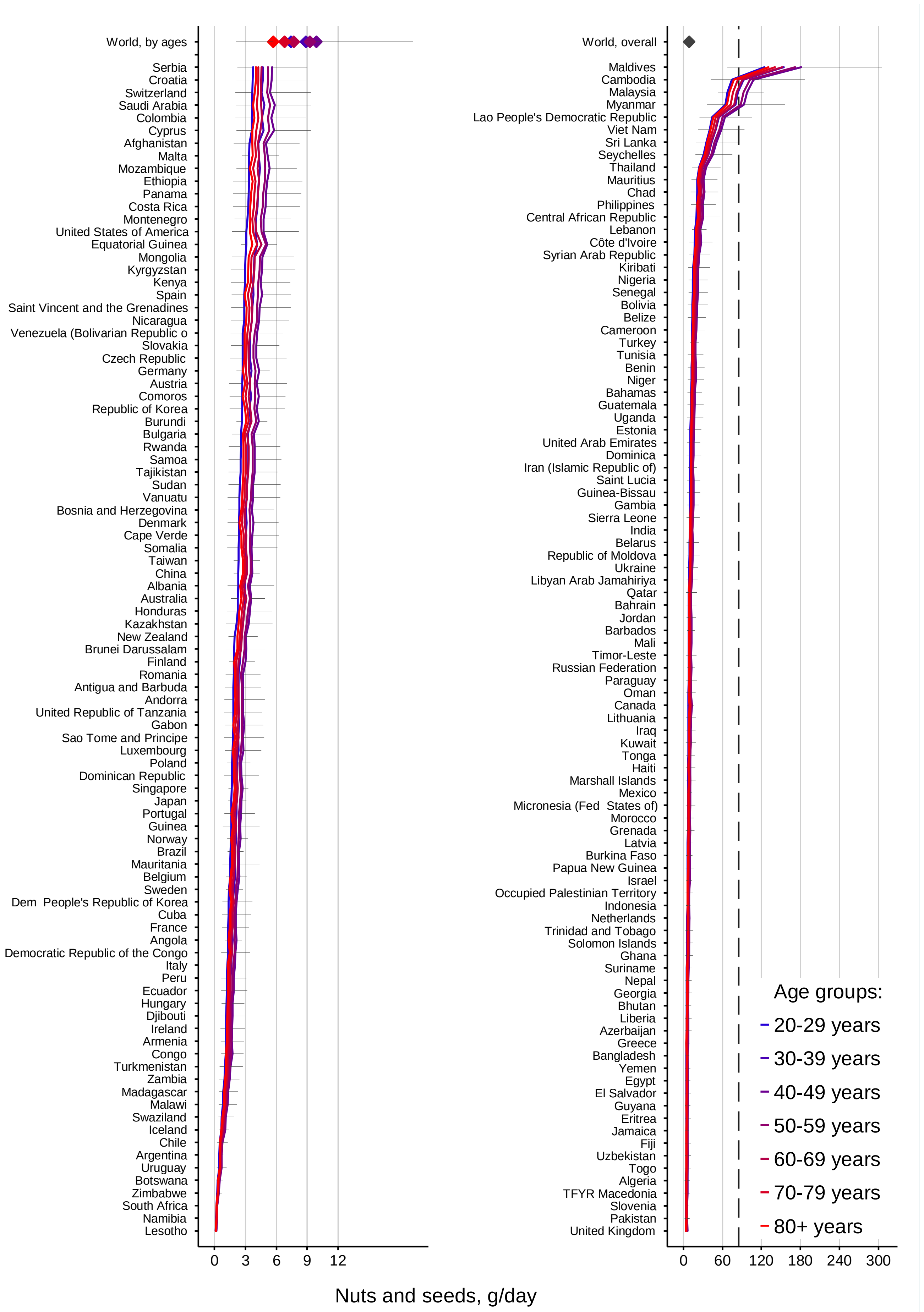

Figure S6. Consumption of nuts and seeds among men and women aged 20 years or older in 187 countries. Countries are ordered by the mean consumption levels among men and women with 20-29 years of age, from the lowest at the bottom-left to the highest at the top-right. Error bars for each country represent a lower side of 95% uncertainty interval (UI) for the lowest estimate and an upper side of 95% UI for the highest estimate. The dashed vertical line represents mean of the theoretical minimal risk exposure distribution for consumption of nuts and seeds.

Consumption of nuts and seeds among women

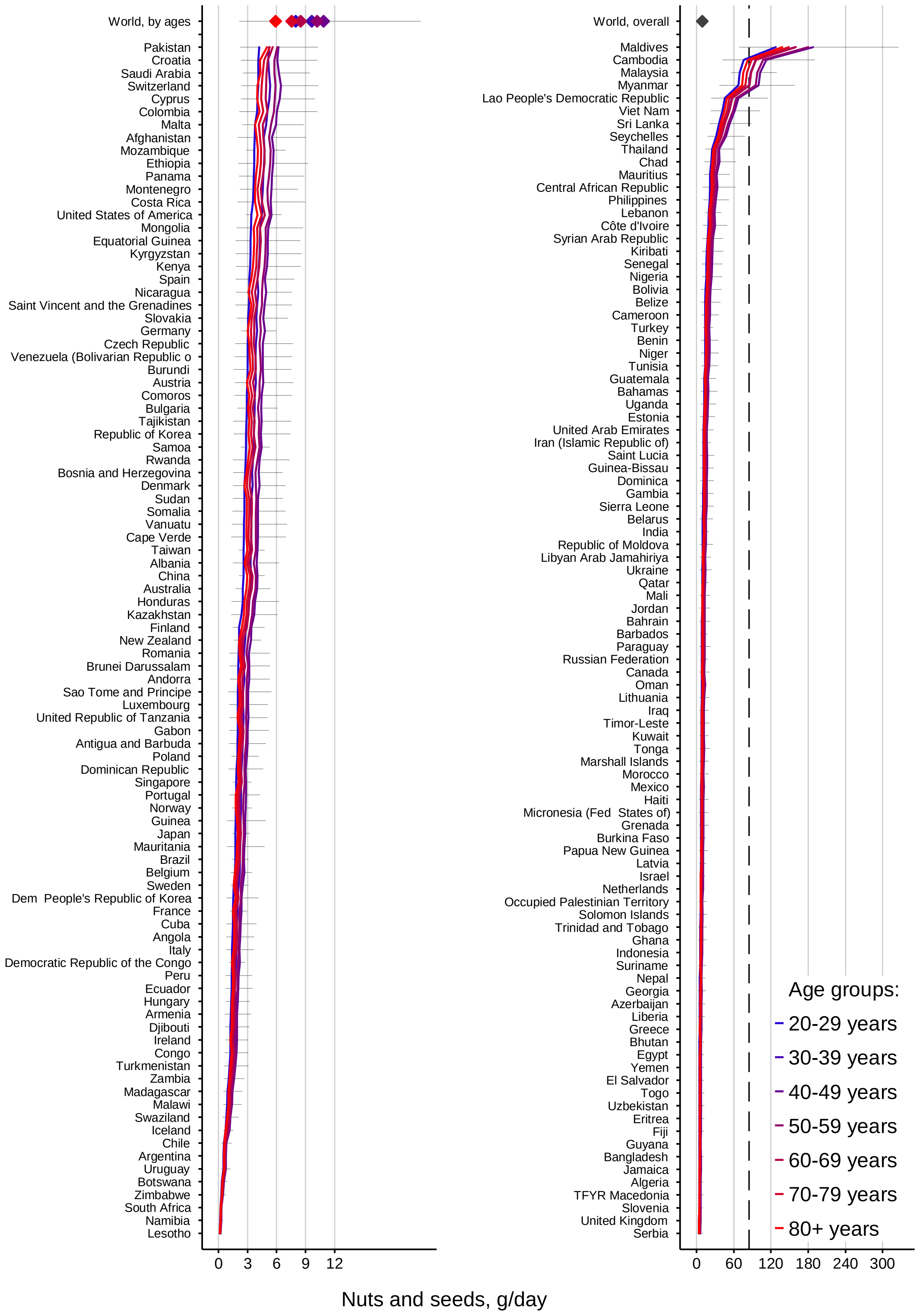

Consumption of beans and legumes among men

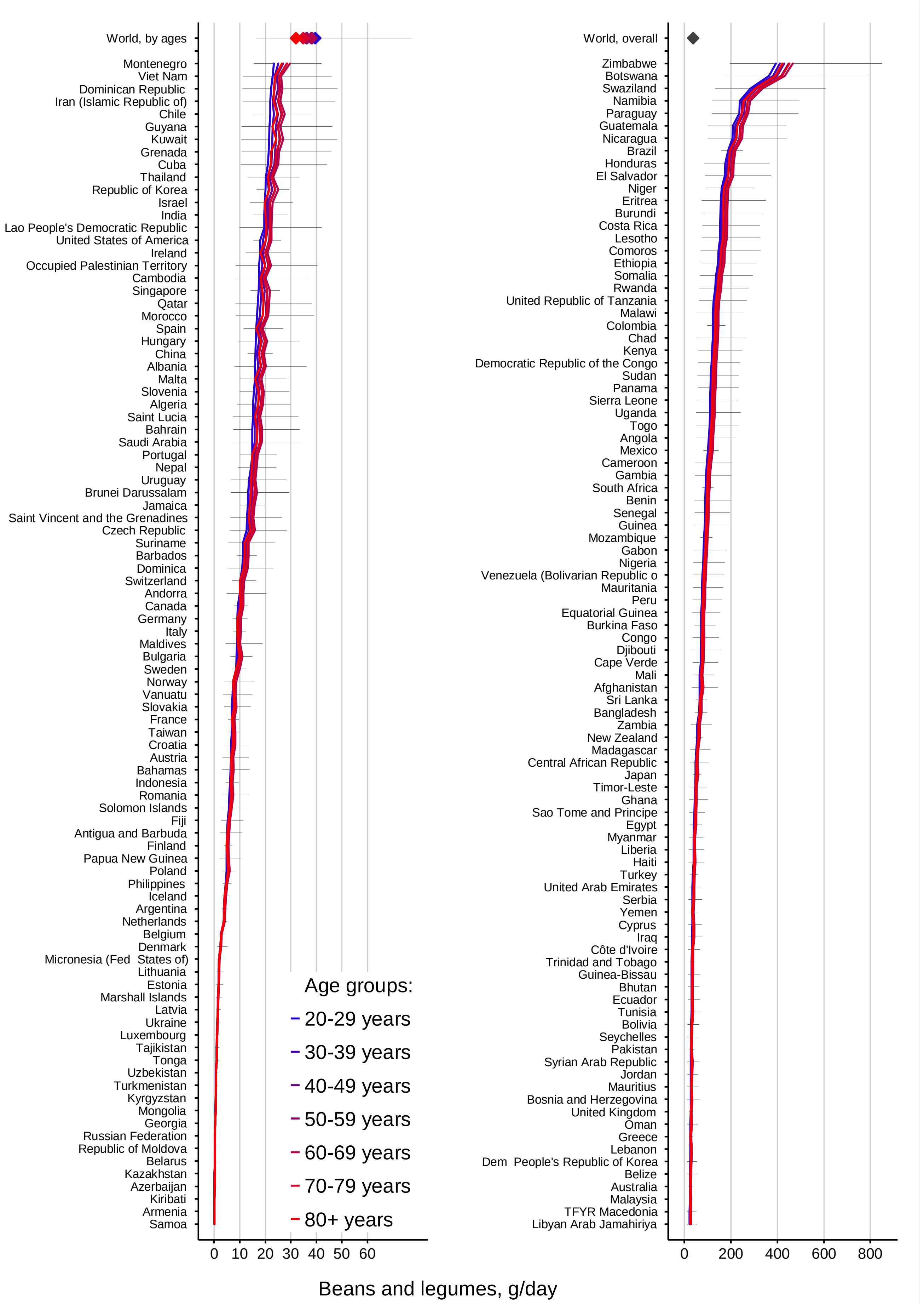

Figure S7. Consumption of beans and legumes among men and women aged 20 years or older in 187 countries. Countries are ordered by the mean consumption levels among men and women with 20-29 years of age, from the lowest at the bottom-left to the highest at the top-right. Error bars represent a lower side of 95% uncertainty interval (UI) for the lowest estimate and an upper side of 95% UI for the highest estimate in each country. The dashed vertical line represents mean of the theoretical minimal risk exposure distribution for consumption of beans and legumes.

Consumption of beans and legumes among women

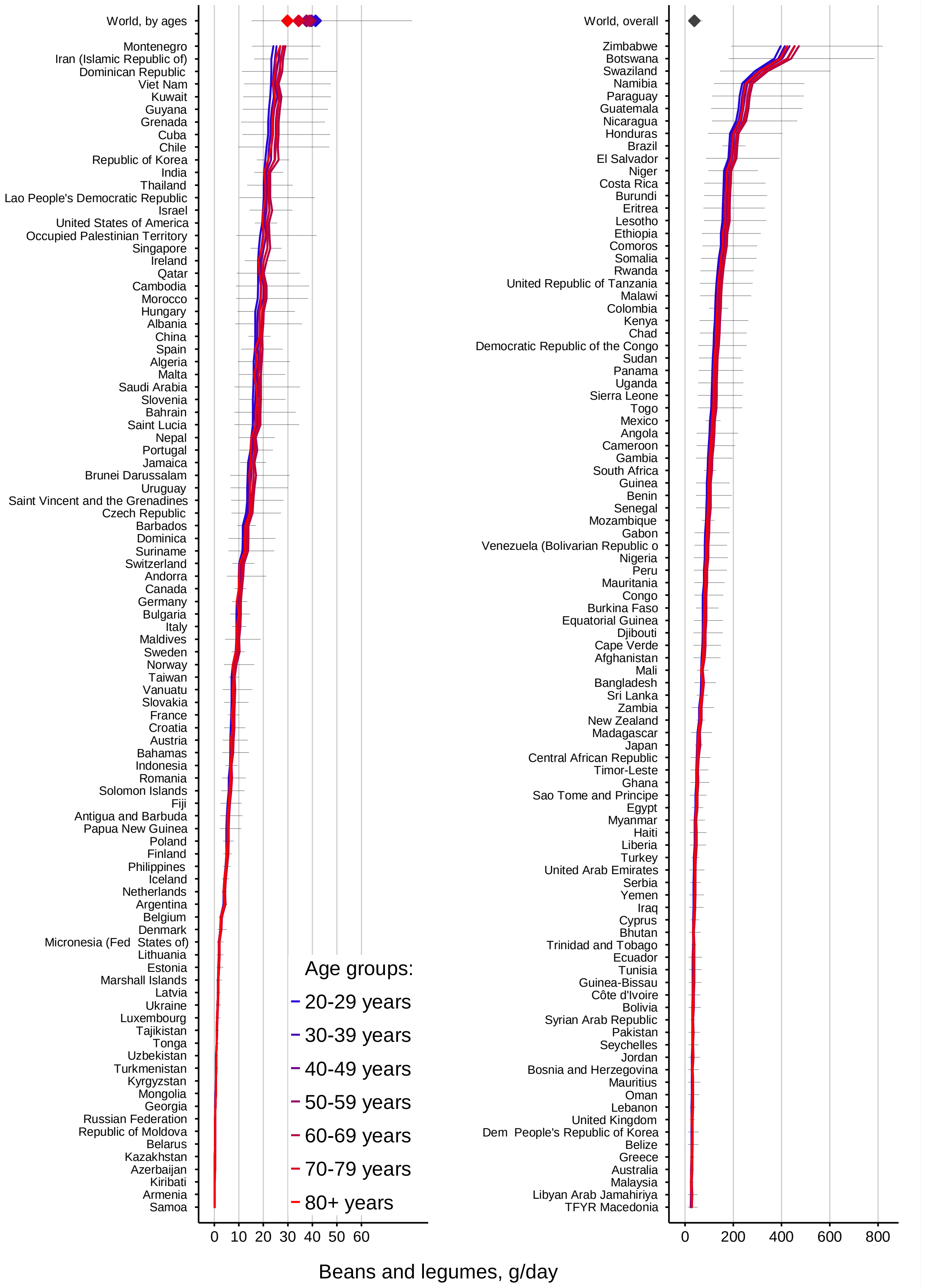

Milk consumption among men

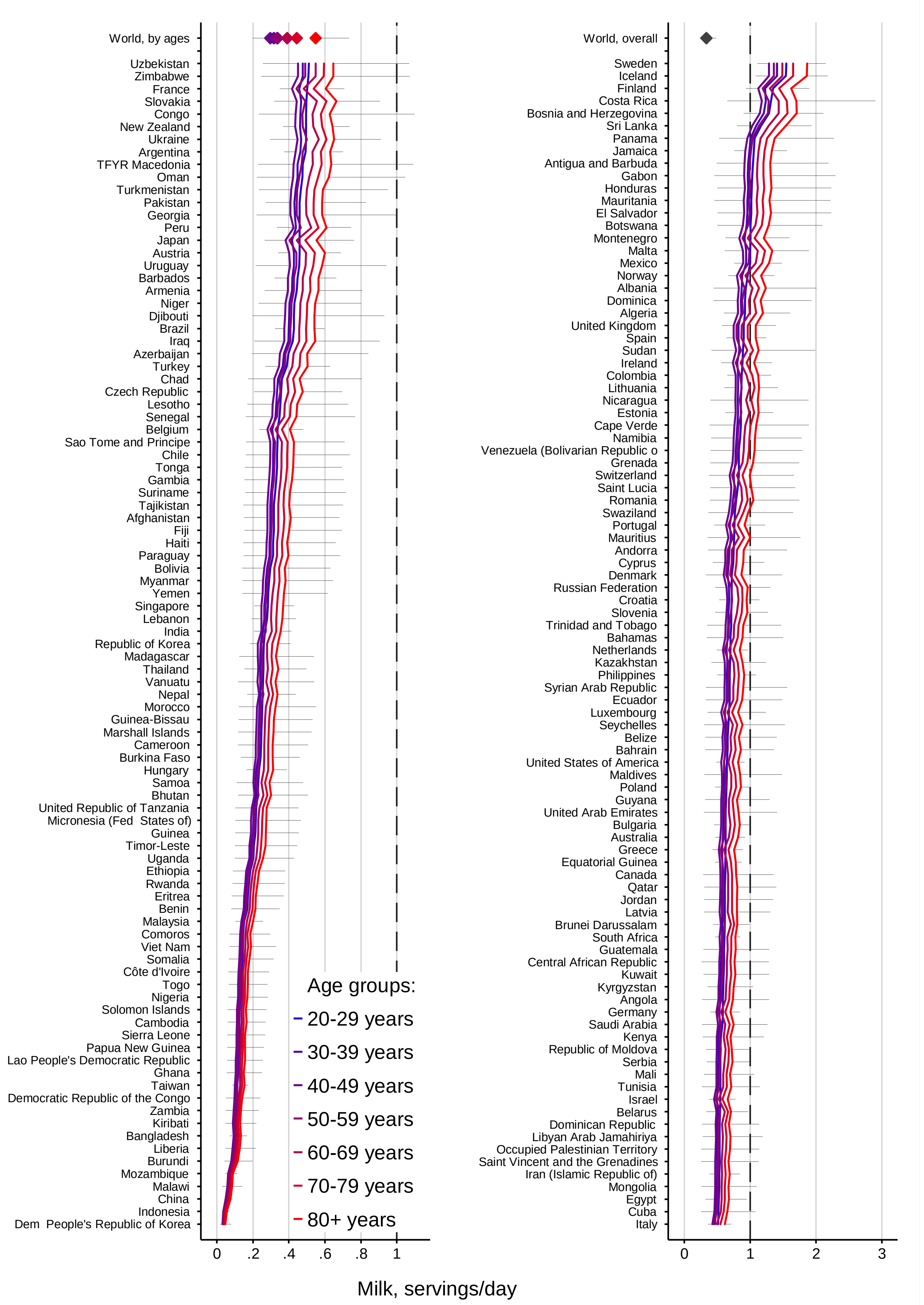

Figure S8. Milk consumption among men and women aged 20 years or older in 187 countries. The unit is 1 serving (=8 oz or 226.8 ml) per day. Countries are ordered by the mean consumption levels among men and women with 20-29 years of age, from the lowest at the bottom-left to the highest at the top-right. Error bars represent a lower side of 95% uncertainty interval (UI) for the lowest estimate and an upper side of 95% UI for the highest estimate. The dashed vertical line represents mean of the theoretical minimal risk exposure distribution for milk consumption.

Milk consumption among women

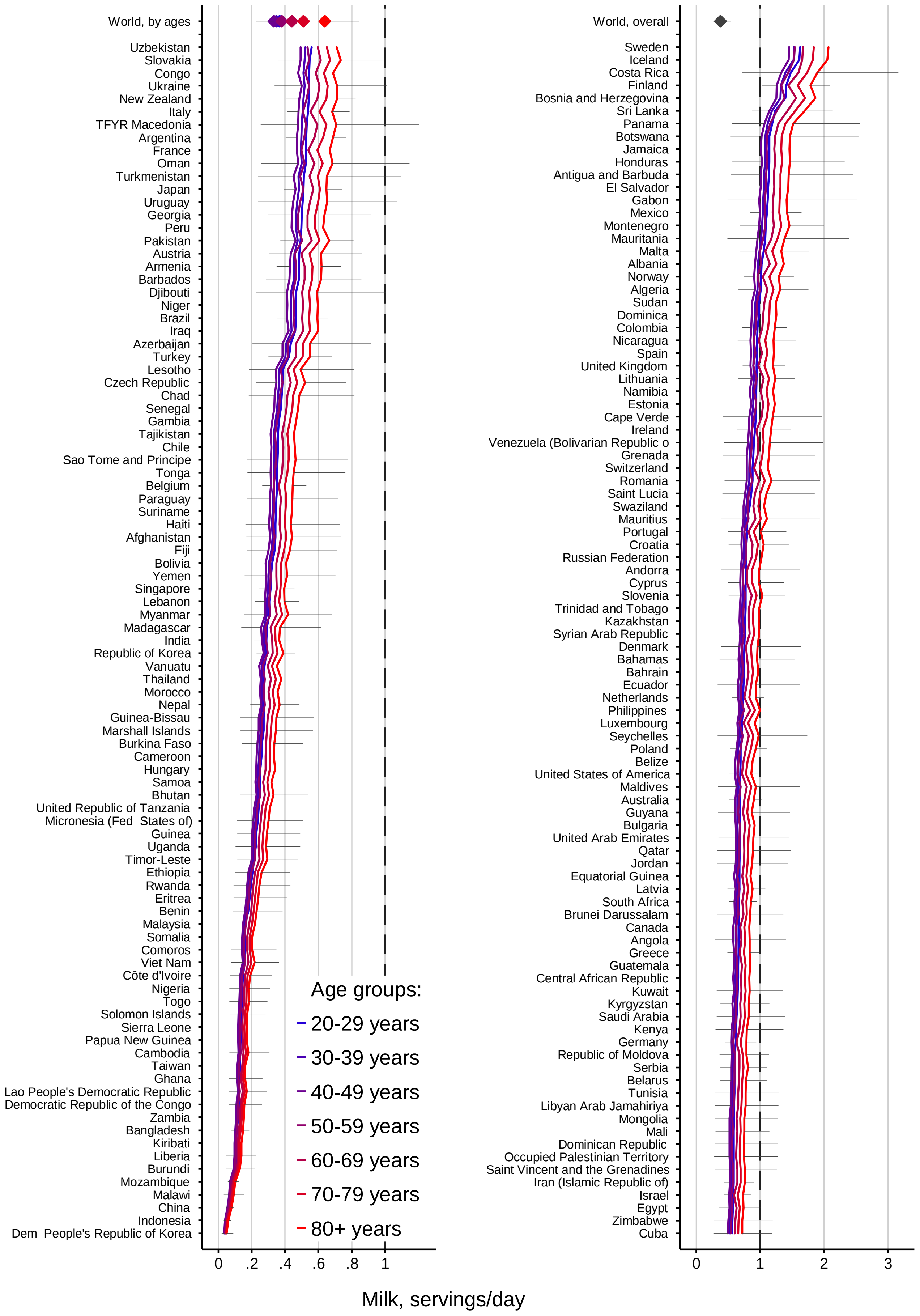

Dietary Fibre consumption among men

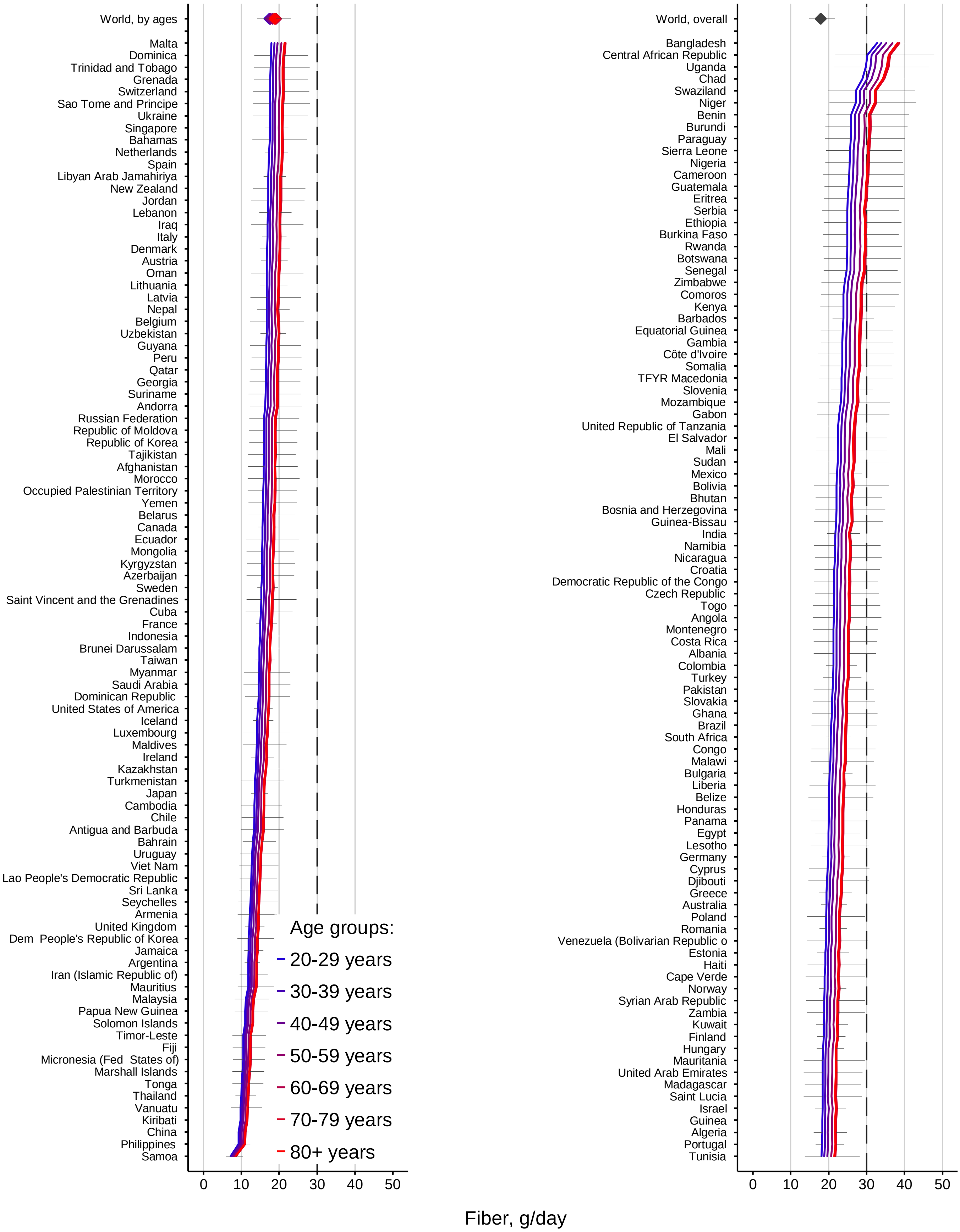

Figure S9. Dietary fibre consumption among men and women aged 20 years or older in 187 countries.
Countries are ordered by the mean consumption levels among men and women with 20-29 years of age, from the lowest at the bottom-left to the highest at the top-right. Error bars for each country represent a lower side of 95% uncertainty interval (UI) for the lowest estimate and an upper side of 95% UI for the highest estimate. The dashed vertical line represents mean of the theoretical minimal risk exposure distribution for dietary fibre consumption.

Dietary fibre consumption among women

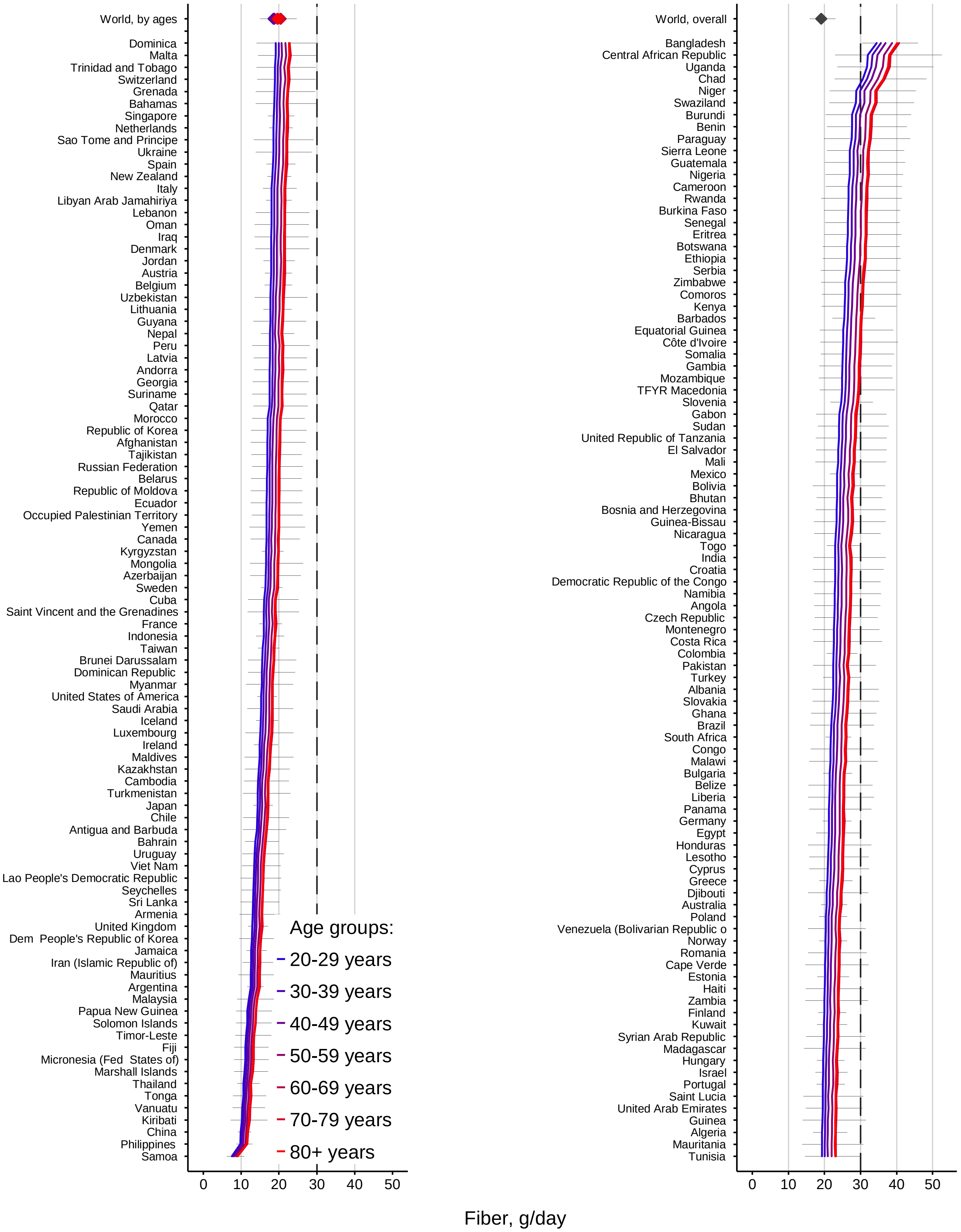

Polyunsaturated fat consumption among men

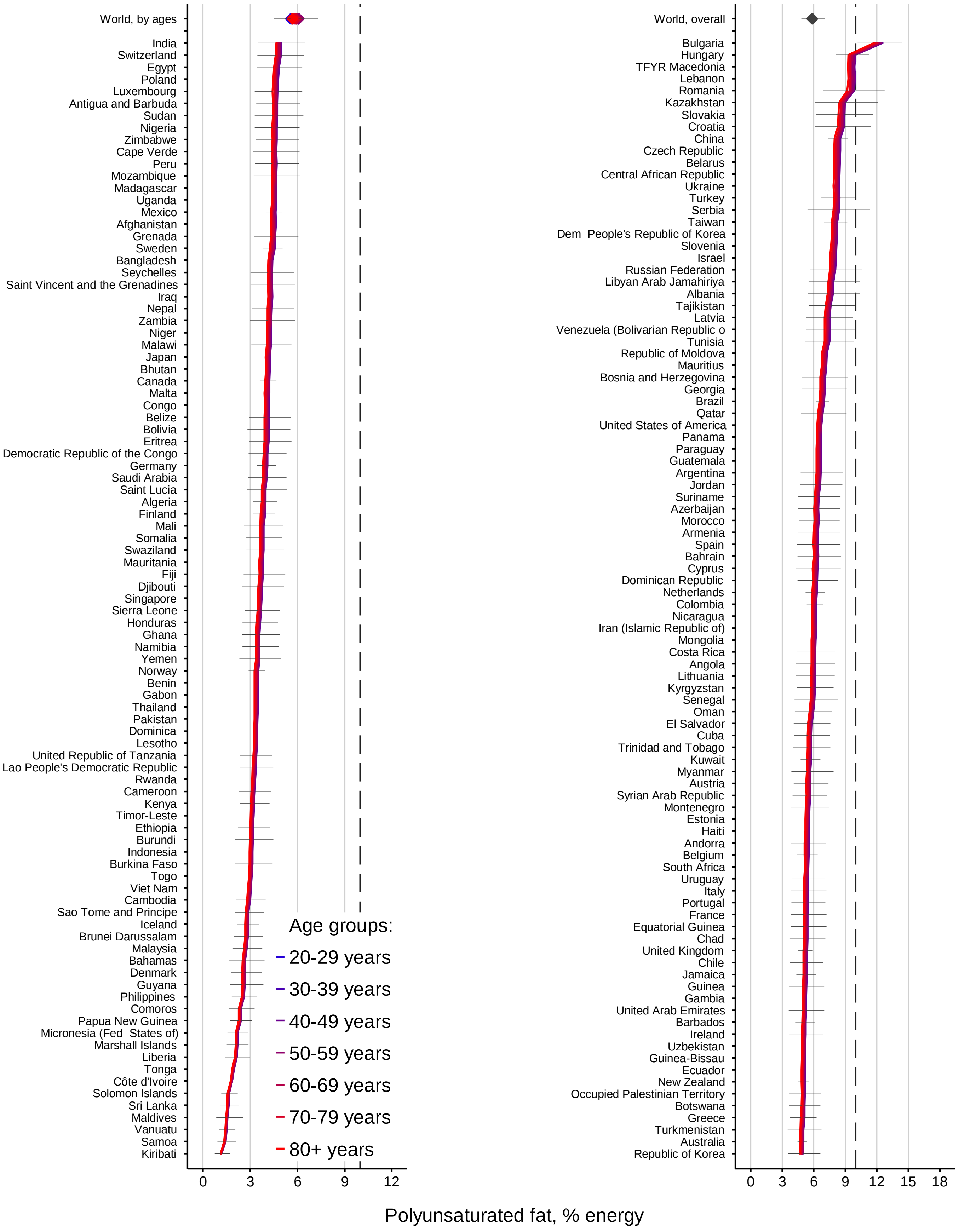

Figure S10. Polyunsaturated fat consumption among men and women aged 20 years or older in 187 countries. Countries are ordered by the mean consumption levels among men and women with 20-29 years of age, from the lowest at the bottom-left to the highest at the top-right. Error bars for each country represent a lower side of 95% uncertainty interval (UI) for the lowest estimate and an upper side of 95% UI for the highest estimate. The dashed vertical line represents mean of the theoretical minimal risk exposure distribution for polyunsaturated fat consumption.

Polyunsaturated fat consumption among women

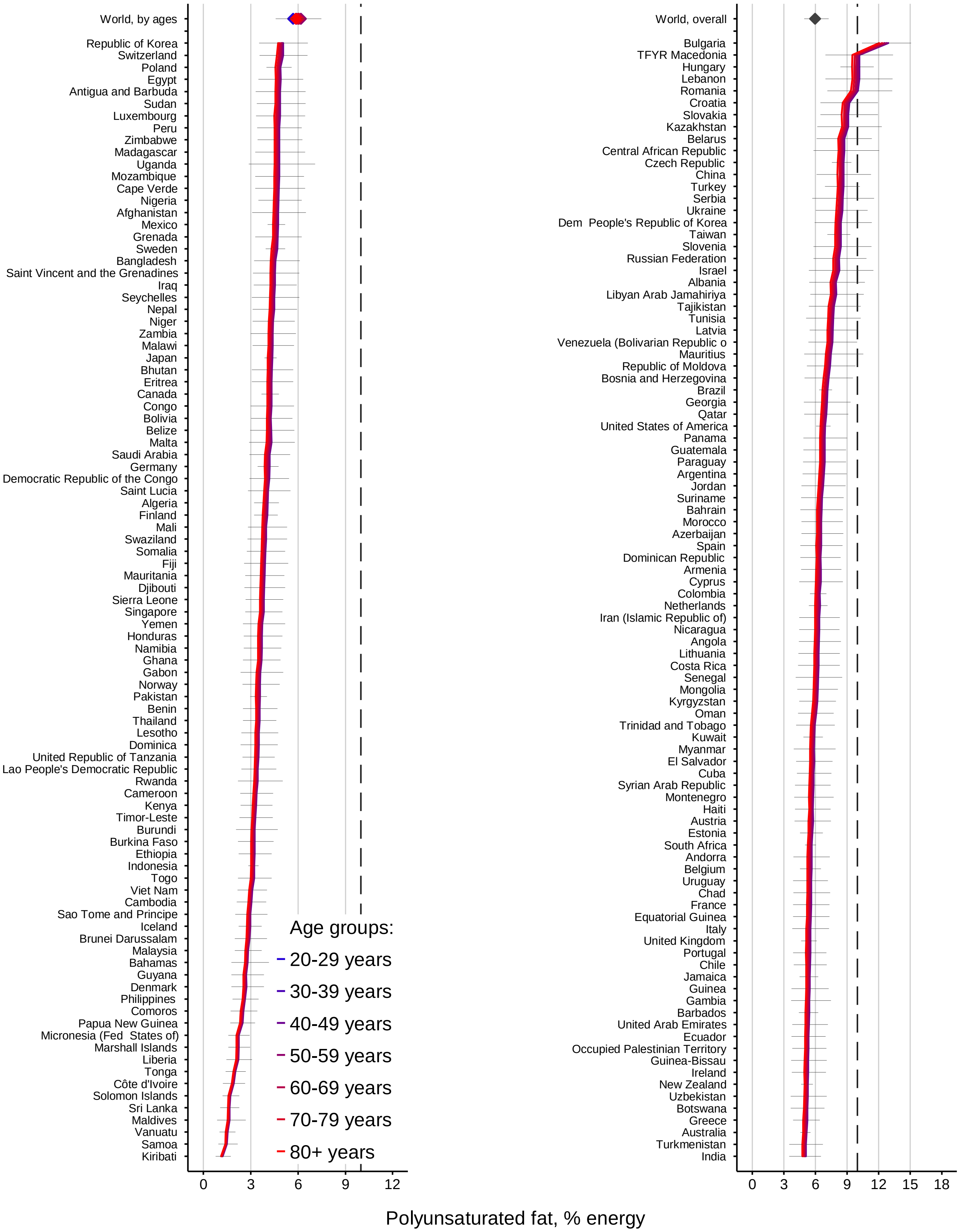

Consumption of seafood omega-3 fatty acids among men

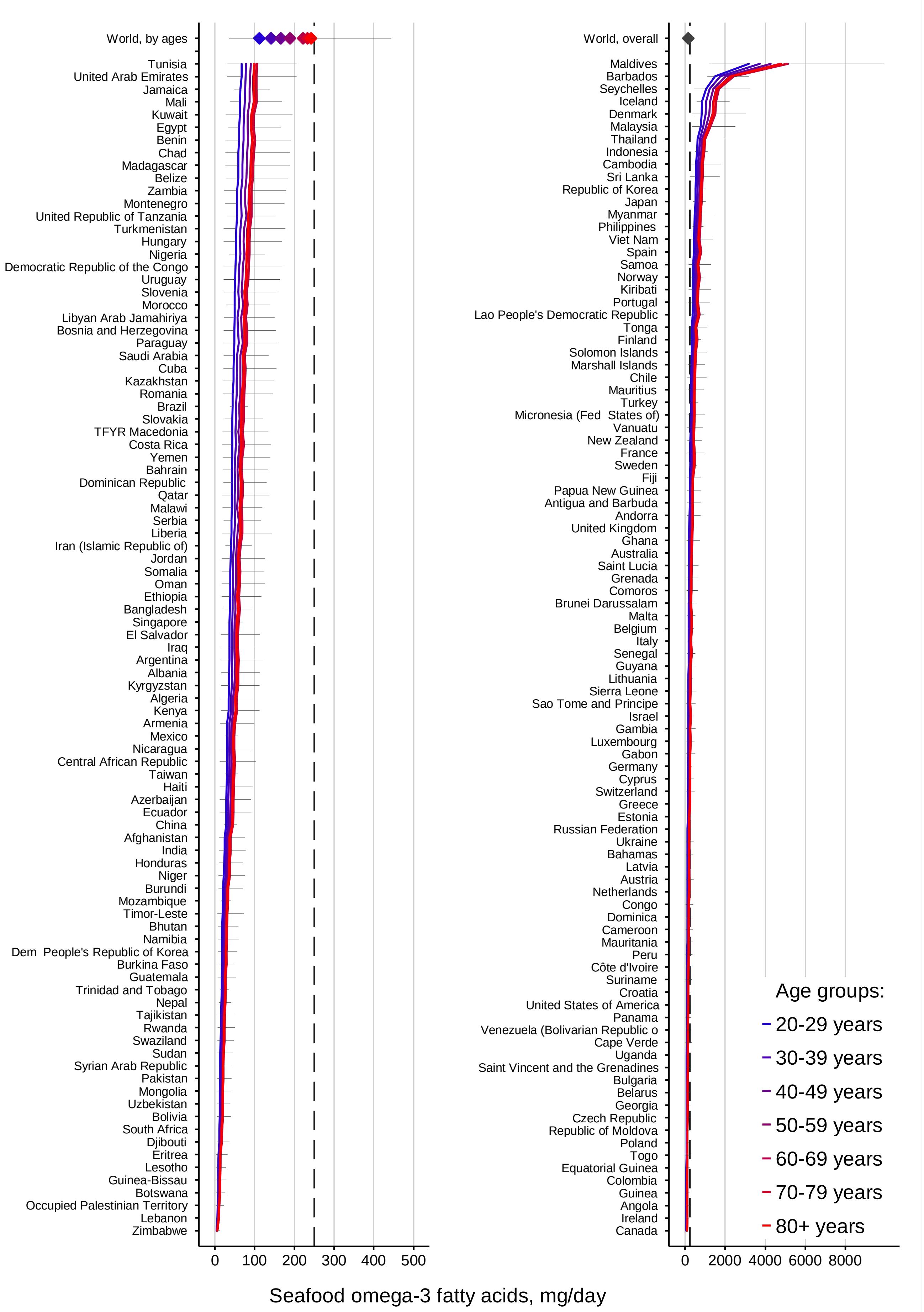

Figure S11. Consumption of seafood omega-3 fatty acids among men and women aged 20 years or older in 187 countries. Countries are ordered by levels among men and women with 20-29 years of age, from the lowest at the bottom-left to the highest at the top-right. Error bars represent a lower side of 95% uncertainty interval (UI) for the lowest estimate and an upper side of 95% UI for the highest estimate in each country. The dashed vertical line represents mean of the theoretical minimal risk exposure distribution for consumption of seafood omega-3 fatty acids.

Consumption of seafood omega-3 fatty acids among women

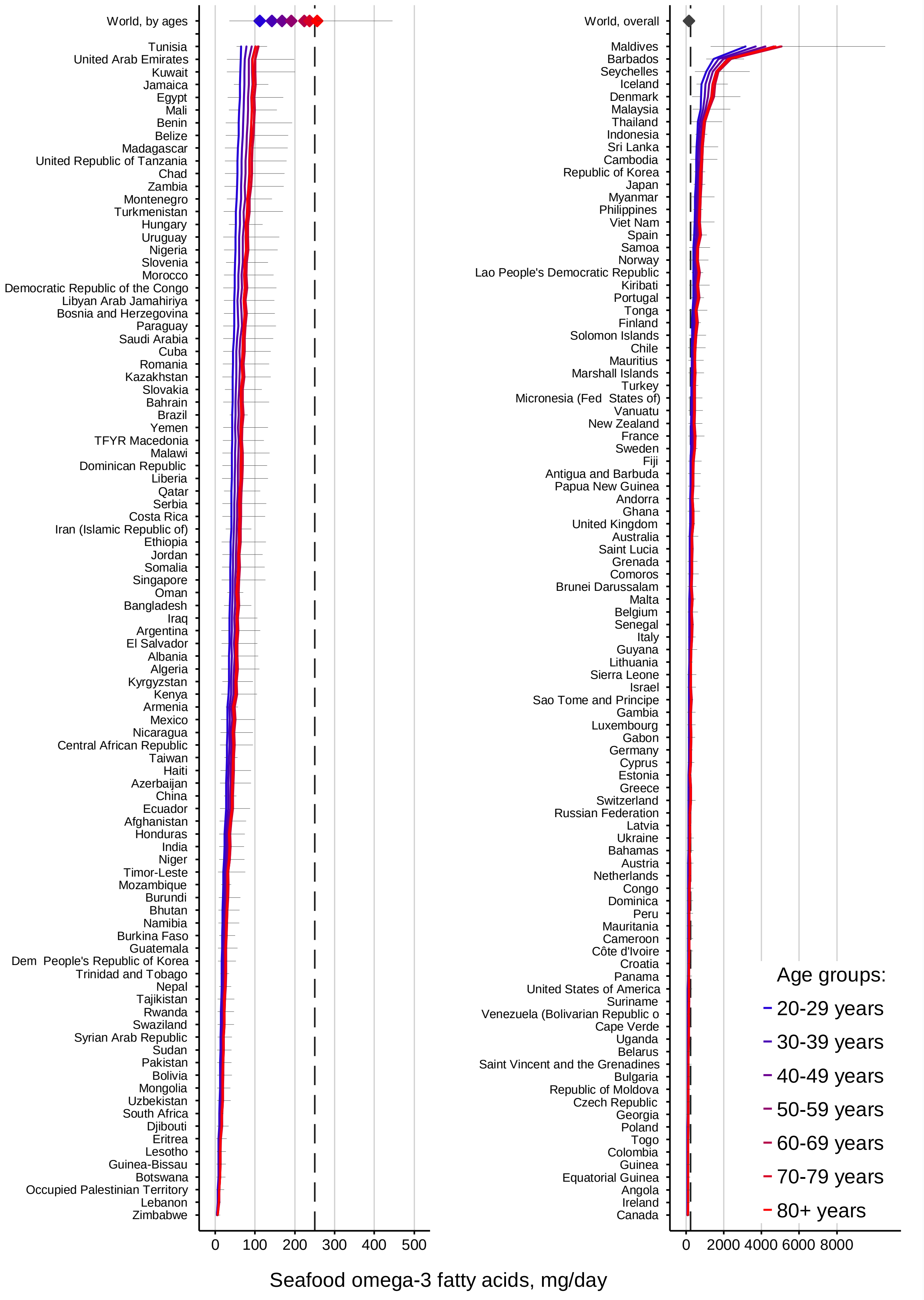

Consumption of plant omega-3 fatty acids among men

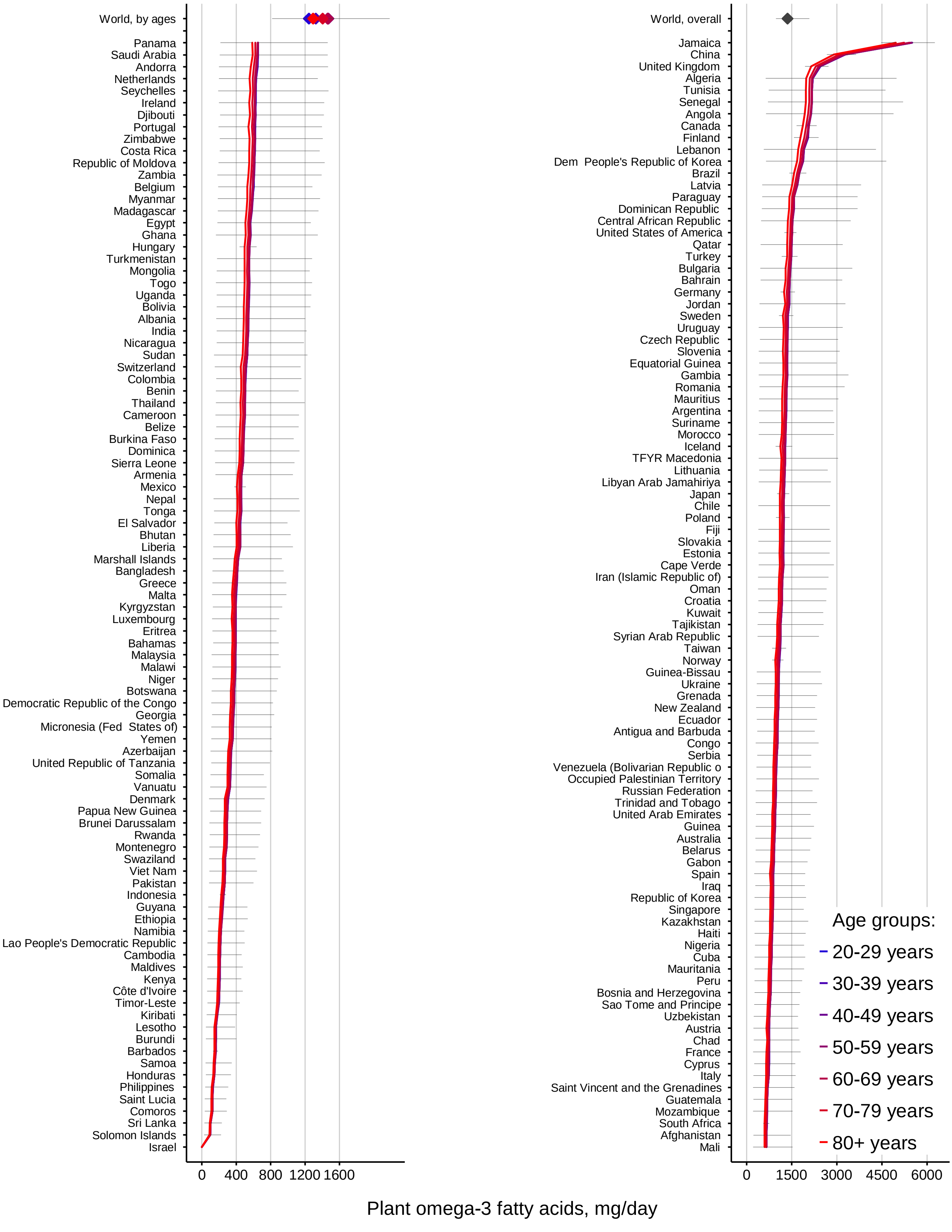

Figure S12. Consumption of plant omega-3 fatty acids among men and women aged 20 years or older in 187 countries. Countries are ordered by the mean consumption levels among men and women with 20-29 years of age, from the lowest at the bottom-left to the highest at the top-right. Error bars for each country represent a lower side of 95% uncertainty interval (UI) for the lowest estimate and an upper side of 95% UI for the highest estimate. The dashed vertical line represents mean of the theoretical minimal risk exposure distribution for consumption of plant omega-3 fatty acids.

Consumption of plant omega-3 fatty acids among women

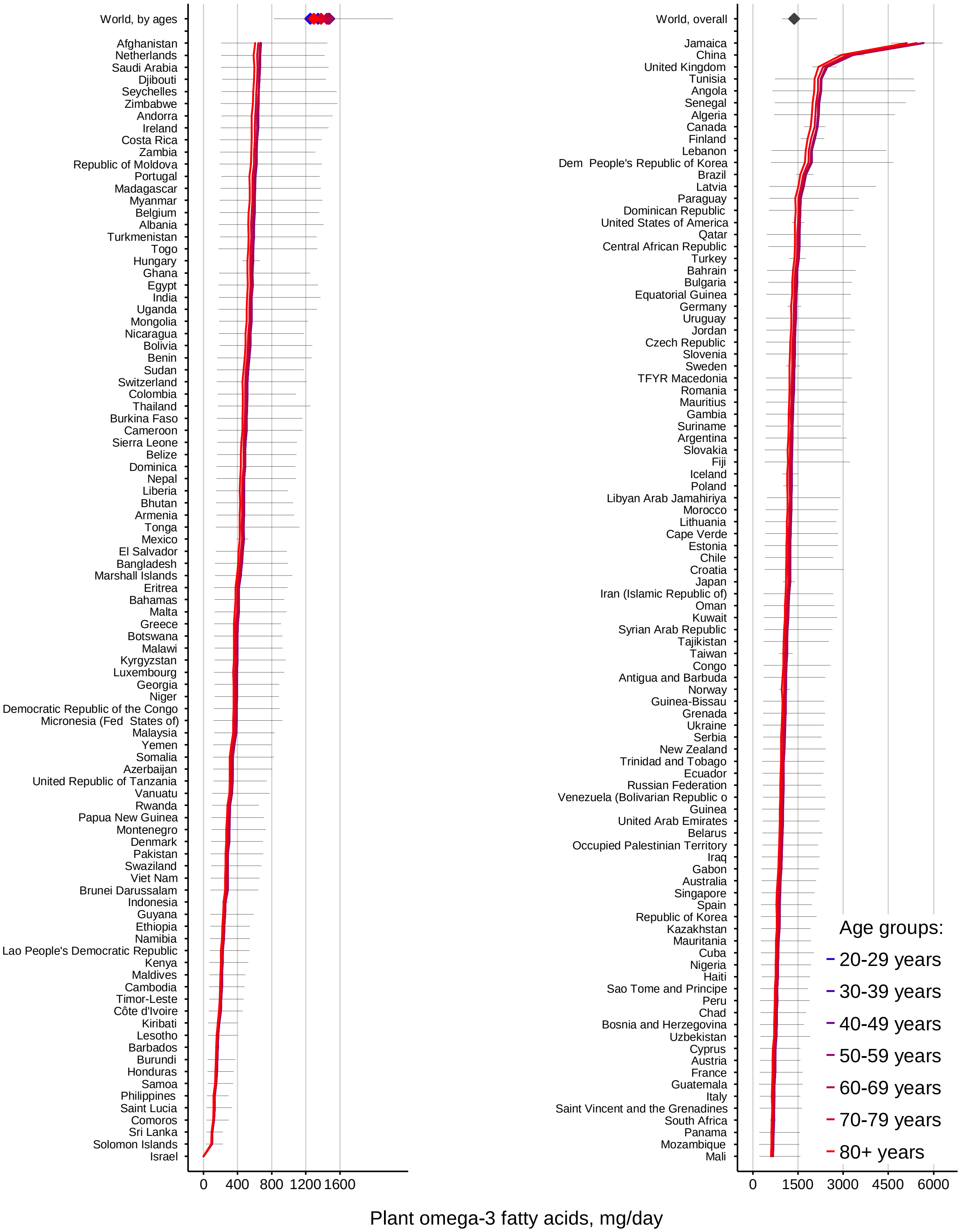

Calcium consumption among men

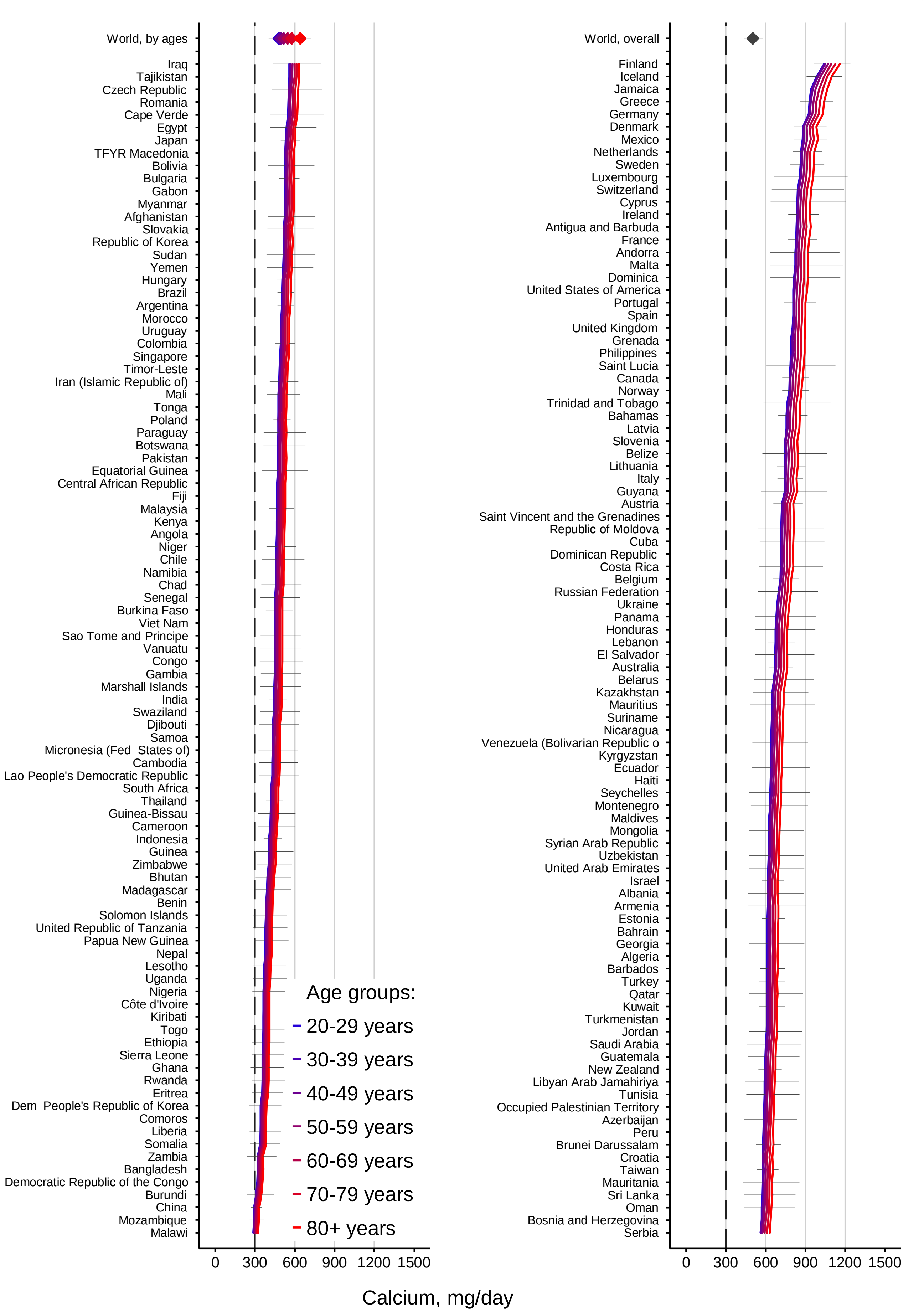

Figure S13. Calcium consumption among men and women aged 20 years or older in 187 countries.
Countries are ordered by the mean consumption levels among men and women with 20-29 years of age, from the lowest at the bottom-left to the highest at the top-right. Error bars for each country represent a lower side of 95% uncertainty interval (UI) for the lowest estimate and an upper side of 95% UI for the highest estimate. The dashed vertical line represents mean of the theoretical minimal risk exposure distribution for calcium consumption.

Calcium consumption among women

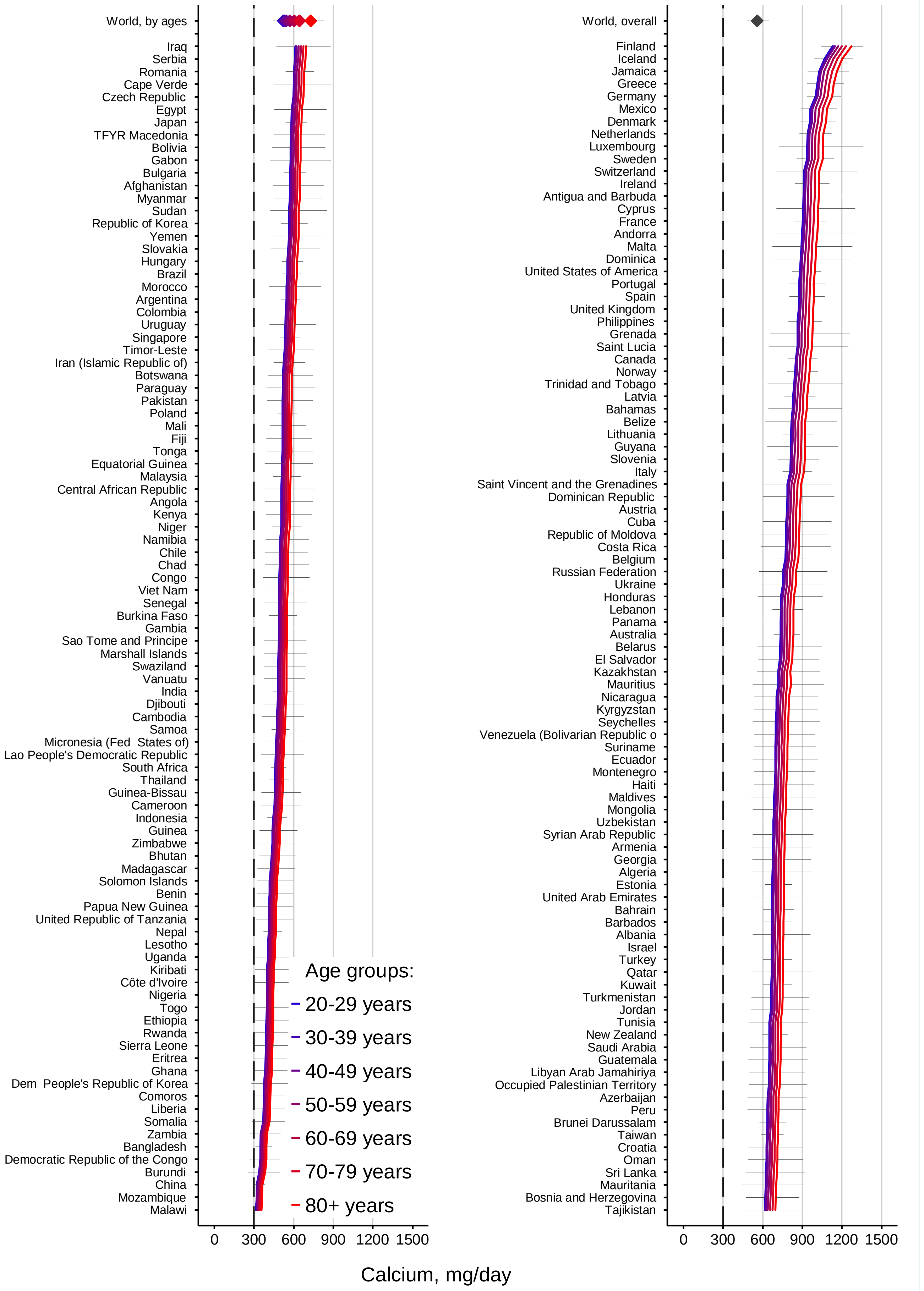

Sugar sweetened beverage consumption among men

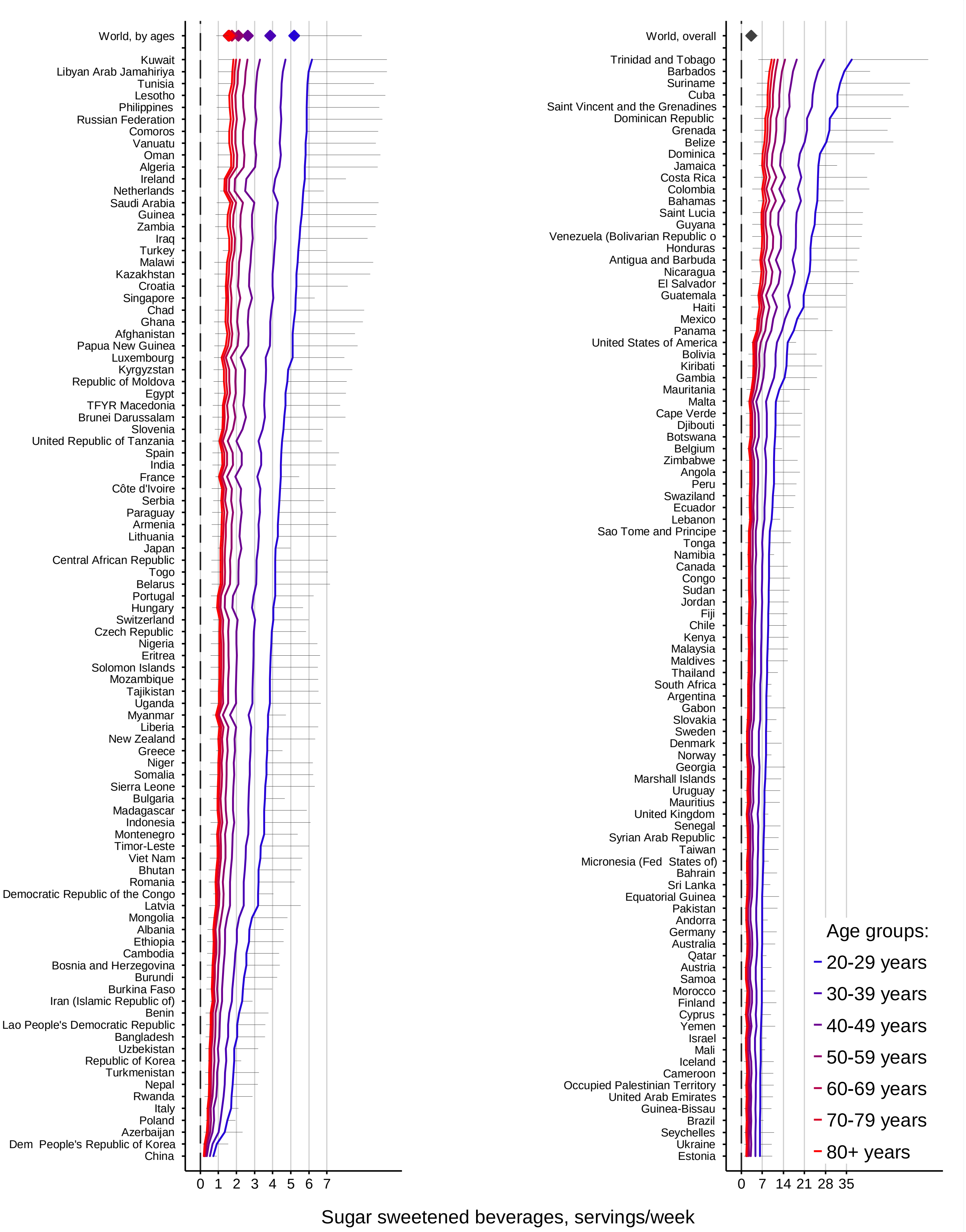

Figure S14. Sugar sweetened beverage consumption among men and women aged 20 years or older in 187 countries. The unit is 1 serving (=8 oz or 226.8 ml) per week. Countries are ordered by the mean consumption levels among men and women with 20-29 years of age, from the lowest at the bottom-left to the highest at the top-right. Error bars for each country represent a lower side of 95% uncertainty interval (UI) for the lowest estimate and an upper side of 95% UI for the highest estimate. The dashed vertical line represents mean of the theoretical minimal risk exposure distribution for sugar sweetened beverage consumption.

Sugar sweetened beverage consumption among women

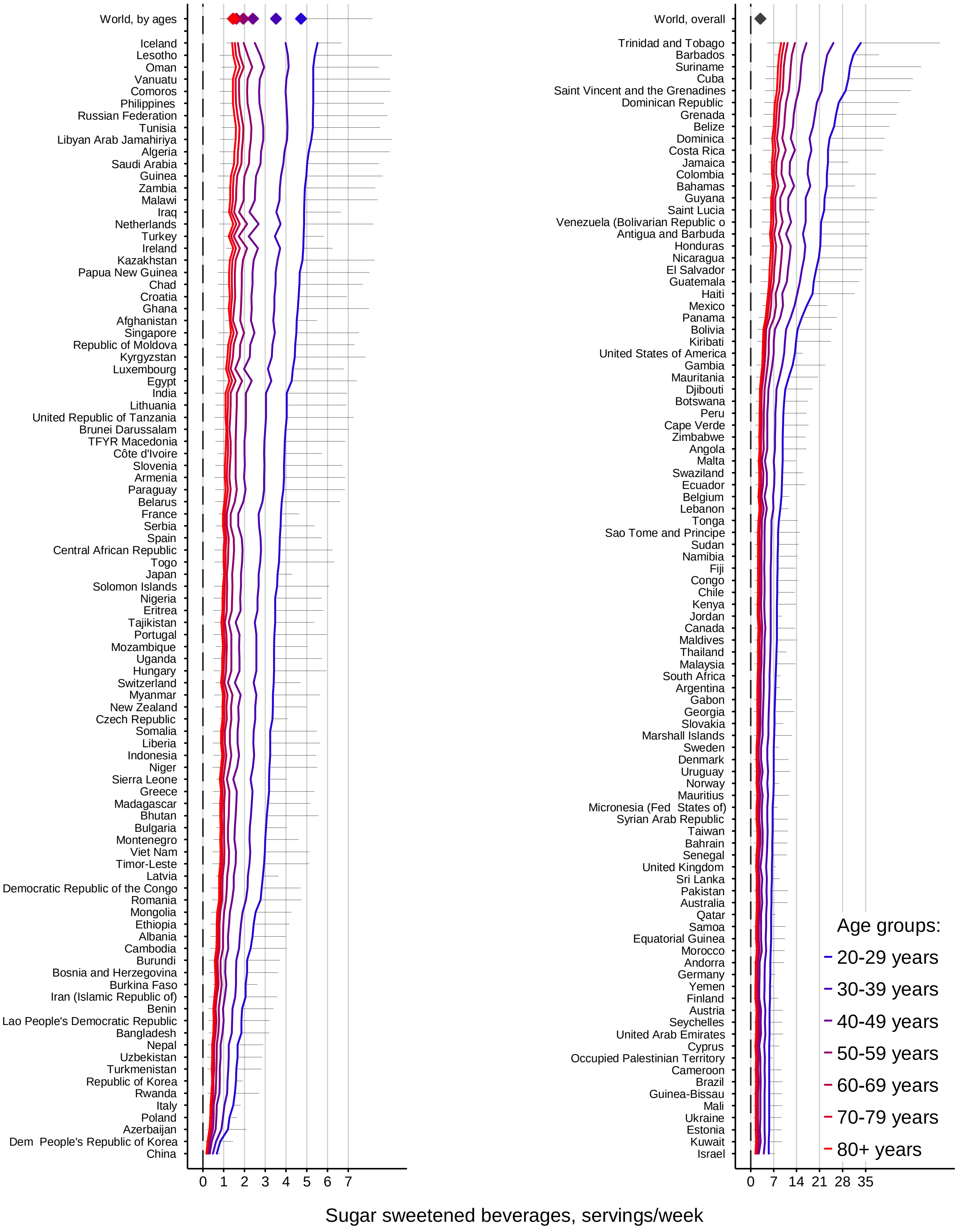

Unprocessed red meat consumption among men

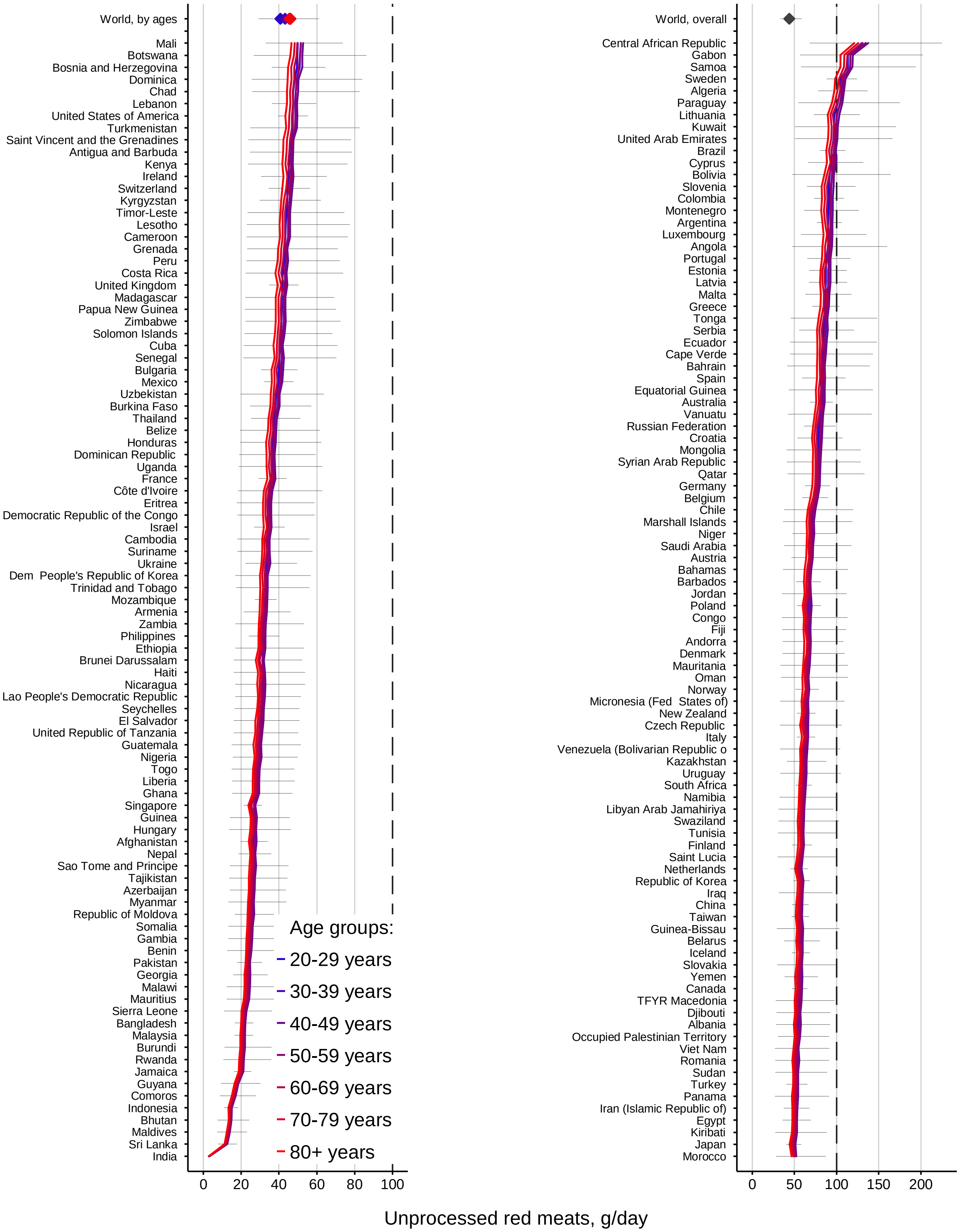

Figure S15. Unprocessed red meat consumption among men and women aged 20 years or older in 187 countries. Countries are ordered by the mean consumption levels among men and women with 20-29 years of age, from the lowest at the bottom-left to the highest at the top-right. Error bars for each country represent a lower side of 95% uncertainty interval (UI) for the lowest estimate and an upper side of 95% UI for the highest estimate. The dashed vertical line represents mean of the theoretical minimal risk exposure distribution for processed meat consumption.

Unprocessed red meat consumption among women

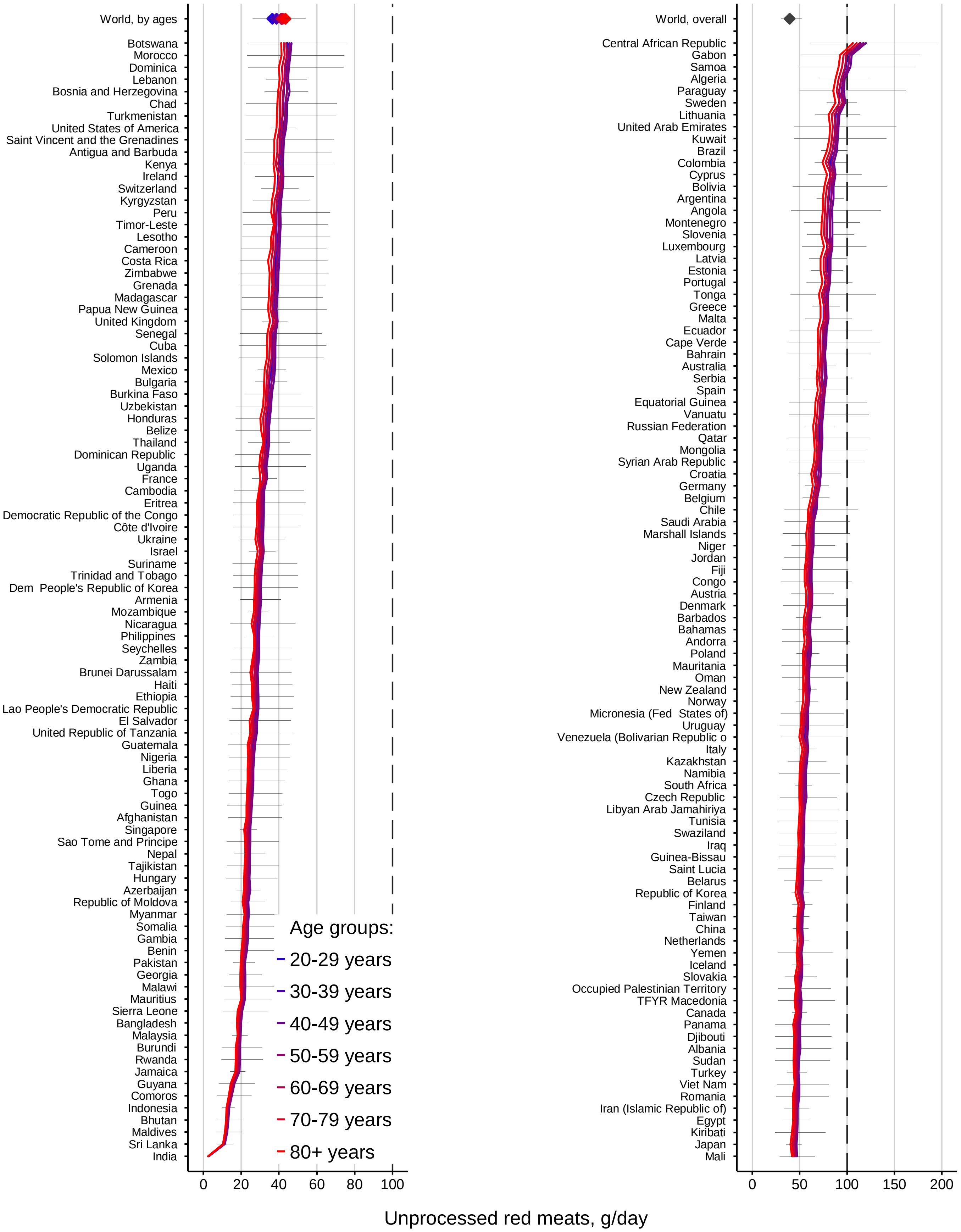

Processed meat consumption among men

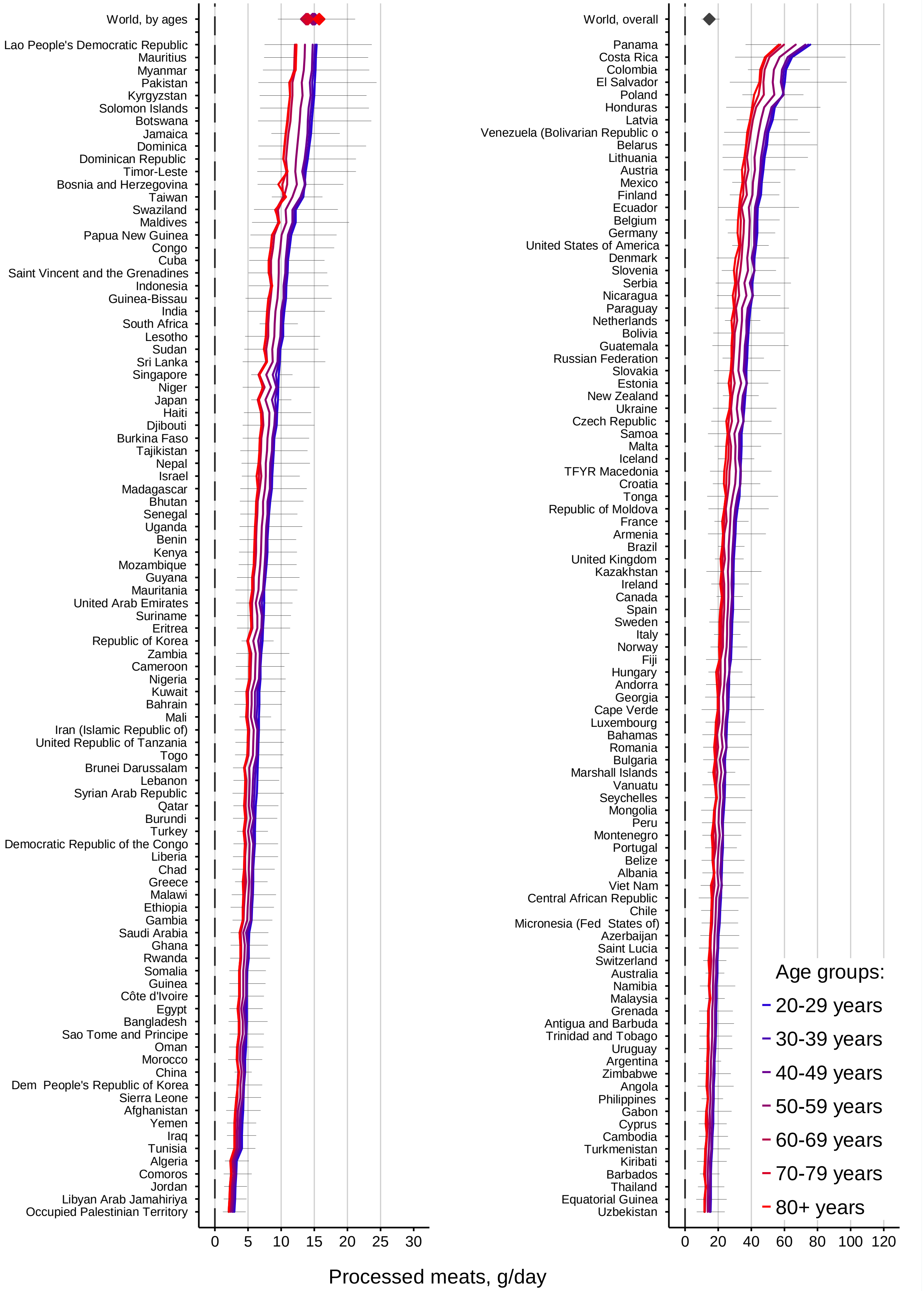

Figure S16. Processed meat consumption among men and women aged 20 years or older in 187 countries. Countries are ordered by the mean consumption levels among men and women with 20-29 years of age, from the lowest at the bottom-left to the highest at the top-right. Error bars represent a lower side of 95% uncertainty interval (UI) for the lowest estimate and an upper side of 95% UI for the highest estimate. The dashed vertical line represents mean of the theoretical minimal risk exposure distribution for unprocessed red meat consumption.

Processed meat consumption among women

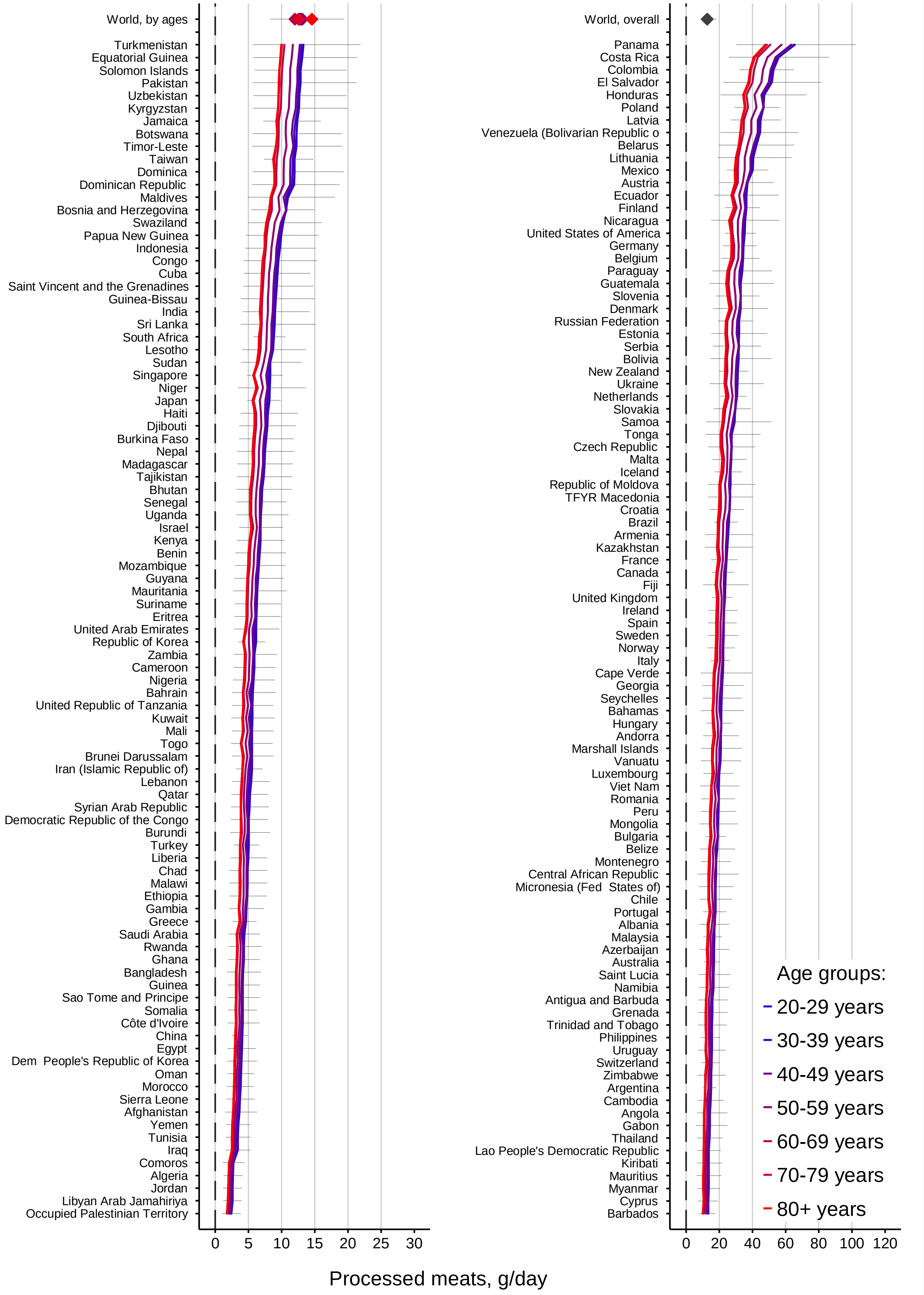

Saturated fat consumption among men

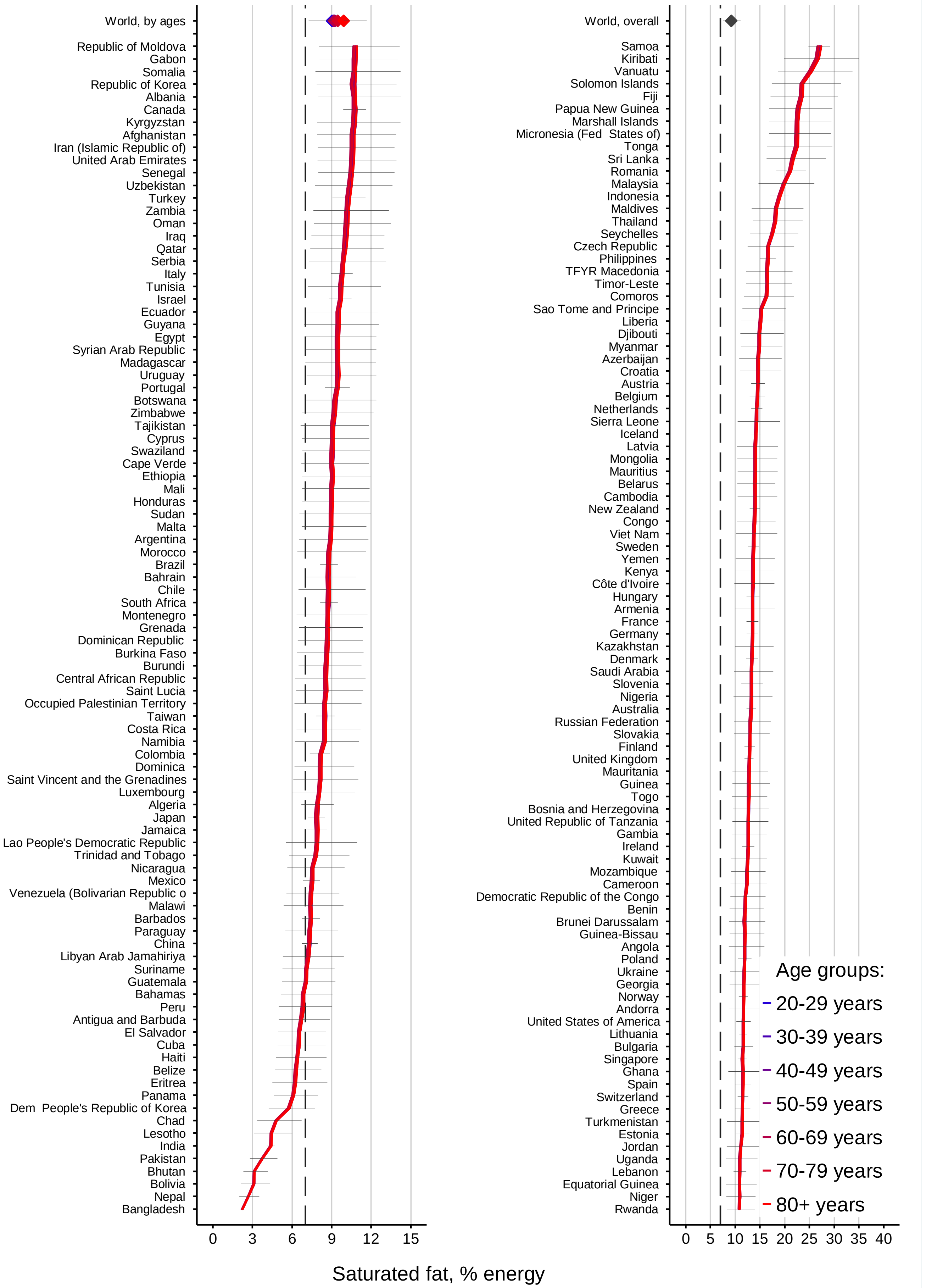

Figure S17. Saturated fat consumption among men and women aged 20 years or older in 187 countries.
Countries are ordered by the mean consumption levels among men and women with 20-29 years of age, from the lowest at the bottom-left to the highest at the top-right. Error bars for each country represent a lower side of 95% uncertainty interval (UI) for the lowest estimate and an upper side of 95% UI for the highest estimate. The dashed vertical line represents mean of the theoretical minimal risk exposure distribution for saturated fat consumption.

Saturated fat consumption among women

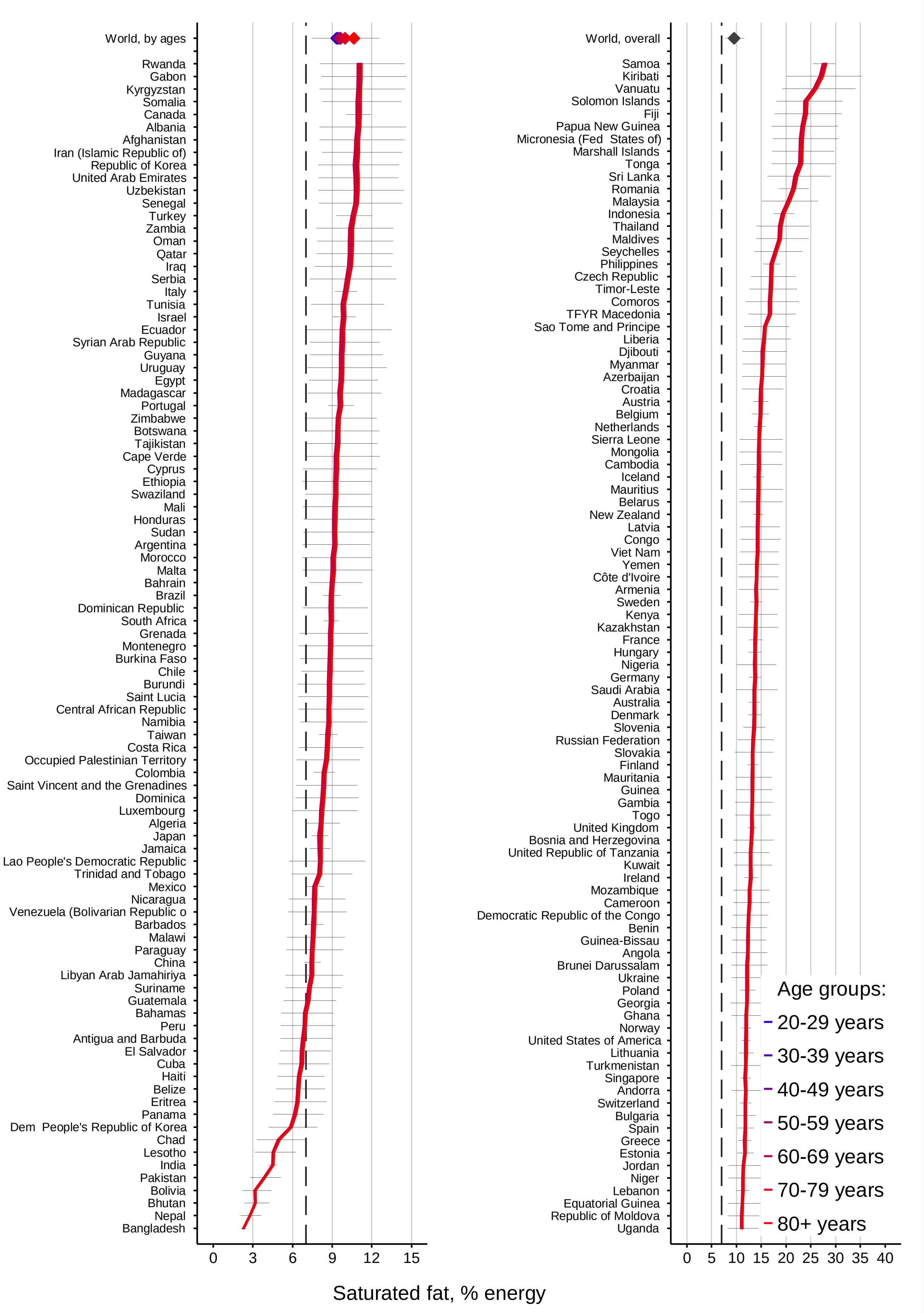

Consumption of trans fatty acids among men

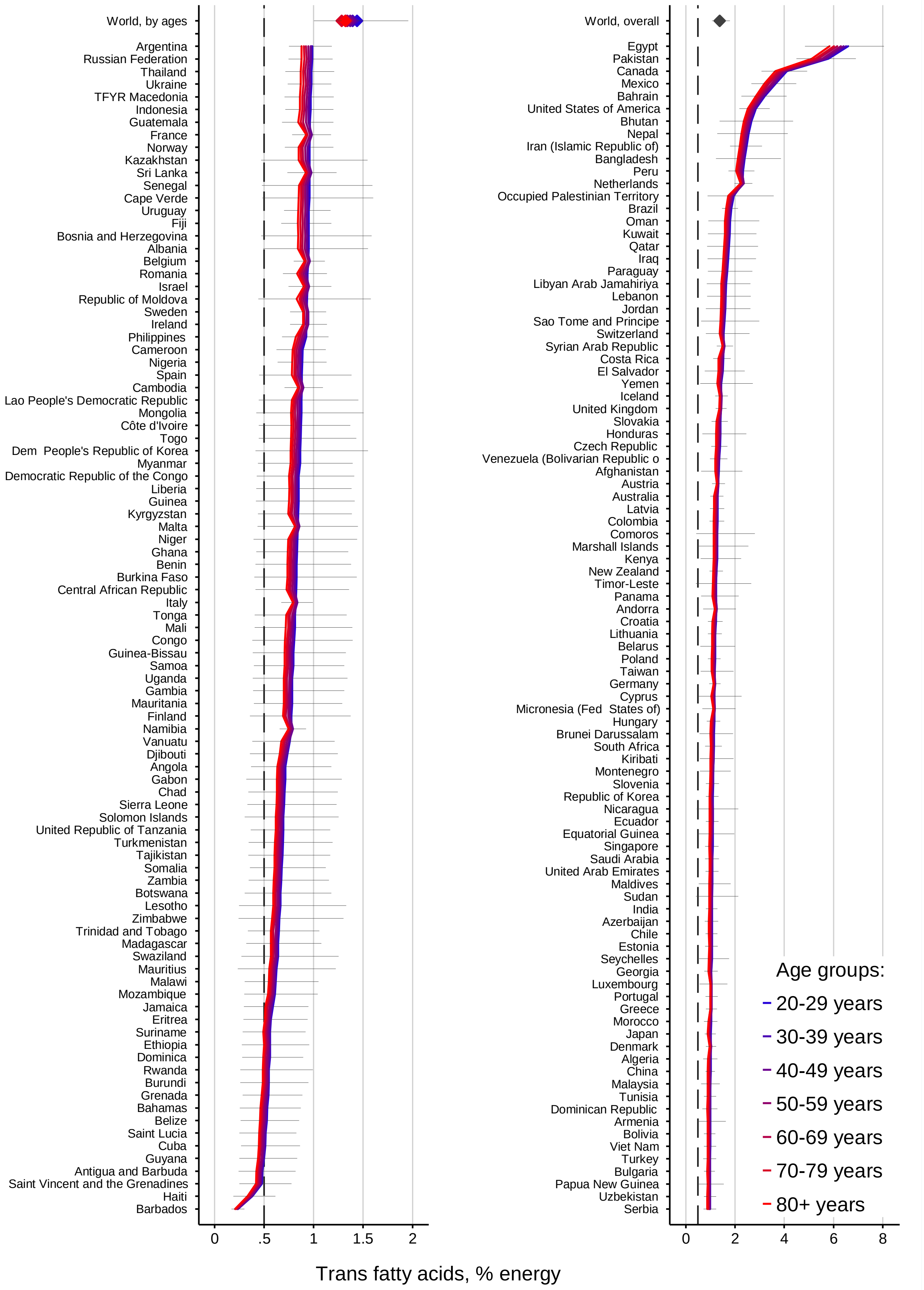

Figure S18. Consumption of trans fatty acids among men and women aged 20 years or older in 187 countries. Countries are ordered by the mean consumption levels among men and women with 20-29 years of age, from the lowest at the bottom-left to the highest at the top-right. Error bars for each country represent a lower side of 95% uncertainty interval (UI) for the lowest estimate and an upper side of 95% UI for the highest estimate. The dashed vertical line represents mean of the theoretical minimal risk exposure distribution for trans fatty acid consumption.

Consumption of trans fatty acids among women

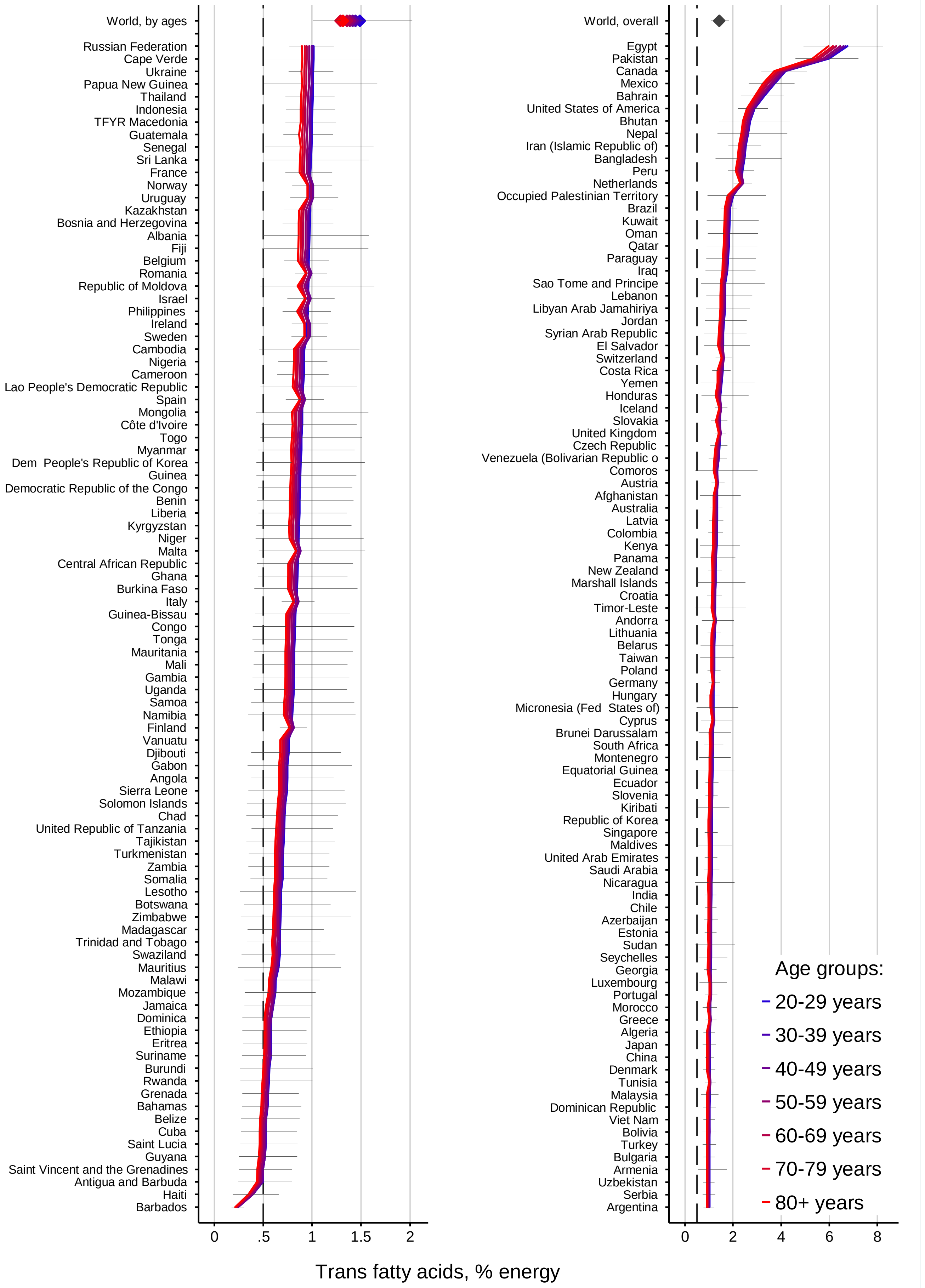

Dietary cholesterol consumption among men

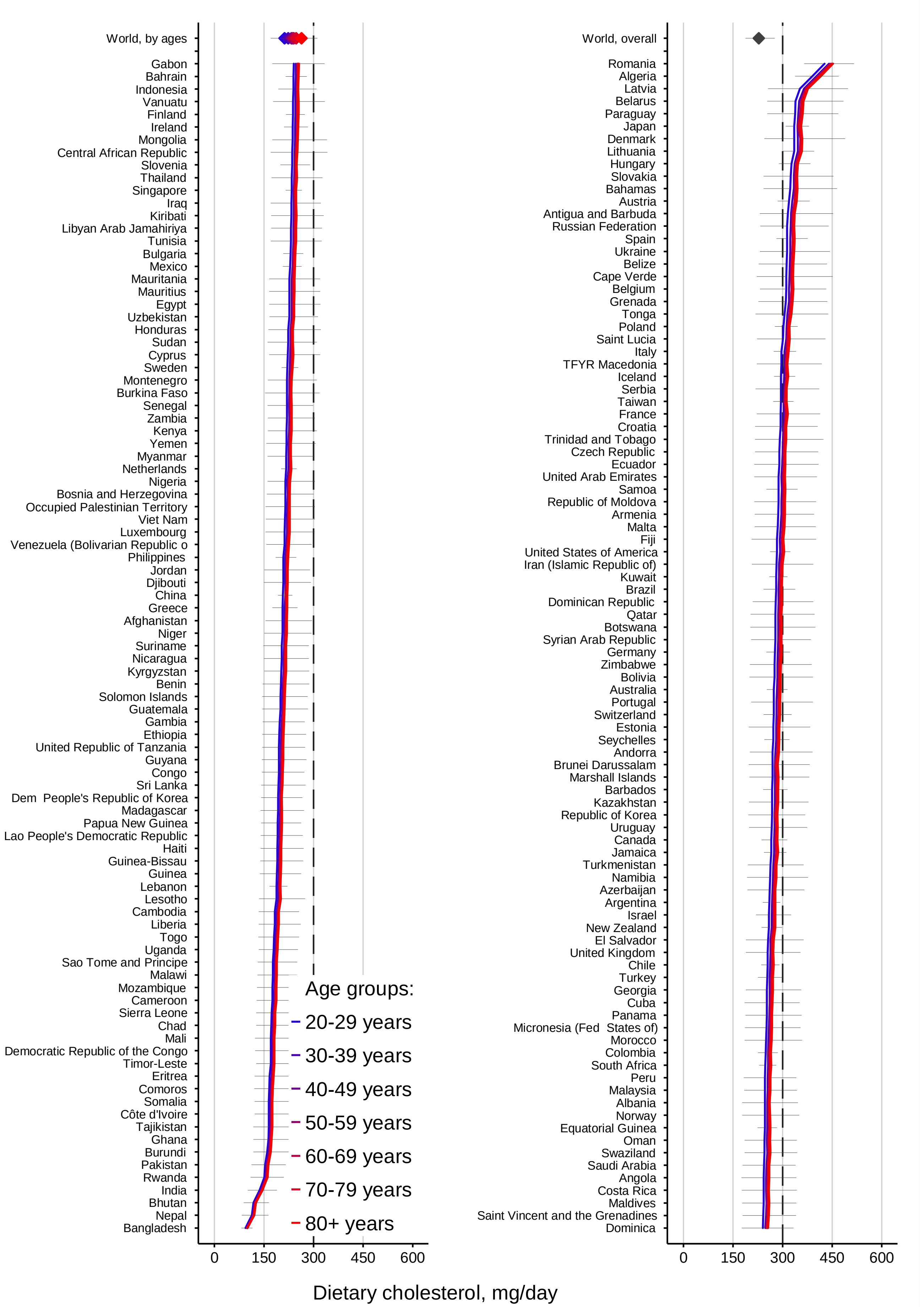

Figure S19. Dietary cholesterol consumption among men and women aged 20 years or older in 187 countries. Countries are ordered by the mean consumption levels among men and women with 20-29 years of age, from the lowest at the bottom-left to the highest at the top-right. Error bars for each country represent a lower side of 95% uncertainty interval (UI) for the lowest estimate and an upper side of 95% UI for the highest estimate. The dashed vertical line represents mean of the theoretical minimal risk exposure distribution for dietary cholesterol consumption.

Dietary cholesterol consumption among women

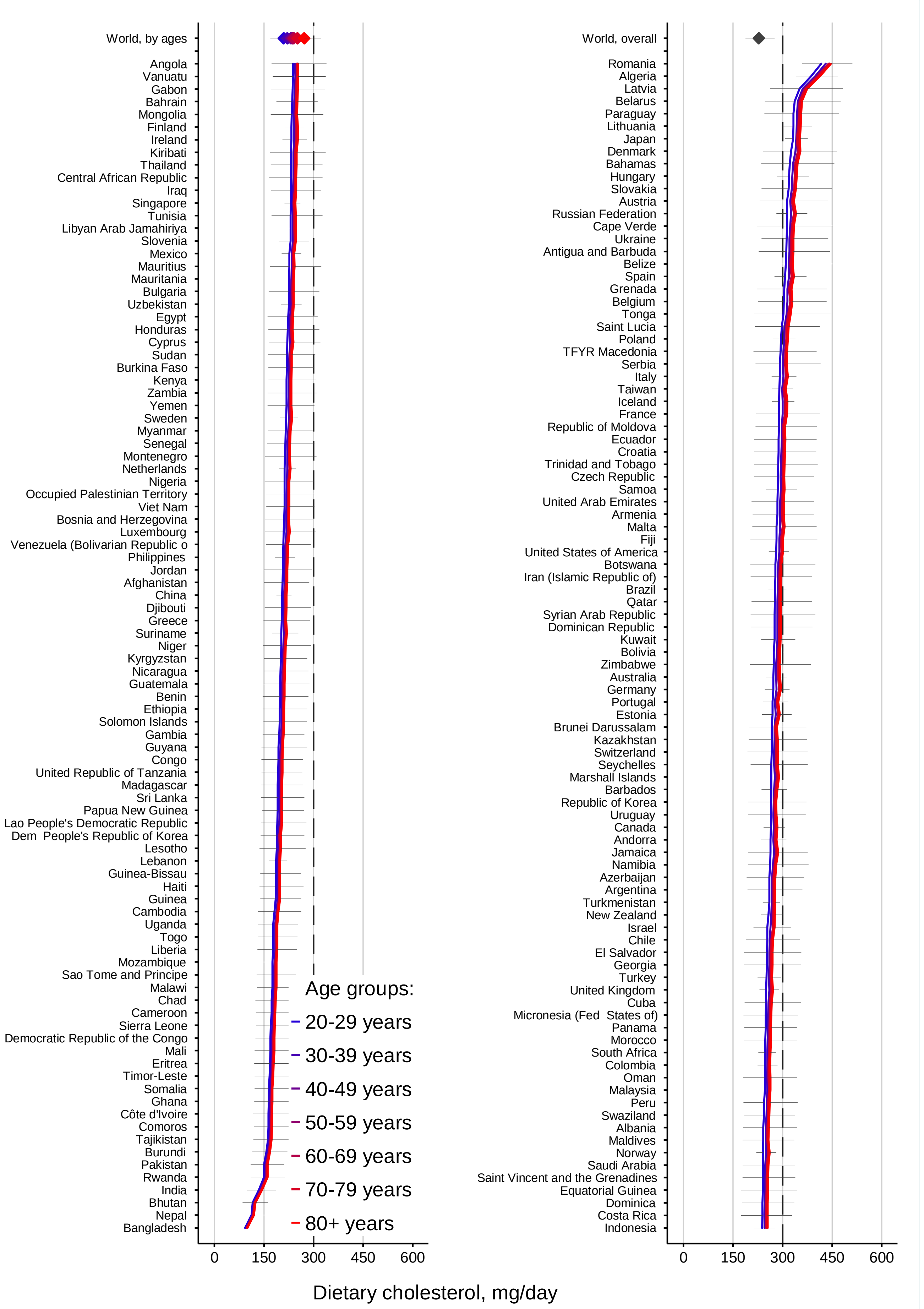

Sodium consumption among men

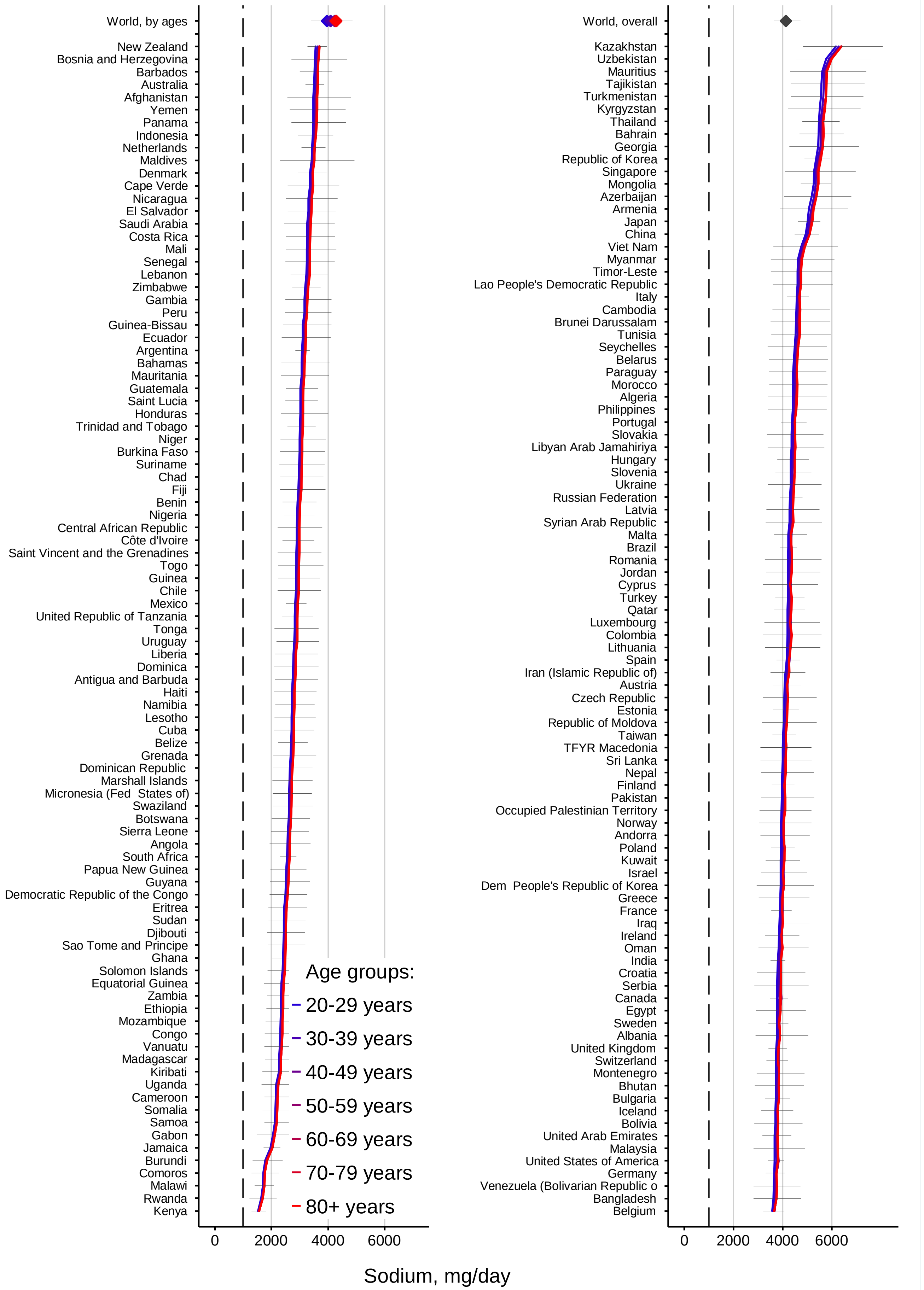

Figure S20. Sodium consumption among men and women aged 20 years or older in 187 countries.
Countries are ordered by the mean consumption levels among men and women with 20-29 years of age, from the lowest at the bottom-left to the highest at the top-right. Error bars for each country represent a lower side of 95% uncertainty interval (UI) for the lowest estimate and an upper side of 95% UI for the highest estimate. The dashed vertical line represents mean of the theoretical minimal risk exposure distribution for sodium consumption.

Sodium consumption among women

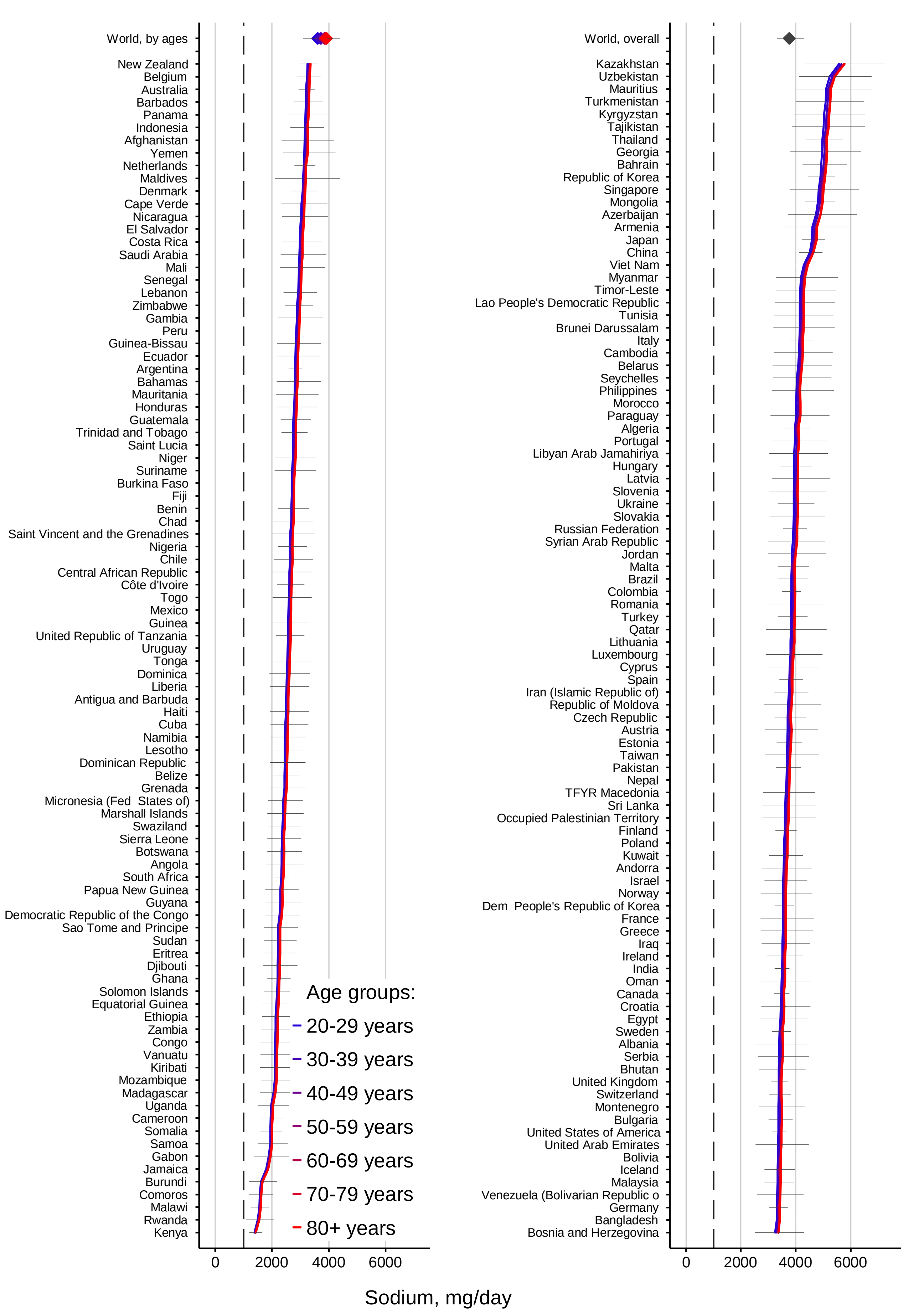

Dietary patterns based on more healthful foods/nutrients, men

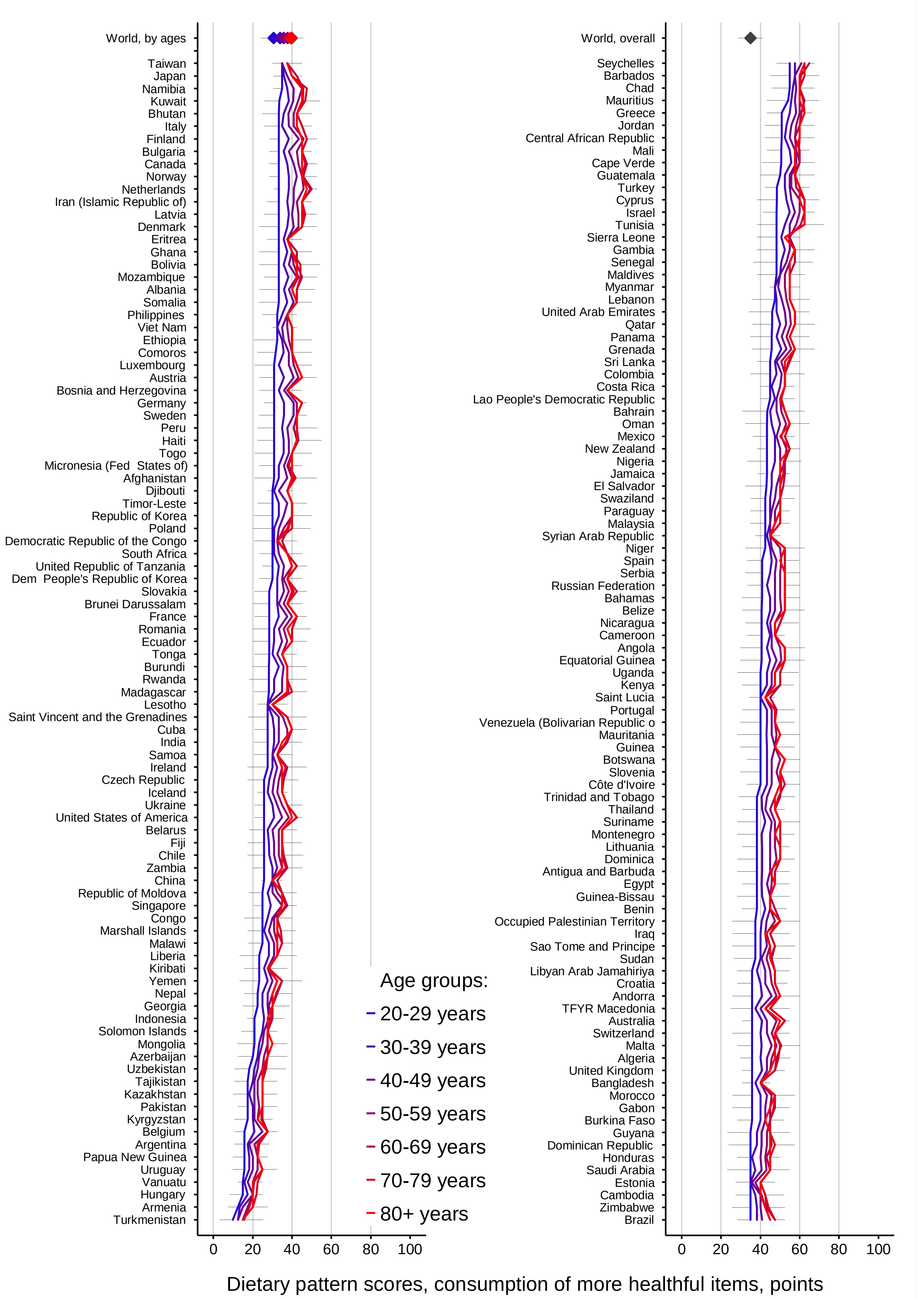

Figure S21. Dietary patterns based on consumption of more healthful foods and nutrients among men and women aged 20 years or older in 187 countries. Countries are ordered by the scores of the group of 20-29 years of age, from the lowest at the bottom-left to the highest at the top-right. Error bars represent a lower side of 95% uncertainty interval (UI) for the lowest estimate and an upper side of 95% UI for the highest estimate. The dashed vertical line represents mean of the theoretical minimal risk exposure distribution for sodium consumption.

Dietary patterns based on more healthful foods/nutrients, women

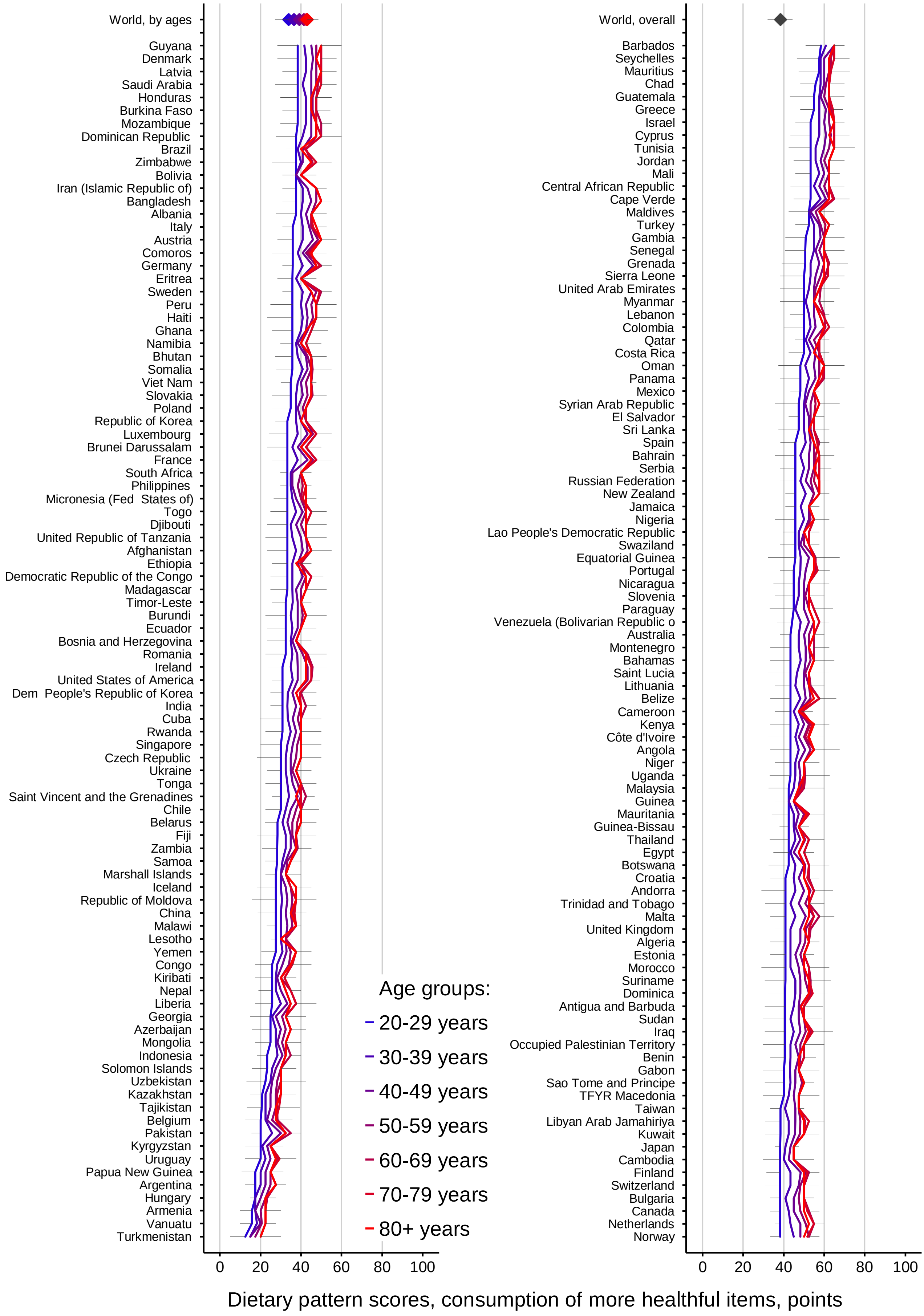

Dietary patterns on fewer unhealthful foods/nutrients, men

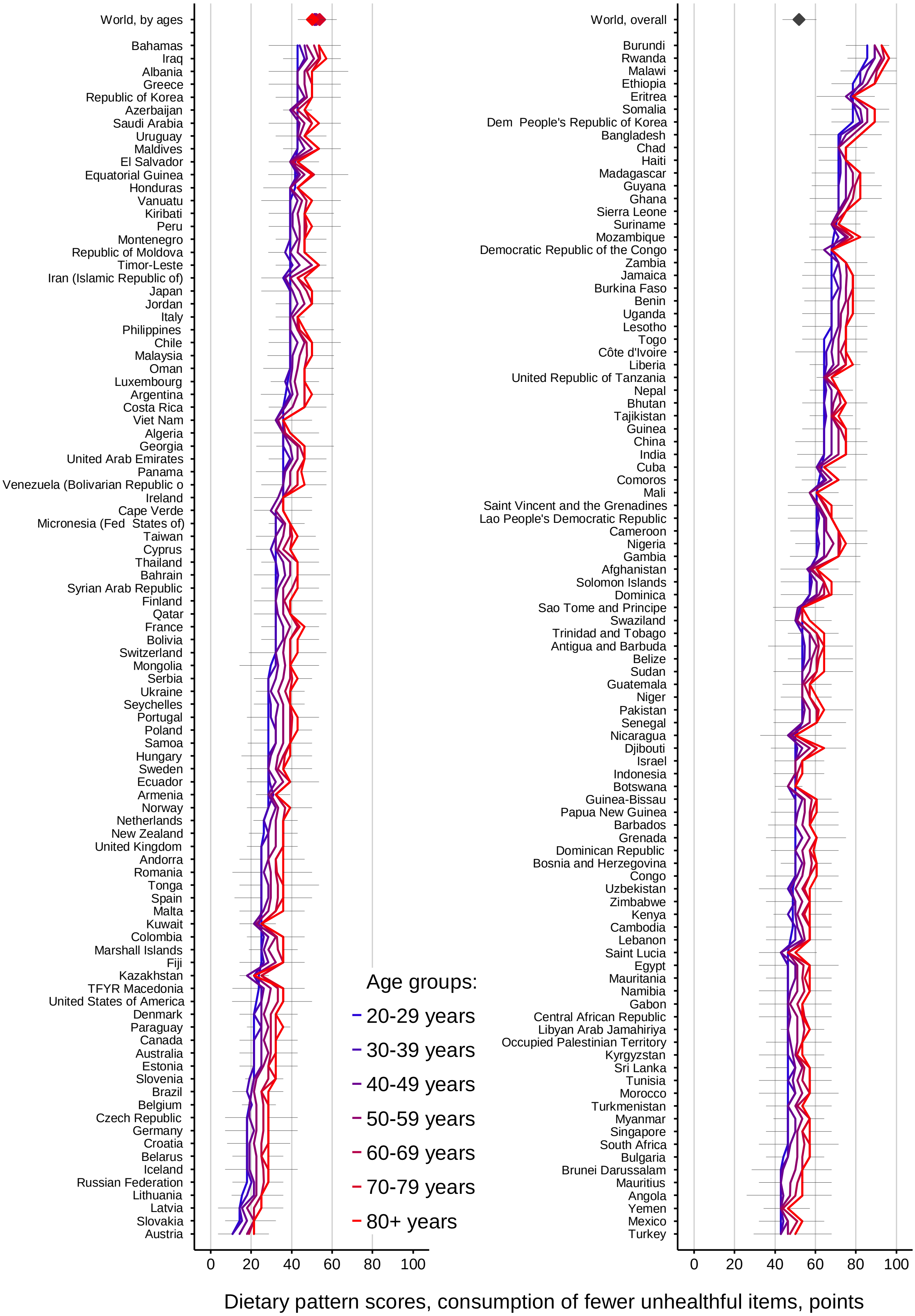

Figure S22. Dietary patterns based on consumption of lesser unhealthful foods and nutrients among men and women aged 20 years or older in 187 countries. Countries are ordered by scores of the subgroups of 20-29 years of age, rom the lowest at the bottom-left to the highest at the top-right. Error bars for each country represent a lower side of 95% uncertainty interval (UI) for the lowest estimate and an upper side of 95% UI for the highest estimate. The dashed vertical line represents mean of the theoretical minimal risk exposure distribution for sodium consumption.

Dietary patterns on fewer unhealthful foods/nutrients, women

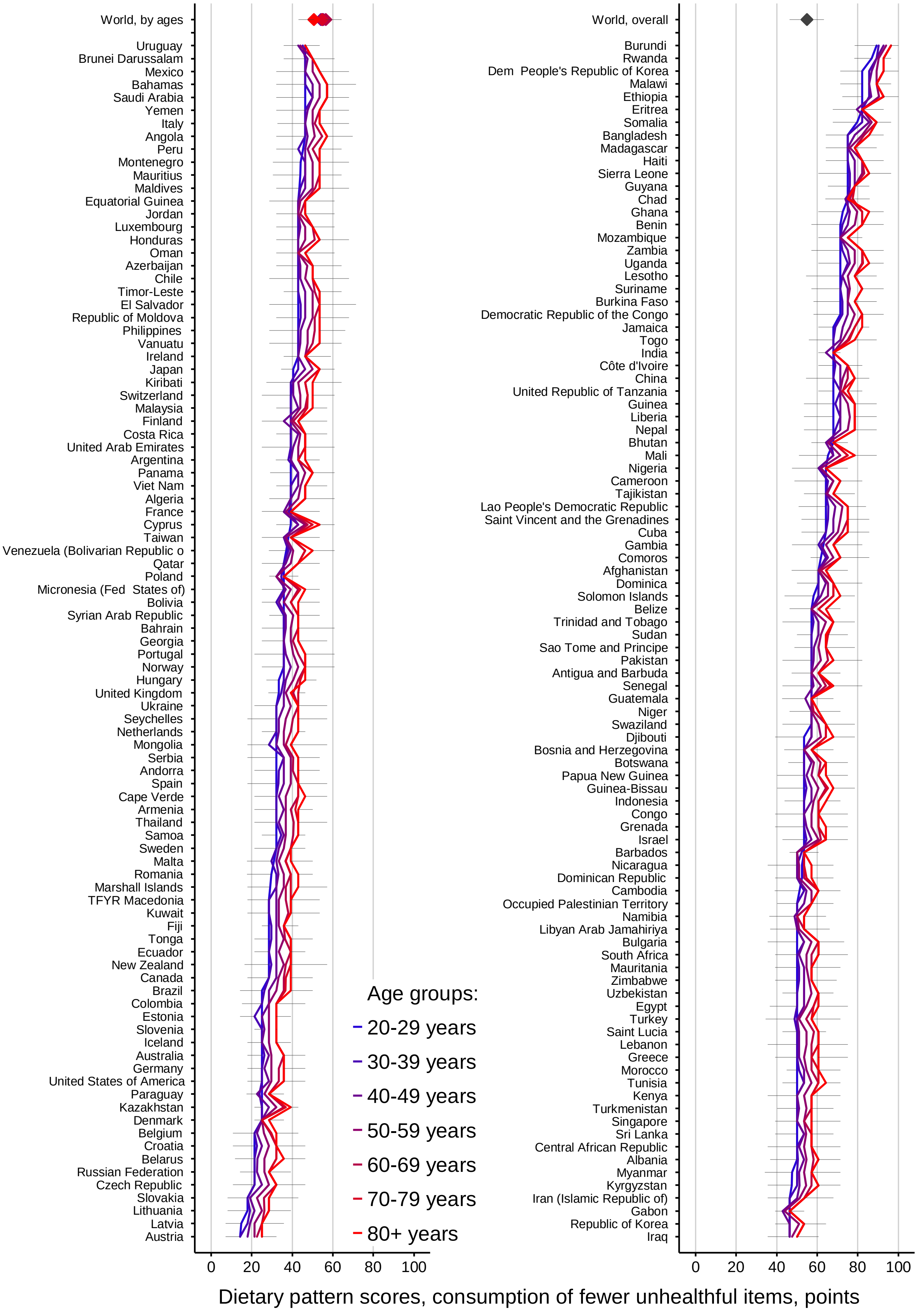

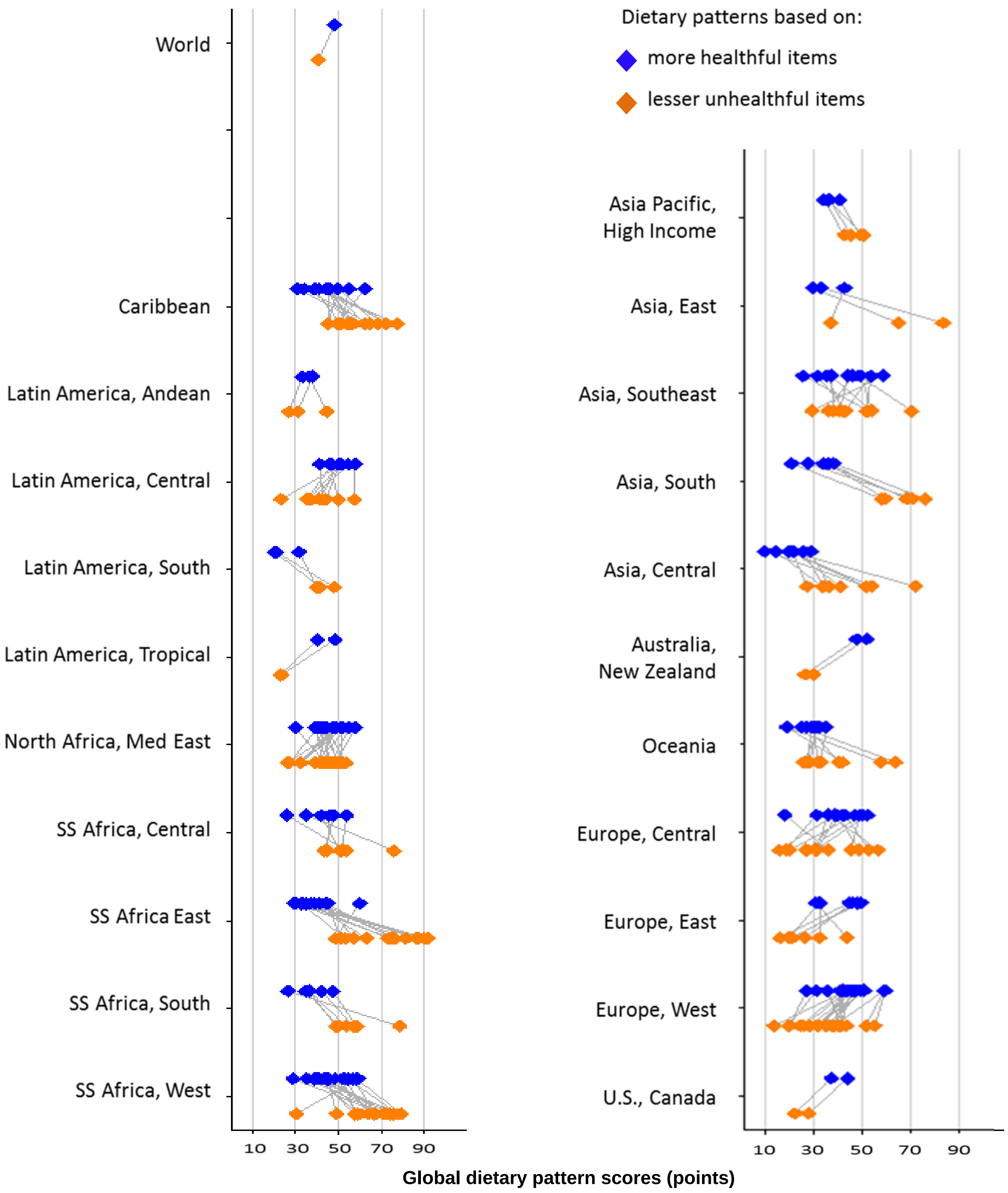

Figure S23. Global dietary patterns of 187 countries in 21 world regions. Two diet quality measures of each country are displayed. Two dots from each country are connected with a grey line. Blue represents diet quality based on greater consumption of 10 healthful foods/nutrients. Orange represents diet quality based on lesser consumption of 7 unhealthful foods/nutrients. The possible score is from 0 (the worst quality) to 100 (the best quality). SS, Sub-Saharan; U.S., United States.

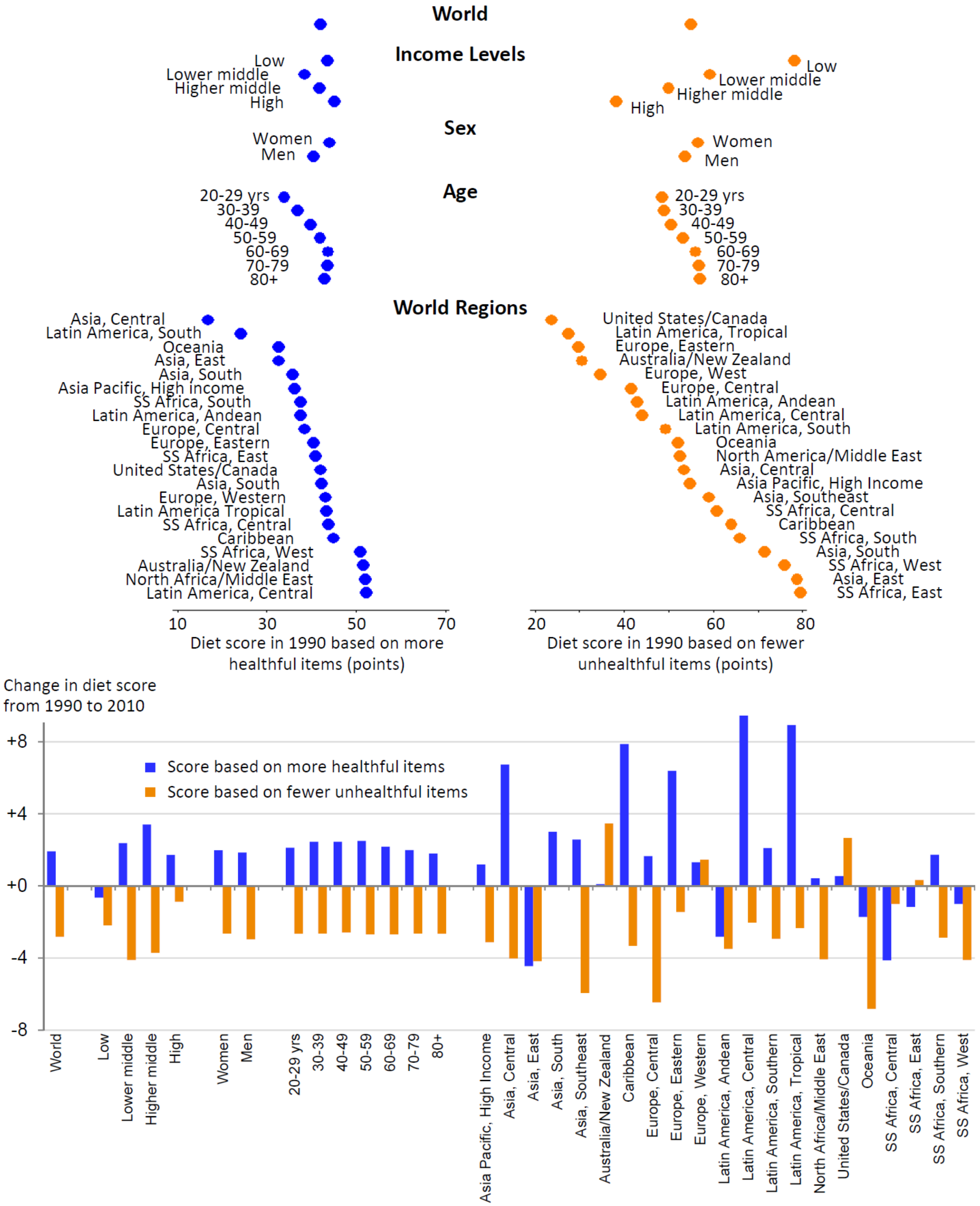

Figure S24. Dietary patterns in 1990 and change from 1990 to 2010 among men and women by demographics and world regions. Top) Diet scores based on more healthful items (left) and fewer unhealthful items (right) in 1990. Bottom) Changes in the two diet scores from 1990 to 2010. More healthful items (n=10) included fruits, vegetables, beans and legumes, nuts and seeds, whole grains, milk, polyunsaturated fatty acids, fish, plant omega-3 PUFA, and fibre. Unhealthful items (n=7) included unprocessed red meats, processed meats, sugar-sweetened beverages, saturated fat, trans fat, dietary cholesterol, and sodium. Values represent change in degrees of adherence to each dietary pattern scaled to be 0 (least healthful) to 100 (most healthful) in each year. Abbreviation: SS, Sub-Saharan.

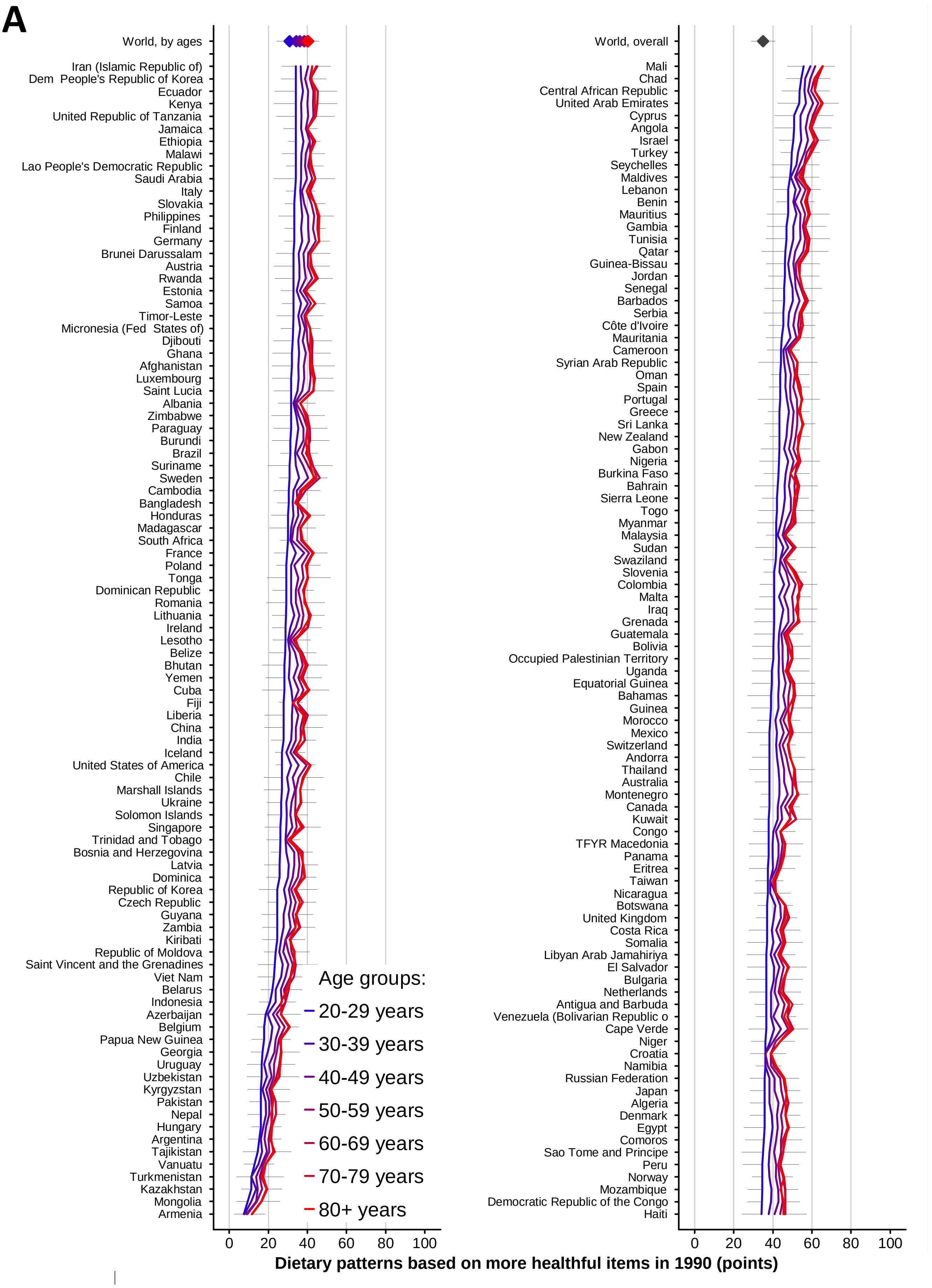

Figure S25. Dietary patterns in 1990 among men and women in 187 countries. A. Dietary pattern based on higher consumption of fruits, vegetables, beans and legumes, nuts and seeds, whole grains, milk, polyunsaturated fatty acids (PUFA), fish, plant omega-3 PUFA, and Fibre. B. Dietary pattern based on fewer consumption of unprocessed red meats, processed meats, sugar-sweetened beverages, saturated fat, trans fat, dietary cholesterol, and sodium. Values represent degrees of adherence to each dietary pattern, ranging from 0 (least healthful) to 100 (most healthful). The countries are ordered by scores among adults aged 20-29 years.

B

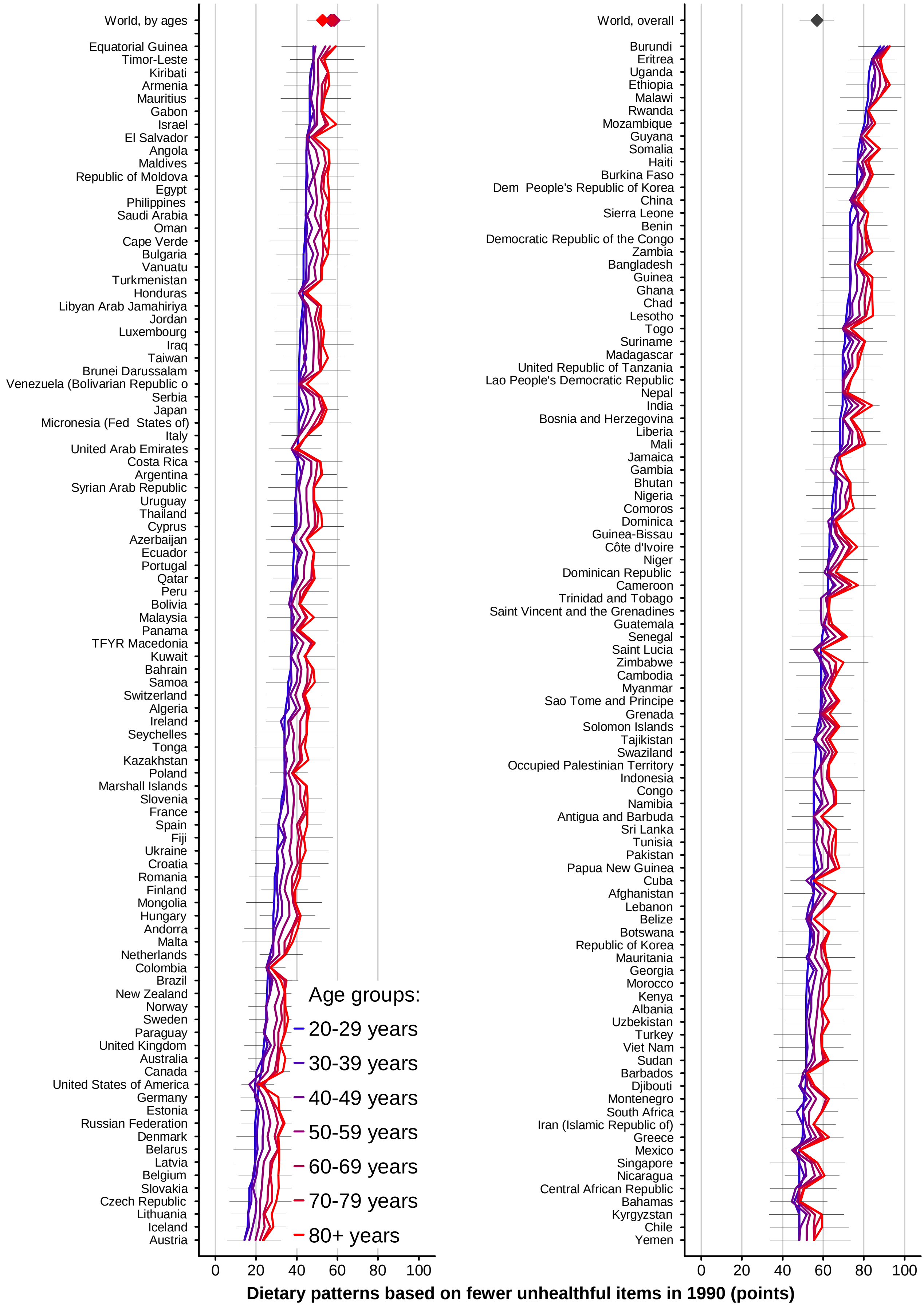
World, by ages
Equatorial Guinea
Timor-Leste
Kiribati
Armenia
Mauritius
Gabon
Israel
El Salvador
Angola
Maldives
Republic of Moldova
Egypt
Philippines
Saudi Arabia
Oman
Cape Verde
Bulgaria
Vanuatu
Turkmenistan
Honduras
Libyan Arab Jamahiriya
Jordan
Luxembourg
Iraq
Taiwan
Brunei Darussalam
Venezuela (Bolivarian Republic o
Serbia
Japan
Micronesia (Fed States of)
Italy
United Arab Emirates
Costa Rica
Argentina
Syrian Arab Republic
Uruguay
Thailand
Cyprus
Azerbaijan
Ecuador
Portugal
Qatar
Peru
Bolivia
Malaysia
Panama
TFYR Macedonia
Kuwait
Bahrain
Samoa
Switzerland
Algeria
Ireland
Seychelles
Tonga
Kazakhstan
Poland
Marshall Islands
Slovenia
France
Spain
Fiji
Ukraine
Croatia
Romania
Finland
Mongolia
Hungary
Andorra
Malta
Netherlands
Colombia
Brazil
New Zealand
Norway
Sweden
Paraguay
United Kingdom
Australia
Canada
United States of America
Germany
Estonia
Russian Federation
Denmark
Belarus
Latvia
Belgium
Slovakia
Czech Republic
Lithuania
Iceland
Austria
Age groups:
20-29 years
30-39 years
40-49 years
50-59 years
60-69 years
70-79 years
80+ years
0
20
40
60
80
100
World, overall
Burundi
Eritrea
Uganda
Ethiopia
Malawi
Rwanda
Mozambique
Guyana
Somalia
Haiti
Burkina Faso
Dem People's Republic of Korea
China
Sierra Leone
Benin
Democratic Republic of the Congo
Zambia
Bangladesh
Guinea
Ghana
Chad
Lesotho
Togo
Suriname
Madagascar
United Republic of Tanzania
Lao People's Democratic Republic
Nepal
India
Bosnia and Herzegovina
Liberia
Mali
Jamaica
Gambia
Bhutan
Nigeria
Comoros
Dominica
Guinea-Bissau
Côte d'Ivoire
Niger
Dominican Republic
Cameroon
Trinidad and Tobago
Saint Vincent and the Grenadines
Guatemala
Senegal
Saint Lucia
Zimbabwe
Cambodia
Myanmar
Sao Tome and Principe
Grenada
Solomon Islands
Tajikistan
Swaziland
Occupied Palestinian Territory
Indonesia
Congo
Namibia
Antigua and Barbuda
Sri Lanka
Tunisia
Pakistan
Papua New Guinea
Cuba
Afghanistan
Lebanon
Belize
Botswana
Republic of Korea
Mauritania
Georgia
Morocco
Kenya
Albania
Uzbekistan
Turkey
Viet Nam
Sudan
Barbados
Djibouti
Montenegro
South Africa
Iran (Islamic Republic of)
Greece
Mexico
Singapore
Nicaragua
Central African Republic
Bahamas
Kyrgyzstan
Chile
Yemen
0
20
40
60
80
100
Dietary patterns based on fewer unhealthful items in 1990 (points)

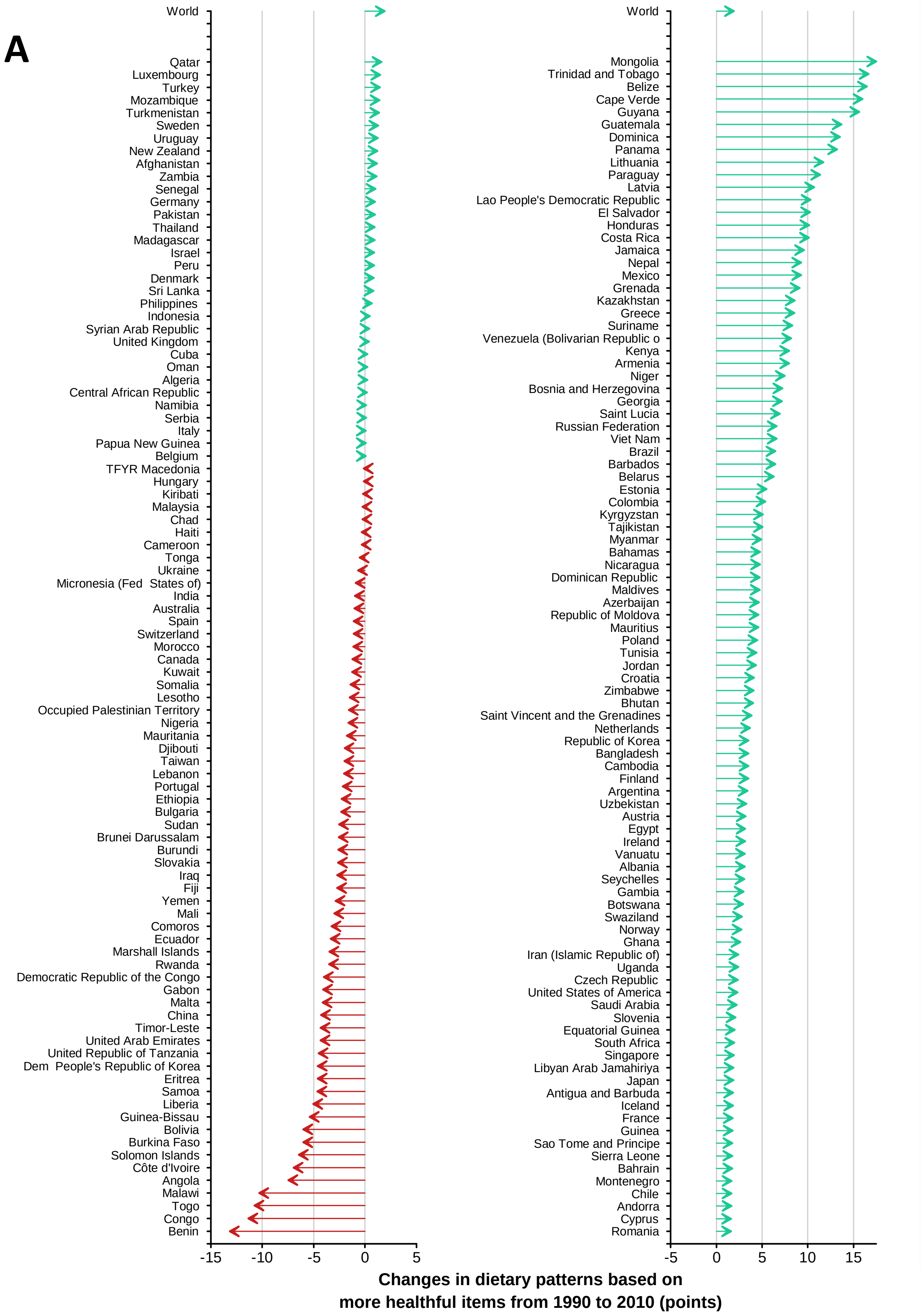

Figure S26. Changes in dietary patterns from 1990 to 2010 among men and women in 187 countries. A. Changes in dietary pattern based on higher consumption of fruits, vegetables, beans and legumes, nuts and seeds, whole grains, milk, polyunsaturated fatty acids (PUFA), fish, plant omega-3 PUFA, and fibre. B. Changes in dietary pattern based on fewer consumption of unprocessed red meats, processed meats, sugar-sweetened beverages, saturated fat, trans fat, dietary cholesterol, and sodium. Each change (Red=worsening; green=improvement) was calculated by subtracting a country mean score in 2010 from that in 1990; each score represented degrees of adherence to each dietary pattern ranging from 0 (least healthful) to 100 (most healthful).

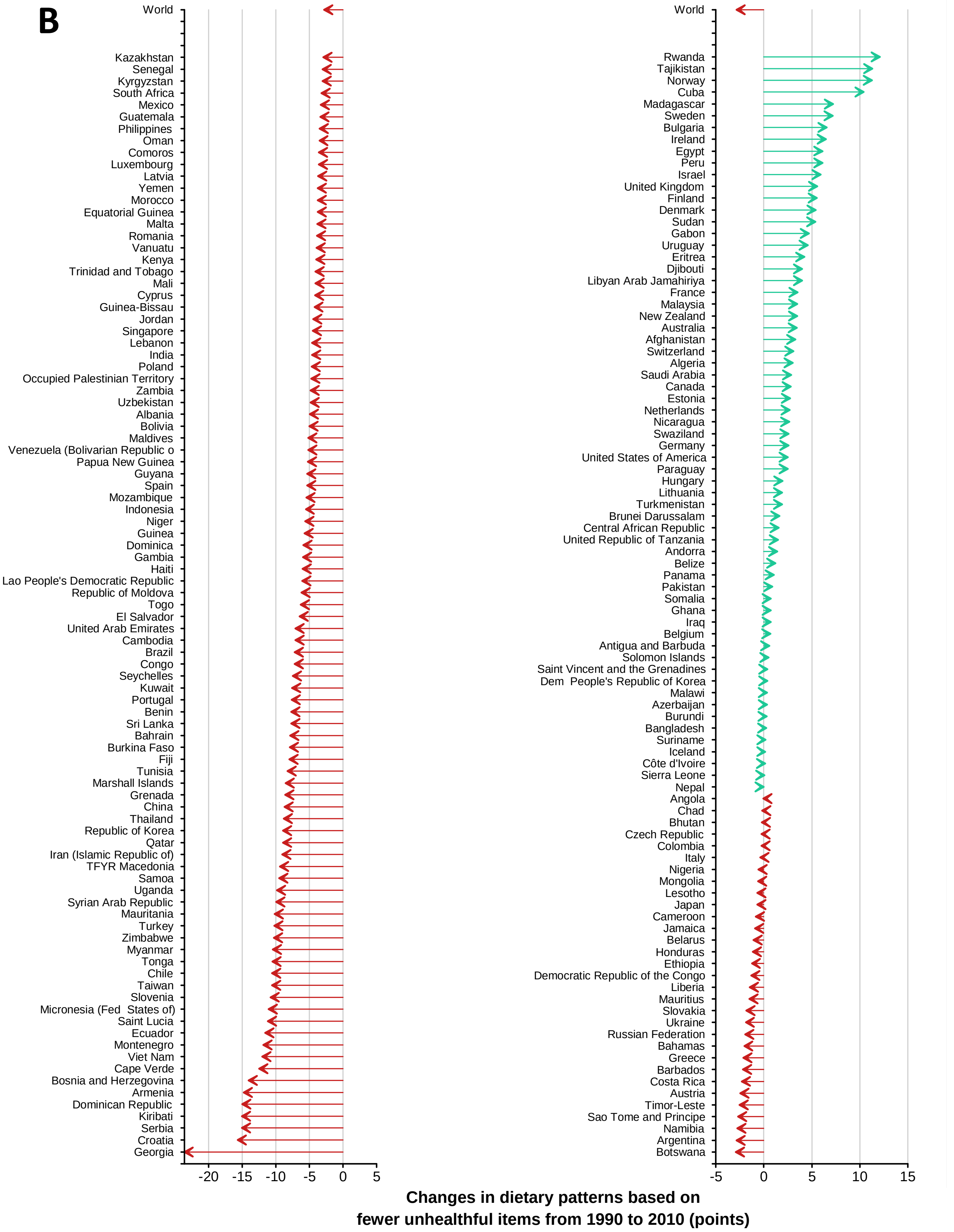
B
World
Kazakhstan
Senegal
Kyrgyzstan
South Africa
Mexico
Guatemala
Philippines
Oman
Comoros
Luxembourg
Latvia
Yemen
Morocco
Equatorial Guinea
Malta
Romania
Vanuatu
Kenya
Trinidad and Tobago
Mali
Cyprus
Guinea-Bissau
Jordan
Singapore
Lebanon
India
Poland
Occupied Palestinian Territory
Zambia
Uzbekistan
Albania
Bolivia
Maldives
Venezuela (Bolivarian Republic o
Papua New Guinea
Guyana
Spain
Mozambique
Indonesia
Niger
Guinea
Dominica
Gambia
Haiti
Lao People's Democratic Republic
Republic of Moldova
Togo
El Salvador
United Arab Emirates
Cambodia
Brazil
Congo
Seychelles
Kuwait
Portugal
Benin
Sri Lanka
Bahrain
Burkina Faso
Fiji
Tunisia
Marshall Islands
Grenada
China
Thailand
Republic of Korea
Qatar
Iran (Islamic Republic of)
TFYR Macedonia
Samoa
Uganda
Syrian Arab Republic
Mauritania
Turkey
Zimbabwe
Myanmar
Tonga
Chile
Taiwan
Slovenia
Micronesia (Fed States of)
Saint Lucia
Ecuador
Montenegro
Viet Nam
Cape Verde
Bosnia and Herzegovina
Armenia
Dominican Republic
Kiribati
Serbia
Croatia
Georgia
-20 -15 -10 -5 0 5
World
Rwanda
Tajikistan
Norway
Cuba
Madagascar
Sweden
Bulgaria
Ireland
Egypt
Peru
Israel
United Kingdom
Finland
Denmark
Sudan
Gabon
Uruguay
Eritrea
Djibouti
Libyan Arab Jamahiriya
France
Malaysia
New Zealand
Australia
Afghanistan
Switzerland
Algeria
Saudi Arabia
Canada
Estonia
Netherlands
Nicaragua
Swaziland
Germany
United States of America
Paraguay
Hungary
Lithuania
Turkmenistan
Brunei Darussalam
Central African Republic
United Republic of Tanzania
Andorra
Belize
Panama
Pakistan
Somalia
Ghana
Iraq
Belgium
Antigua and Barbuda
Solomon Islands
Saint Vincent and the Grenadines
Dem People's Republic of Korea
Malawi
Azerbaijan
Burundi
Bangladesh
Suriname
Iceland
Côte d'Ivoire
Sierra Leone
Nepal
Angola
Chad
Bhutan
Czech Republic
Colombia
Italy
Nigeria
Mongolia
Lesotho
Japan
Cameroon
Jamaica
Belarus
Honduras
Ethiopia
Democratic Republic of the Congo
Liberia
Mauritius
Slovakia
Ukraine
Russian Federation
Bahamas
Greece
Barbados
Costa Rica
Austria
Timor-Leste
Sao Tome and Principe
Namibia
Argentina
Botswana
-5 0 5 10 15
Changes in dietary patterns based on
fewer unhealthful items from 1990 to 2010 (points)

Collaborators:

Members of the Global Burden of Diseases Nutrition and Chronic Diseases Expert Group (NutriCoDE) core group: DM, SF, SK, RM, JP, PS, and Majid Ezzati, Professor, Faculty of Medicine, School of Public Health, Imperial College London, London, UK.

Other Members of NutriCoDE: FI, Ibrahim Elmadfa, PhD, Institute of Nutritional Sciences, University of Vienna, Vienna, Austria; Shadi Kalantarian, MD, MPH, Harvard School of Public Health, Boston, Massachusetts, US; Mayuree Rao, BA, The Warren Alpert Medical School of Brown University, Providence, Rhode Island, US; Pattra Wirojratana, Harvard School of Public Health, Boston, Massachusetts, US.

Members of Dietary Exposure Estimation subgroup: Stephen S Lim, PhD, Institute for Health Metrics and Evaluation, University of Washington, Seattle, Washington, US; Kathryn G Andrews, MPH, African Leaders Malaria Alliance, Dar es Salaam, Tanzania; Rebecca E Engell, BA, Institute for Health Metrics and Evaluation, University of Washington, Seattle, Washington, US.

Urine-based Sodium Exposures—Intersalt and Intermap: Paul Elliott, PhD, Department of Epidemiology and Biostatistics, School of Public Health, Imperial College London, London, UK; Ian Brown, PhD, Public Health Specialty Training Program, NHS East of England Multi-Professional Deanery, Fulbourn, UK.

Urine-based Sodium Exposures—corresponding members: John Britton, PhD, Division of Epidemiology and Public Health, University of Nottingham, Nottingham, UK; Andrew Fogarty, PhD, Division of Epidemiology and Public Health, University of Nottingham, Nottingham, UK; Mary Anne Land, MPH, doctoral student, The George Institute of Global Health, University of Sydney, Sydney, Australia; Sarah Lewis, PhD, Division of Epidemiology and Public Health, University of Nottingham, Nottingham, UK; Tricia McKeever, PhD, School of Community Health Sciences, University of Nottingham, Nottingham, UK; Bruce Neal, PhD, The George Institute of Global Health, University of Sydney, Sydney, Australia; Marga C Ocké, PhD, National Institute for Public Health and the Environment (RIVM), PO Box 1, 3720 BA Bilthoven, The Netherlands; Jacqui Webster, PhD, The George Institute of Global Health, University of Sydney, Sydney, Australia.

Dietary Exposures—corresponding members: Pamela A Abbott, PhD, University of Aberdeen, UK; Morteza Abdollahi, MD, MPH, National Nutrition and Food Technology Research Institute, Iran, Iran; Enrique O Abeyá Gilardon, MD, MPH, Ministerio de Salud, Argentina; Habibul Ahsan, MD, University of Chicago, US; Mohannad Abed Alfattah Al Nsour, MD, Eastern Mediterranean Public Health Network (EMPHNET), Jordan; Suad N Al-Hooti, MSc, Kuwait Institute for Scientific Research, Kuwait; Carukshi Arambepola, MD, Faculty of Medicine, University of Colombo, Sri Lanka; Hubert Barennes, PhD, Institut Francophone pour la Médecine Tropicale, Lao PDR; Simon Barquera, PhD, Instituto Nacional de Salud Publica (INSP), Mexico; Ana Baylin, MD, DrPH, University of Michigan, US; Wulf Becker, PhD, professor, National Food Agency, Sweden; Dr Peter Bjerregaard, MD, Medical Science, National Institute of Public Health, University of Southern Denmark, Denmark; Lesley T Bourne, PhD, Environment and Health Research Unit, Medical Research Council, South Africa; Neville Calleja, MD MFPH, Department of Health Information and Research, Malta; Mario V Capanzana, PhD, Food and Nutrition Research Institute, Philippines; Katia Castetbon, PhD, Institut de veille sanitaire, France; Hsing-Yi Chang, DrPH, National Health Research Institutes, Taiwan; Yu Chen, PhD, New York University School of Medicine, US; Melanie J Cowan, MPH, WHO, Switzerland; Professor Stefaan De Henauw, MD, PhD, Ghent University, Department of Public Health, Belgium; Eric L Ding, ScD, Harvard Medical School and Harvard School of Public Health, US; Charmaine A Duante, MSc, Food and Nutrition Research Institute-Department of Science and Technology, Philippines; Pablo Duran, PhD, Dirección Nacional de Maternidad e Infancia, Ministerio de Salud de la Nación, Argentina; Professor Ibrahim Elmadfa, Institute of Nutritional Sciences, University of Vienna, Austria; Heléne Enghardt Barbieri; Farshad Farzadfar, MD, DSc, Tehran University of Medical Sciences, Iran; Dulitha N Fernando, PhD, Faculty of Medicine, University of Colombo, Sri Lanka; Aida Filipovic Hadziomeragic, MD, MSc, Institute of Public Health of Federation of Bosnia and Herzegovina, Bosnia and Herzegovina; Regina M Fisberg, PhD, Faculty of Public Health—University of São Paulo, Brazil; Simon Forsyth; Didier Garriguet, Msc, Statistics Canada, Canada; Jean-Michel Gaspoz, MD, MPH, Geneva University Hospitals and Faculty of Medicine of Geneva, Switzerland, Switzerland; Dorothy Gauci, Post Graduate Diploma (Epidemiology), Department of Health Information and Research, Malta; Brahmam NV Ginnela, MB, BS, DPH, Scientist, at National Institute of Nutrition, Indian Council of Medical Research, India; Idris Guessous, MD, Geneva University Hospitals, Switzerland; Martin C Gulliford, FFPH, King's College London, UK; Wilbur Hadden, Christian Haerpfer, PhD, University of Aberdeen, UK; Daniel J Hoffman, PhS, Rutgers, the State University of New Jersey, US; Anahita Houshiar-rad, MSC, National Nutrition and Food Technology Research Institute Shahid Beheshti University of Medical Sciences Tehran, Iran, IRIran; Inge Huybrechts, PhD, Ghent University, Department of Public Health, Belgium; Nahla C Hwalla, PhD, American University of Beirut, Lebanon; Hajah Masni Ibrahim, Master, Ministry of Health, Brunei; Manami Inoue, MD, PhD, Epidemiology and Prevention Division, Research Center for Cancer Prevention and Screening, National Cancer Center, Japan; Maria D Jackson, PhD, University of the West Indies, Jamaica; Lars Johansson, PhD, Norwegian Directorate of Health, Norway; Lital

Keinan-Boker, MD, PhD, Ministry of Health, Israel; Cho-il Kim, PhD, Korea Health Industry Development Institute, Republic of Korea; Assistance Professor Eda Koksal, PhD, Gazi University, Turkey; Hae-Jeung Lee; Yanping Li, PhD, Harvard School of Public Health, US; Nur Indrawaty Lipoeto, PhD, Andalas University, Indonesia; Guansheng Ma, PhD, National Institute for Nutrition and Food Safety, Chinese Center for Disease Control and Prevention, China; Guadalupe L Mangialavori, MS RD, Ministerio de Salud de la Nación (National Health Ministry), Argentina; Yasuhiro Matsumura, PhD, Bunkyo University, Japan; Stephen T. McGarvey, PhD, Brown University, US; Chan Mei Fen; Gert BM Mensink, PhD, Robert Koch Institute, Germany; Rafael A Monge-Rojas, PhD, Costa Rican Institute for Research and Education and Nutrition and Health (INCIENSA), Costa Rica; Abdulrahman Obaid. MUSiger, PhD, Arab Center for Nutrition, Bahrain; Balakrishna Nagalla, PhD, Scientist at National Institute of Nuyrition, India, Hyderabad; Androniki Naska, PhD, Department of Hygiene, Epidemiology and Medical Statistics, University of Athens Medical School, Greece; Marga C Ocke, PhD, National Institute for Public Health and the Environment, the Netherlands; Maciej Oltarzewski, MSc, National Food and Nutrition Institute, Poland; Philippos Orfanos, MSc, Department of Hygiene, Epidemiology and Medical Statistics, University of Athens Medical School, Greece; Marja-Leena Ovaskainen, PhD, National Institute for Health and Welfare, Finland; Wen-Harn Pan, PhD, Division of Preventive Medicine and Health Services Research, Institute of Population Health Sciences, National Health Reserch Institutes, Taiwan; Demosthenes B Panagiotakos, PhD, Harokopio University, Greece; Gulden Ayla Pekcan, PhD, Hacettepe University Department of Nutrition and Dietetics, Turkey; Stefka Petrova, MD, PhD, National Center of Public Health and Analyses, Bulgaria; Noppawan Piaseu, PhD, Mahidol University, Thailand; Christos Pitsavos, MD, Athens University Medical School, Greece; Luz Gladys. Posada, RD, Master of science, Universidad de Antioquia, Colombia; Leanne M Riley, MSc, WHO, Switzerland; Luz Maria Sánchez-Romero, MD, MSc, National Institute of Public Health, Mexico; Rusidah BT Selamat, MSc, Nutrition Division, Ministry of Health Malaysia, Putrajaya, Malaysia; Sangita Sharma; Abla Mehio Sibai, PhD, American University of Beirut—Faculty of Health Sciences, Lebanon; Rosely Sichieri, MD, PhD, State University of Rio de Janeiro, Brazil; Chansimaly Simmala, MD, Institut of Tropical Medecin, Laos; Laufey Steingrimsdottir, PhD, Professor, Iceland; Gillian Swan; Elz˙bieta Halina. Sygnowska, MSc, PhD, National Institute of Cardiology, Poland; Lucjan Szponar, MD, PhD, National Food and Nutrition Institute, Poland; Heli Tapanainen, MSc, National Institute for Health and Welfare, Finland; Robert Templeton; Anastasia Thanopoulou, MD, PhD, Diabetes Center, 2nd Department of Internal Medicine, National University of Athens, Hippokration General Hospital, Greece; Holmfridur Thorgeirsdóttir, MSc, Directorate of Health, Iceland; Inga Thorsdottir; Antonia Trichopoulou, MD, Hellenic Health Foundation, Greece; Shoichiro Tsugane, MD, PhD, National Cancer Center, Japan; Aida Turrini, MS, Agricultural Research Council, Research Center on Food and Nutrition, Italy; Sirje Vaask, PhD, Tallinn University of Technology, Estonia; Coline van Oosterhout, National Institute for Public Health and the Environment, The Netherlands; J Lennert Veerman, PhD, The University of Queensland, Australia; Nowak Verena; Anna Waskiewicz, MSc, PhD, Institute of Cardiology, Department of Cardiovascular Diseases Epidemiology, Prevention and Health Promotion, Poland; Sahar Zaghloul, PhD, National Nutrition Institute, Egypt; Gábor Zajkás, MD, National Institute of Food and Nutrition Sciences, Hungary.
